# Association between Dietary Fiber Intake and Incidence of Depression and Anxiety in Patients with Essential Hypertension

**DOI:** 10.3390/nu13114159

**Published:** 2021-11-20

**Authors:** Yuanyuan Liu, Yang Ju, Lingling Cui, Ting Liu, Yunying Hou, Qing Wu, Omorogieva Ojo, Xiaojiao Du, Xiaohua Wang

**Affiliations:** 1 School of Nursing, Medical College, Soochow University, Suzhou 215006, China; 20195231002@stu.suda.edu.cn (Y.L.); wanglili83476@suda.edu.cn (Y.J.); 20185231001@stu.suda.edu.cn (L.C.); lt322426@163.com (T.L.); houyunying@suda.edu.cn (Y.H.); qwu@suda.edu.cn (Q.W.); duxiaojiao@suda.edu.cn (X.D.); 2 School of Health Sciences, Faculty of Education, Health and Human Sciences, University of Greenwich, London SE9 2UG, UK; o.ojo@greenwich.ac.uk

**Keywords:** dietary fiber, anxiety, depression, essential hypertension

## Abstract

(1) Background: Our previous study found that the dietary fiber supplement in patients with hypertension increased SCFA-producers, Bififidobacterium and Spirillum in the gut microbiota, which may be associated with improvement of depression and anxiety through the gut–brain axis. However, only a few studies have explored the association between dietary fiber intake (DFI) and the incidence of depression and anxiety in hypertensive patients. (2) Methods: A cross-sectional survey was conducted in one comprehensive hospital and one community clinic aimed at understanding the status of DFI and the association between DFI and incidences of depression and anxiety in hypertensive patients. Levels of DFI were obtained through a two-24 h diet recall. According to the levels of DFI from low to high, the participants were divided into Q1, Q2, Q3 and Q4 groups. The Reported Outcomes Measurement Information System short form v1.0-Depression 8b and Anxiety 8a were used to assess patients’ levels of depression and anxiety. (3) Results: A total of 459 hypertensive patients were recruited and the daily DFI was 10.4 g. The incidences of hypertension combined with depression and anxiety were 19.6% and 18.5%, respectively. Regression analysis showed statistically significant associations between DFI and depression (B = −0.346, *p* = 0.001) and anxiety score (B = −0.565, *p* < 0.001). In logistic regression, after the covariates were adjusted, DFI was associated with the incidence of depression in Q3 (OR 2.641, 95% CI 1.050–6.640) and with that of anxiety in Q1 (OR 2.757, 95% CI 1.035–7.346), compared with Q4. (4) Conclusions: A higher consumption of DF was a protective factor for depression and anxiety in hypertensive patients.

## 1. Introduction

Hypertension and psychological comorbidities may share some common pathological mechanisms including the disorder of intestinal flora, high inflammatory response, and the excessive activation of the renin–angiotensin–aldosterone system (RAAS) [1,2,3]. So they are closely related, and often influence or even cause each other [3]. The incidence of negative emotions in hypertensive patients is significantly higher than that of non-hypertensive people [4]. Carroll et al. [5] found that the prevalences of depression and anxiety in hypertensive patients were 9% and 12%, respectively. In China, the prevalences of depression, anxiety, depression and anxiety comorbidities in patients with hypertension were as high as 47.9%, 63% and 36.4% [6].

Psychological comorbidities have been linked to worse cardiovascular outcomes [7]. An association between anxiety and the occurrence of coronary heart disease and cardiac death, showed that anxious persons were at risk of coronary heart disease (HR, 1.26; 95% CI, 1.15–1.38) and cardiac death (HR, 1.48; 95% CI, 1.14–1.92) [8]. Abramson et al. [9] found that depression was an independent prevalence factor that increases the risk of heart failure (OR, 2.59; 95% CI, 1.57–4.27). In China, hypertensive patients combined with anxiety, depression had lower life quality [10]. Therefore, management of mental health is an important aspect of hypertension treatment [7].

Recently, there has been increasing evidence that insufficient dietary fiber intake (DFI) influences psychological health [11,12,13]. Dietary fibers comprise the cell walls of plants that are resistant to digestion by human enzymes but might be degraded by resident gut microbial enzymes to produce short-chain fatty acids (SCFAs) [11,12]. SCFAs regulate emotions through the intestinal–brain axis and inhibit inflammation [14]. A study conducted which included participants aged from 12 to 18 in Iran found that higher DF intake is a protective factor for depressive symptoms (OR:0.42, 95% CI: 0.25~0.68) [15]. Taylor et al. [12] summarized that the consumption of probiotics (a type of dietary fiber) improved psychological or biological measures of depression and anxiety. Andrew et al. [11] showed that participants who consumed cereal bars (each containing 1.1 g DF) felt less anxious than those who did not consume them. However, these studies did not focus on hypertensive patients with depression and anxiety. Xu et al. [16] found that intakes of total fiber were inversely associated with depressive symptoms in patients with chronic diseases including hypertension. Miki [17] found that dietary fiber intake from vegetables and fruits was significantly inversely associated with depressive symptoms in patients with cardiovascular diseases, cancer, and so on. Our previous study published in 2020 found that the dietary fiber (oat bran) supplement in patients with hypertension increased SCFA-producers, Bififidobacterium and Spirillum in the gut microbiota [18]. Therefore, we hypothesized that insufficient DF intake is one of the risk factors for the high incidence of psychological problems in hypertensive patients. This study aimed to investigate the status of DF intake and explore the associations between DF intake and psychological comorbidities in hypertensive patients.

## 2. Materials and Methods

### 2.1. Study Design

This study was a cross-sectional investigation. The patients with hypertension who met the criteria for inclusion were selected for investigation through convenient sampling from July 2019 to October 2020 in the First Affiliated Hospital of Soochow University and Jinchang Community. The study was approved by the ethics committee of The First Affiliated Hospital of Soochow University (ECSU-2019000148).

### 2.2. Study Population

Inclusion criteria: (1) diagnosed with hypertension according to the “2018 Chinese Guidelines for Prevention and Treatment of Hypertension—A report of the Revision Committee of Chinese Guidelines for Prevention and Treatment of Hypertension” [19]; (2) age ≥ 18 years old; (3) could complete the questionnaire independently or with the help of researchers; (4) informed consent and voluntary participation. Exclusion criteria: (1) were secondary hypertension patients; (2) had severe physical comorbidities or complications (e.g., malignant tumors, severe liver and kidney damage); (3) received psychotherapy within the past one month; (4) had cognitive dysfunction; (5) had diarrhea, dysentery or other gastrointestinal diseases within the past one month; (6) were pregnant or lactating women; (7) participating in other research.

### 2.3. Office Blood Pressure

Blood pressure was measured using the corrected electronic sphygmomanometer (Omron HEM-8102A, Omron Corporation, Shanghai, Japan) in a community service room or a hospital demonstration classroom, including office systolic blood pressure (oSBP) and office diastolic blood pressure (oDBP). The researchers measured participants’ BP of the upper arm at sitting position after participants taking a rest for at least 5 min. Neither participant nor researcher should talk before, during and between measurements. Repeated measurement was performed with a 5 min interval. We took the average of these two values as the final BP value.

### 2.4. Dietary Fiber Intake Record

Dietary intake data were obtained through two 24-h diet recall [20]. A 24-h diet recall method is a review and description of all food consumed during the day before the survey. In order to better help patients evaluate specific food portions, the researchers distributed a brochure that contained the pictures of food standard portions [originated Chinese Dietary Guidelines (2016 Edition)] before collecting dietary data [21]. The first dietary data were collected face-to-face after collection of demographic and clinical indicators. Other dietary data were collected by telephone 2 or 3 days later. The quantities of nutrient intake were calculated using the Beijing Feihua Nutrition Software (V2.7.6.10, Beijing, China) and adopted as the average quantities of nutrients of two recording days. According to the quartile grouping of DF intake from low to high, the participants were divided into Q1, Q2, Q3 and Q4, respectively.

### 2.5. Depression and Anxiety

The psychological comorbidities mainly referred to anxiety and depression in this study. Reported Outcomes Measurement Information System (PROMIS) short form v1.0-Depression 8b and Anxiety 8a were used to assess past 7-day severity of depressive symptoms and anxious symptoms, which were permitted by C2008-2019 PROMIS Health Organization (PHO). Both item banks contain 8 items, using a 5-point rating scale that ranges from 1 (“Never”) to 5 (“Always”). The total score of the scale is the sum of the scores of all items [22]. According to the scoring manual, the total score was converted to t score, which was used as the final score of the scale. The total scores ranged from 37.1 to 81.1 points [23]. The higher the score of the scale, the more serious the depressive or anxiety symptoms are, and a score > 55 is considered as depression or anxiety [22]. The Cronbach α coefficients of PROMIS short form v1.0-Depression 8b and Anxiety 8a were 0.897 and 0.917 in this study, indicating that both tools had good reliability.

### 2.6. Demographic and Clinical Information

A set of general questionnaires which included age, sex, marital status, educational level, occupational status, medical payment, exercise, duration of sleep, quality of sleep, smoking status, alcohol drinking, body mass index (BMI), waist-to-hip ratio (WHR), constipation, duration of hypertension, taking antihypertensive drugs, complication, comorbidity were administered and assessed via a survey. Body height and weight were measured to the nearest 0.1 cm and 0.1 kg, respectively, in a standardized procedure with participants wearing light clothes and without shoes. BMI was calculated as weight (in kilograms) divided by the square of height (in meters). Exercise less than 3 times a week, less than 20 min each time, and continuous time less than 3 months, was considered irregular exercise, otherwise, it is exercise [24]. The quality of sleep was the subjective feeling of the patient and judged by the patient himself. Constipation usually presents with symptoms including hard or lumpy stools, reduced frequency of defaecation, a sensation of incomplete evacuation or blockage, straining at stool, and some may also report abdominal pain and bloating [25]. Hypertension complication refers to the occurrence of another disease or symptom caused by blood pressure in the development process, including stroke, cerebral hemorrhage, myocardial infarction, frequent angina, aortic dissection, renal insufficiency, heart failure, and left ventricular hypertrophy [26]. They were selected via tick boxes by patients. Comorbidity referred to the co-existence of one or more diseases or clinical conditions and is independent of blood pressure [27]. When the patient cannot determine whether his disease was a complication, the research team asked the patient for medical history to make a decision.

### 2.7. Data Collection Process

The following was the data collection process. (1) The researchers explained the purpose of the study and obtained informed consent from patients with hypertension who met the inclusion criteria. (2) The collection of patients’ data: the patients’ parameters were measured including the office blood pressure twice before and after they filled out the general questionnaire, PROMIS short form v1.0-Depression 8b and Anxiety 8a. Then the researchers checked whether the questionnaires were completely filled out. This process took about 10 min. (3) The researchers recorded the patients’ diet data on the previous day and added the patients’ WeChat or telephone numbers. After 2–3 days, the researchers obtained the second 24-h dietary data through electric interviews or WeChat.

### 2.8. Statistical Analysis

Statistical analyses were conducted using SPSS 22.0. Demographic and clinical indicators were presented as mean ± standard deviation and frequency (percentage). Use linear analysis to evaluate the associations between the quantities of dietary fiber intake and the scores of depression and anxiety. To further analyze the relationship between different doses of dietary fiber intake and depression and anxiety, we used univariate and multivariate binary logistic regression. In multivariate logistic regressions, the covariates of model 1 were *p* < 0.05 in the baseline demographics indicators including age, and occupational status. Model 2 adjusted for variables in model 1, plus clinical indicators (*p* < 0.05) including constipation, duration of hypertension, taking antihypertensive drugs, SBP and DBP. There was further adjusting of the nutritional indicators (*p* < 0.05) including energy (model 3). *p*-values < 0.05 were considered statistically significant.

## 3. Results

### 3.1. Demographic and Clinical Characteristics

Initial questionnaires were collected from a total of 471 participants. Among those collected, the remaining 459 were considered valid and the effective recovery rate was 97.5%. Participants’ socio-demographic and clinical characteristics are presented in Table 1. The range of age of participants was from 20 to 81 years. Compared with participants without depression or anxiety, those with depression or anxiety were younger, on the job, had non-constipation, shorter duration of hypertension, taking antihypertensive drugs and higher BP levels. The blood pressure control level of the depression or anxiety group was higher than that of the non-depression or anxiety group. None of the participants took anti-anxiety and anti-depression drugs. Tables S1 and S2 in the supplementary materials show the demographic and clinical characteristics from two different recruitment centers. 

### 3.2. The Status of DF Intake

The status of DF intake is presented in Table 2. The median daily intakes of DF of total participants was 10.4 g; Q1, Q2, Q3 and Q4 were 6.3 g, 9.4 g, 12.0 g and 17.8 g, respectively.

### 3.3. The Status of Depression and Anxiety

Among the patients with hypertension in this group, the median score for depression and is 52.3 and 53.2. There were 90 (19.6%) depressive cases with a median score of 58.4 and 85 (18.5%) anxiety cases with a median score of 57.1.

### 3.4. The Association between DF Intake and Incidence of Depression

Results from statistical models are represented in Table 3. Models 1~3 revealed a statistically significant association between dietary fiber intake and depression score (Model 3, B = −0.346, *p* = 0.001).

The odds ratios (OR) with 95% confidence intervals (CI) of the incidence of depression based on DF intake are shown in Table 4. Taking Q4 as a reference group in univariate logistic regression, Q1, Q2 and Q3 groups were significantly associated with the incidences of depression. After the covariates were adjusted for demographics indicators and clinical indicators (*p* < 0.05), the results remained statistically significant. After further adjustment for nutrients intake (model 3), Q3 was still significantly associated with the incidence of depression, with an OR value of 2.641 (1.050–6.640), whereas the associations between the DF intakes in Q1 and Q2 groups with the incidences of depression were not statistically significant.

### 3.5. The Association between DF Intake and Incidence of Anxiety

Results from statistical models are represented in Table 5. Models 1~3 revealed a statistically significant association between dietary fiber intake and the score of anxiety (Model 3, B = −0.565, *p* < 0.001).

The OR with 95% CI of the incidence of anxiety based on DF intakes is shown in Table 6. Taking Q4 as a reference group in univariate and multivariate logistic regression, only Q1 was significantly associated with the incidence of anxiety. After further adjustment for covariates (model 1, model 2 and model 3), the OR (95% CI) were 0.619 (0.471−0.812), 3.469 (1.458–8.250) and 2.757 (1.035−7.346), respectively.

## 4. Discussion

A total of 459 patients with hypertension were recruited for this study, and the average age was 51.3 ± 12.6 years. A total of 288 (62.7%) of the participants were males, which is consistent with the investigation report of hypertension epidemiology in China [5,28]. Compared with participants without depression or anxiety, those with depression or anxiety were younger, on the job, had non-constipation, shorter duration of hypertension, taking antihypertensive drugs and higher BP levels, which is consistent with the study by Xu et al. [16]. The average BMI of participants ranged from 25.3 ± 4.8 to 26.3 ± 4.6 kg/m^2^, indicating that most participants were overweight, which may be related to non-exercise or low exercise intensity. Up to 27% of the patients maintained daily exercise in this study. The blood pressure control level of the depression or anxiety group was higher than that of the non-depression or anxiety group. This may be due to the fact that it is more difficult to control blood pressure when the patient has hypertension combined with depression [29].

### 4.1. The Status of DF Intake

In recent years, there is evidence that insufficient intake of vegetables and fruits, and consumption of more fine processed food lead to insufficient intake of DF in China [30]. In this group, the median daily intake of DF of total participants was 10.4 g, which is consistent with the study by Jindan et al. [31], who found that the average daily DF intake was (10.17 ± 4.38) g in hypertensive patients. Only five participants (1.1%) had enough DF intake that reached the quantity of the recommended Dietary Reference Intake (25–30 g/d) [32]. These results indicated that most hypertensive patients may not have sufficient dietary fiber intake in China.

### 4.2. The Status of Depression and Anxiety

Poor blood pressure control, drug side effects and economic burden act as stressors on the brain, causing the patient to produce negative emotions (depression and anxiety) [33,34]. The present study found that the prevalence of hypertension combined with depression, anxiety, depression and anxiety comorbidities were 19.6%, 18.5%, and 10.7%, respectively, which were similar to the results by Li et al. [4]. However, it is lower than the results of Yang [6], which showed that the prevalence of hypertension combined with depression, anxiety, depression and anxiety comorbidities were as high as 47.9% and 63%, 36.4%, respectively. The main reason for the differences may be related to the exclusion of participants with serious complications and comorbidities in this study. In the study of Yang FF, 63 (50%) participants had heart disease and 30 (23.8%) participants had diabetes. Patients with hypertension combined with cervical cerebrovascular disease and diabetes have a higher prevalence of anxiety and depression than hypertension patients [35].

### 4.3. The Association between DF Intake and Depression in Patients with Hypertension

Intestinal microbes and the gut–brain axis share some common pathogenesis in the occurrences of anxiety and depression [36,37,38]. Dietary fiber may improve negative emotions by regulating the intestinal flora [21,39], thereby improving the intestinal–brain axis in patients with hypertension.

This study showed that DF intake is associated with depression, that is an increased DF intake is associated with a decreased prevalence of depression in hypertensive patients, which is consistent with the results of Xu and Miki’s studies [16,17]. Compared with the hypertensive patients with a DF intake ≥15.4 g/d, the incidence of depression in the patients with a DF 10.5 to <15.4 g/d increased by 2.641 times. At present, there is no reported study about the relationship between DF intake and negative emotions in hypertensive patients. However, Xu et al. surveyed 40,617 general populations in the United States and found that people with DF intake more than 14.9 g/d had a 42% lower risk of depression than those with less than 10.5 g/d [16]. While for the groups with DF intake <8.1 g/d and 8.1 g/d < DF < 10.5 g/d, compared with the group with DF intake ≥15.4 g/d, there were no statistically significant increases. One of the possible reasons is that energy intake is associated with the prevalence of depression [40,41]. In this study, we found that energy intake was a risk factor for depression (*p* < 0.05). The potential increased risk of depression due to low dietary fiber intake may be compensated for by the benefits of reduced energy intake in Q1 (1758 Kcal) and Q2 (1985 Kcal), compared with Q4 with an energy of 2239 Kcal).

### 4.4. The Association between DF Intake and Anxiety in Patients with Hypertension

Some studies have shown that high DF intake can relieve anxiety in the general population [11]. DF may regulate anxiety through the regulation of inflammatory factors and neurotransmitter levels by acetic acid [42,43]. In this study of patients with hypertension, DF intake is associated with the score of anxiety and an increased intake of DF is associated with a decrease in the prevalence of anxiety. After adjusting for the demographic, clinical and nutritional indicators, DF still is associated with the prevalence of anxiety. Compared with patients with the intake of DF ≥15.4 g/d, the incidences of anxiety increased in the patients with daily DF intake <8.1 g by 2.757 times. These results are consistent with the results of the study of Andrew and Hepsomali et al. in the general population [11,13]. Hepsomali et al. also found that the high, medium/high, and medium fiber intake groups had a lower number of mental health symptomatology compared to the low/medium and low fiber intake groups [13]. In addition, the energy did not affect the level of anxiety in the Q1 and Q2 groups but did in Q3. The underlying mechanism is not clear. Until now, no other studies have reported the relationship between DF intake and anxiety in patients only with hypertension.

DF primarily includes β-glucan and oligosaccharides (such as galactose oligosaccharides), lignin, inulin, etc. [42]. β-glucan mainly produces butyric acid, a kind of SCFA [42]. If the decreased butyric acid increases intestinal permeability and damages the intestinal barrier [44], thus inflammatory factors can enter the circulatory system [45], while inflammation is closely related to the onset of depression [46]. However, galactose oligosaccharides mainly produce acetic acid [42], which not only reduces the expression of inflammatory factors such as IL-1β, IL-6 and tumor necrosis factor α [43], it can also increase the neurotransmitter such as glutamate, gamma-aminobutyric acid levels in the hypothalamus which, as found before, can relieve anxiety [47].

Since this study did not classify the DF of participants’ intake, we could not understand the kinds of DF intake. Some patients may mainly intake β-glucan, while other people may consume more galactose oligosaccharides, which leads to the different associations between dietary fiber and anxiety/depression in this population.

## 5. Conclusions

Higher consumption of DF was a protective factor for depression and anxiety in hypertensive patients. The increased risk of depression due to low DF intake may be compensated by the benefits of reduced energy intake in hypertensive patients.

## 6. Strengths and Limitations

In this study, the associations between DF and depression and anxiety in patients with hypertension were examined after adjusting for a variable of total energy intake. However, there were some limitations in this study. Firstly, since this was a cross-sectional investigation, we only made a relationship between dietary fiber intake and psychological comorbidities in patients with hypertension. Cohort studies or prospective intervention studies are needed to verify this relationship in the future. Secondly, using a single question to evaluate sleep quality is a limitation, which might lead to no differences between patients with anxiety/depression and those without anxiety/depression. Thirdly, since there were very few participants who had enough DF intake, it is impossible to obtain the risk coefficient multiples of incidence of negative emotions in the insufficient DF intake group compared with the group of adequate DF intake which will be offset by conducting prospective interventional studies. Fourthly, we did not explore the relationship between intake of dietary fiber from different sources and psychological comorbidities, which could be a subject for future research projects. Due to limited funds, we did not measure the mechanism of DF in improving psychological comorbidities in patients with hypertension. Lastly, the amount of DFI was roughly estimated based on the retrospective dietary diary which may cause potential measurement error.

## Figures and Tables

**Table 1:** Socio-demographic and clinical characteristics (*n* = 459).

Characteristics		Depression ($\bar{x}$ ± s/*n* (%)/M (P25, P75)				Anxiety ($\bar{x}$ ± s/*n* (%)/M (P25, P75)			
		Yes (N = 90)	No (N = 369)	t/χ^2^/z	*p*	Yes (N = 85)	No (N = 374)	t/χ^2^/z	*p*
Age (y)		46.2 ± 11.9	52.9 ± 12.5	4.382 ^a^	<0.001 ***	46.1 ± 12.9	52.5 ± 12.3	4.256 ^a^	<0.001 ***
Sex	Male	56 (62.2)	232 (62.9)	0.013 ^b^	0.909	53 (62.4)	235 (62.8)	0.007 ^b^	0.934
	Female	34 (37.8)	137 (37.1)			32 (37.6)	139 (37.2)		
Marital status	Single	0 (0.0)	18 (4.9)	5.636 ^b^	0.131	0 (0.0)	18 (4.8)	5.252 ^b^	0.154
	Married	90 (100.0)	347 (94.0)			85 (100.0)	352 (94.1)		
	others	0 (0.0)	4 (1.1)			0 (0.0)	4 (1.1)		
Occupational status	On the job	70 (77.8)	216 (58.5)	27.993 ^b^	<0.001 ***	64 (75.3)	222 (59.4)	12.051 ^b^	0.002 **
	Retired	20 (22.2)	153 (41.5)			21 (24.7)	152 (40.6)		
Regular exercise	Yes	22 (24.4)	98 (26.6)	0.167 ^b^	0.682	19 (21.2)	101 (27.0)	0.776 ^b^	0.378
	No	68 (75.6)	271 (73.4)			66 (77.6)	273 (73.0)		
Duration of sleep (h)		6.86 ± 1.07	6.84 ± 1.14	−0.184 ^a^	0.854	7.0 (6.5, 7.5)	7.0 (6.0, 7.5)	−1.079 ^d^	0.281
Quality of sleep	Poor	10 (11.1)	27 (7.3)	3.913 ^b^	0.141	10 (11.8)	27 (7.2)	2.083 ^b^	0.353
	Fair	80 (88.9)	342 (92.7)			75 (88.2)	347 (92.8)		
Smoking status	Yes	25 (27.8)	93 (25.2)	0.251 ^b^	0.616	25 (29.4)	93 (24.9)	0.749 ^a^	0.387
	No	85 (94.4)	276 (74.8)			60 (70.6)	281 (75.1)		
Alcohol drinking	Yes	24 (26.7)	86 (23.3)	0.448 ^b^	0.503	19 (22.4)	91 (24.3)	0.149 ^a^	0.700
	No	66 (73.3)	283 (76.7)			66 (77.6)	283 (75.7)		
BMI (kg/m^2^)		26.25 ± 4.59	25.25 ± 4.78	−1.794 ^a^	0.073	25.93 ± 5.22	25.34 ± 4.64	−1.032 ^a^	0.303
Constipation	Yes	14 (15.6)	29 (7.9)	5.048 ^b^	0.025 *	10 (11.8)	33 (8.8)	0.706 ^b^	0.401
	No	76 (84.4)	340 (92.1)			75 (88.2)	341 (91.2)		
Duration of HTN (y)		1.0 (0.5, 6.3)	5.0 (1.0, 10.0)	−3.427 ^d^	0.001 **	1.0 (0.5, 5.5)	5.0 (1.0, 5.0)	−3.514 ^d^	<0.001 ***
Taking drugs	Yes	52 (57.8)	303 (82.1)	24.452 ^b^	<0.001 ***	48 (56.5)	307 (82.1)	25.932 ^b^	<0.001 ***
	No	38 (42.2)	66 (17.9)			37 (43.5)	48 (12.8)		
SBP (mmHg)		143.5 ± 15.3	134.6 ± 12.4	−5.789 ^a^	<0.001 ***	140.0 (133.0, 147.5)	135.0 (126.0, 143.0)	−3.618 ^d^	<0.001 ***
DBP (mmHg)		92.6 ± 12.4	84.8 ± 10.4	−6.155 ^a^	<0.001 ***	89.8 ± 10.7	85.5 ± 11.2	−3.261 ^a^	0.001 **
Complication	Yes	3 (3.3)	2 (0.5)	5.232 ^c^	0.054	1 (1.2)	4 (1.1)	0.007 ^c^	0.932
	No	87 (96.7)	367 (99.5)			84 (98.8)	370 (98.9)		
Comorbidity	Yes	10 (11.1)	52 (14.1)	0.550 ^b^	0.458	8 (9.4)	54 (14.4)	1.498 ^b^	0.221
	No	80 (88.9)	317 (85.9)			77 (90.6)	320 (85.6)		
Energy		1849.4 ± 344.2	2035.9 ± 366.9	4.375	<0.001 ***	1863.8 ± 366.6	2030.1 ± 363.9	3.799	<0.001 ***

Note: ***: *p* < 0.001; **: *p* < 0.01; *: *p* < 0.05. HTN: hypertension; Taking drugs means taking antihypertensive drugs; BMI, body mass index; DBP, Diastolic blood pressure; SBP, Systolic blood pressure. ^a^ Independent-samples T test; ^b^ Pearson Chi-square; ^c^ Yates’ correction Chi-square; ^d^ Mann–Whitney U; M (P25, P75), median (25th and 75th percentiles).

**Table 2:** The status of DF intake.

Dietary Fiber Intake (g/d)	N (%)	DF Intake (g/d), M (P_25_, P_75_)
Total fiber	459 (100.00)	10.4 (8.0, 13.3)
Q1 (<8.1)	121 (26.4)	6.3 (5.0, 7.3)
Q2 (8.1 to <10.5)	114 (24.8)	9.4 (8.9, 10.1)
Q3 (10.5 to <15.4)	118 (25.7)	12.0 (11.1, 12.6)
Q4 (≥15.4)	106 (23.1)	17.8 (14.9, 20.8)

Note: M (P_25_, P_75_), median (25th and 75th percentile); Q1, P_0^−^_ < P_25_; Q2, P_25^−^_ < P_50_; Q3, P_50^−^_ P_75_; Q4, P_75^−^_ P_100_.

**Table 3:** Regression analysis summary for the score of depression.

Variables	Crude			Model 1			Model 2			Model 3		
	B (95% CI)	t	*p*	B(95% CI)	t	*p*	B(95% CI)	t	*p*	B (95% CI)	t	*p*
DFI (g)	0.609 (−0.796, −0.422)	−6.425	<0.001	−0.604 (−0.788, −0.420)	−6.473	<0.001	−0.533 (−0.720, −0.346)	−5.615	<0.001	−0.346 (−0.547, −0.145)	−3.393	0.001
Age	-	-	-	−0.126 (−0.206, −0.045)	−3.086	0.002	−0.106 (−0.720, −0.346)	−2.532	0.012	−0.103 (−0.183, −0.023)	−2.551	0.001
SBP	-	-	-	-	-	-	0.104 (0.019, 0.189)	2.410	0.017	0.111 (0.029, 0.193)	2.665	0.008
Energy	-	-	-	-	-	-	-	-	-	−0.006 (−0.009, −0.003)	−4.139	<0.001

Model 1 Adjusted for age, occupational status. Model 2 Adjusted for variables in model 1 + constipation, duration of hypertension, taking antihypertensive drugs, systolic blood pressure (SBP), diastolic blood pressure (DBP). Model 3 Adjusted for variables in model 1 + model 2 + energy intake.

**Table 4:** Odds ratios and 95% confidence intervals for depression according to dietary fiber intake.

Variables	Cases		Crude	Model 1	Model 2	Model 3
	Yes	No	OR (95% CI)	OR (95% CI)	OR (95% CI)	OR (95% CI)
Q4	9	97	1.00 (Ref.)	1.00 (Ref.)	1.00 (Ref.)	1.00 (Ref.)
Q1	27	94	3.096 (1.383, 6.931) **	3.641 (1.579, 8.414) **	3.796 (1.578, 9.136) **	2.042 (0.745, 5.600)
Age	-	-	-	0.966 (0.935, 0.999) *	-	-
Energy	-	-	-	-	-	0.999 (0.997, 1.000) *
Q2	23	91	2.724 (1.197, 6.197) *	2.988 (1.282, 6.965) *	3.287 (1.236, 8.742) *	2.036 (0.709, 5.843)
Age	-	-	-	0.953 (0.923, 0.985) **	-	-
Occupational status	-	-	-	-	-	2.674 (1.058, 6.759) *
Antihypertensive drugs	-	-	-	-	0.159 (0.061, 0.413) ***	0.153 (0.055, 0.428) ***
DBP	-	-	-	-	-	1.080 (1.011, 1.1531) *
Energy	-	-	-	-	-	0.997 (0.995, 0.999) ***
Q3	37	81	3.840 (1.732, 8.518) **	4.240 (1.861, 9.662) **	3.100 (1.275, 7.538) *	2.641 (1.050, 6.640) *
Age	-	-	-	0.957 (0.926–0.990) *	-	--
SBP	-	-	-	-	1.058 (1.014–1.105) *	1.058 (1.013–1.106) *
Energy	-	-	-	-	-	0.998 (0.007–0.999) **

Notes: ***: *p* < 0.001; **: *p* < 0.01; *: *p* < 0.05. Model 1 Adjusted for age, occupational status. Model 2 Adjusted for variables in model 1 + constipation, duration of hypertension, taking antihypertensive drugs, systolic blood pressure (SBP), diastolic blood pressure (DBP). Model 3 Adjusted for variables in model 1 + model 2 + energy intake.

**Table 5:** Regression analysis summary for the score of anxiety.

Variables	Crude			Model 1			Model 2			Model 3		
	B (95% CI)	t	*p*	B(95% CI)	t	*p*	B(95% CI)	t	*p*	B (95% CI)	t	*p*
DFI (g)	−0.630 (−0.807, −0.453)	−7.032	<0.001	−0.626 (−0.801, −0.450)	−7.022	<0.001	−0.591 (−0.772, −0.410)	−6.4421	<0.001	−0.565 (−0.768, −0.363)	−5.502	<0.001
Age	-	-	-	−0.082 (−0.158, 3.024)	−2.098	0.037	-	-	-	-	-	-

Model 1 Adjusted for age, occupational status. Model 2 Adjusted for variables in model 1 + duration of hypertension, taking antihypertensive drugs, systolic blood pressure (SBP), diastolic blood pressure (DBP). Model 3 Adjusted for variables in model 1 + model 2 + energy intake.

**Table 6:** Odds ratios and 95% confidence intervals for anxiety according to dietary fiber intake.

Variables	Cases		Crude	Model 1	Model 2	Model 3
	Yes	No	OR (95% CI)	OR (95% CI)	OR (95% CI)	OR (95% CI)
Q4	11	95	1.00 (Ref.)	1.00 (Ref.)	1.00 (Ref.)	1.00 (Ref.)
Q1	31	90	2.975 (1.411, 6.271) **	0.619 (0.471, 0.812) **	3.469 (1.458, 8.250) **	2.757 (1.035, 7.346) *
Age	-	-	-	0.940 (0.909–0.971) ***	0.953 (0.918, 0.990) **	0.953 (0.917, 0.989) *
Q2	21	93	1.950 (0.891, 4.269)	2.178 (0.960, 4.941)	1.940 (0.782, 4.813)	1.638 (0.641, 4.190)
Age	-	-	-	0.947 (0.916, 0.979) **	0.957 (0.923, 0.991)	0.960 (0.925, 0.996) *
Antihypertensive drugs	-	-	-	-	0.340 (0.138–0.835) *	0.354 (0.143–0.878) *
Q3	22	96	1.979 (0.910–4.306)	0.481 (0.217–1.065)	0.558 (0.244–1.277)	1.488 (0.639–3.464)
Age	-	-	-	0.957 (0.925–0.990) *	0.961 (0.927–0.997) *	-
Energy	-	-	-	-	-	0.999 (0.997–1.000) *

Notes: ***: *p* < 0.001; **: *p* < 0.01; *: *p* < 0.05. Model 1 Adjusted for age, occupational status. Model 2 Adjusted for variables in model 1 + duration of hypertension, taking antihypertensive drugs, systolic blood pressure (SBP), diastolic blood pressure (DBP). Model 3 Adjusted for variables in model 1 + model 2 + energy intake.

## Data Availability

The data in this study involves privacy issues, data should not be shared.

## Supplementary Materials

The following are available online at https://www.mdpi.com/article/10.3390/nu13114159/s1. “2018 Chinese Guidelines for Prevention and Treatment of Hypertensionn—A report of the Revision Committee of Chinese Guidelines for Prevention and Treatment of Hypertension” Supplementary Table S1: Socio-demographic and clinical characteristics in the hospital (*n* = 322); Supplementary Table S2: Socio-demographic and clinical characteristics in the community (*n* = 137).
