# Supplementary material for: Association between Dietary Fiber Intake and Incidence of Depression and Anxiety in Patients with Essential Hypertension
Source: Nutrients. 2021 Nov 20;13(11):4159. doi: 10.3390/nu13114159 (PMC8623764; doi:10.3390/nu13114159)
Supplement: Supplementary file 1 [file nutrients-13-04159-s001.zip › nutrients-1375234-supplementary.pdf]

Journal of Geriatric Cardiology (2019) 16: 182–241
©2019 JGC All rights reserved; www.jgc301.com

Guidelines **· Open Access ·**

2018 Chinese Guidelines for Prevention and Treatment of Hypertension—A report of the Revision Committee of Chinese Guidelines for Prevention and Treatment of Hypertension

Joint Committee for Guideline Revision

Chinese Hypertension League
Chinese Society of Cardiology
Hypertension Committee of the Chinese Medical Doctor Association
Hypertension Branch of the China Association for the Promotion of International Exchanges of Health Care
Hypertension Branch of the Chinese Geriatrics Society

Revision Committee Members

Chair: Li–Sheng LIU*
Deputy Chairs: Zhao–Su WU, Ji–Guang WANG, Wen WANG
Members (Listed in alphabetic order by last name in Chinese Pinyin)
Yu–Jing BAO, Jun CAI, Lu–Yuan CHEN, Wei–Wei CHEN, Shao–Li CHU, Ying–Qing FENG, Ping–Jin GAO, Ting–Rui GUAN, Zi–Hong GUO, Qi HUA, Jun HUANG, Yong HUO, Wei–Ping JIA, Xiong–Jing JIANG, Yi–Nong JIANG, Li LI, Li–Huan LI, Nan–Fang LI, Wei LI, Xiao–Ying LI, Xue–Wang LI, Yan LI, Yong LI, Jin–Xiu LIN, Li–Sheng LIU, Xin–Zheng LU, Ji–Xiang MA, Jie MI, Jian-Jun MU, Chang–Yu PAN, Gang SUN, Ning–Ling SUN, Ying–Xian SUN, Jun TAO, Hao WANG, Ji–Guang WANG, Wen WANG, Wen–Zhi WANG, Yong–Jun WANG, Yu WANG, Zeng–Wu WANG, Hai–Ying WU, Zhao–Su WU, Liang–Di XIE, Zhang–Rong XU, Xiao–Wei YAN, Yan–Min YANG, Chong–Hua YAO, Zheng–Pei ZENG, Jian ZHANG, Wei–Zhong ZHANG, Xin–Hua ZHANG, Xin–Jun ZHANG, Yu–Qing ZHANG, Ding–Liang ZHU, Jun ZHU, Zhi–Ming ZHU

Scientific Committee Members

Xiang–Mei CHEN, Hong CHEN, Jun–Shi CHEN, Xiao–Ping CHEN, Yun–Dai CHEN, Zhao–Qiang CUI, Xue–Ping DU, Li FAN, Run–Lin GAO, Jun–Bo GE, Luo–Bu GE–SANG, Dong–Feng GU, Jing–Xuan GUO, Yi–Fang GUO, Qing–Hua HAN, Ya–Ling HAN, Zhao–Guang HONG, Da–Yi HU, Jian–Ping LI, Guang–Wei LI, Guang–Ping LI, Xin–Li LI, Yu–Ming LI, Xiao–Feng LIANG, Yi–Shi LI, Yu–Hua LIAO, Shu–Guang LIN, Mei–Lin LIU, Jing LIU, Wei LIU, Fang–Hong LU, Shu–Ping MA, Qian–Hui SHANG, Yi–Pu CHEN, Hai–Qin Tang, Xin–Hua TANG, Gang TIAN, Ke–An WANG, Xing–Yu WANG, Dao–Wen WANG, Shou–Ling WU, Cheng–Bin XU, Shou–Chun XU, Xin–Juan XU, Xin–Hua YIN, Bo YU, Guo–Ying YU, Jing YU, Wei YU, Meng–Sun YU, Hong YUAN, Ru–Yu YUAN, Chun–Yu ZENG, Zhe–Chun ZENG, Lin ZHANG, Hui–Min ZHANG, Dong ZHAO, Luo–Sha ZHAO, Lian–You ZHAO, Xiao–Fang ZHOU, Man–Lu ZHU

J Geriatr Cardiol 2019; 16: 182–241. doi:10.11909/j.issn.1671-5411.2019.03.014

Table of Contents

Preamble
1 Prevalence of hypertension in Chinese population
1.1 Prevalence, incidence and epidemic trend of hypertension in Chinese population
1.2 Awareness, treatment and control of Hypertension in China
1.3 Important risk factors for hypertension in Chinese population
2 Hypertension and cardiovascular risk
2.1 Relationship between blood pressure and cardiovascular risk
2.2 Cardiovascular risk characteristics of hypertension population in China
3 Diagnostic evaluation
3.1 Medical history

***Correspondence to:** llschl@126.com (LIU LS)

3.2 Physical examination
3.3 Laboratory examinations
3.4 Genetic analysis
3.5 BP measurement
3.6 Evaluation of target organ damage
4 Classification and stratification of hypertension
4.1 Classification by BP level
4.2 Cardiovascular risk stratification
5 Treatment of hypertension
5.1 Goals of treatment
5.2 Therapeutic strategies
5.3 Lifestyle intervention
5.4 Pharmacological therapy of hypertension
5.5 Advance in device-based hypertension treatment
5.6 Treatment of associated risk factors
5.7 Follow-up, referral and medical record of anti-hypertensive therpay
6 Treatment of hypertension for special populations
6.1 Hypertension in the elderly
6.2 Hypertension in children and adolescents
6.3 Hypertensive disorders in pregnancy
6.4 Hypertension with stroke
6.5 Hypertension with coronary arteary disease
6.6 Hypertension with heart failure
6.7 Hypertension in chronic kidney disease
6.8 Concomitant hypertension and dlabetes mellitus
6.9 Metabolic syndrome
6.10 Antihypertensive treatment for peripheral arterial disease
6.11 Refractory hypertension
6.12 Hypertensive emergencies and hypertensive urgencies
6.13 Perioperative management of hypertension
7 Hypertension prevention and treatment measures and strategies
7.1 Prevention and treatment policy and health service system
7.2 Community hypertension prevention and treatment strategy
8 Community-based standardized management of hypertension
8.1 Community-based standardized management of hypertension
8.2 Management of first-time visit hypertensive patients
8.3 Hierarchical long-term follow-up for hypertension management
8.4 Health education for hypertensive patients
8.5 Remote management of hypertensive patients
8.6 Team building
8.7 Hierarchical medical system of hypertensive patients
8.8 Self-management of hypertensive patients
9 Secondary hypertension
9.1 Renal parenchymal hypertension
9.2 Hypertension caused by renal artery stenosis and other vascular diseases
9.3 Obstructive sleep apnea syndrome
9.4 Primary aldosteronism and other endocrine hypertension
9.5 Other rare types of secondary hypertension
9.6 Drugs induced hypertension
9.7 Monogenic Inherited Hypertension
10 Research prospect
References
Schedule

Preamble

Since 2010, as research evidence is ever-increasing regarding hypertension and related diseases worldwide, guidelines for hypertension have been successively developed or revised in many countries and regions. In China, new evidence from population studies and clinical trials are accumulating, including representative sampling survey on the population with hypertension in the 12th Five-Year Plan, FEVER study subgroup, Chinese Hypertension Intervention Efficacy study (CHIEF), China Stroke Primary Prevention Trial (CSPPT). These studies have provided further information and evidence for the revision of Chinese guidelines for hypertension.

In September 2015, supported by the former Disease Control Bureau of National Health and Family Planning Commission of China, the Chinese Hypertension League, in partnership with Chinese Society of Cardiovascular Disease of the Chinese Medical Association, Hypertension Branch of the China International Exchange and Promotion Association for Medical and Healthcare, Hypertension Branch of the Chinese Geriatrics Society, and the Hypertension Committee of Chinese Medical Doctor Association organized a committee for revising the *2010 Chinese Guidelines for the Management of Hypertension*. Over the past two years, discussions have been conducted for many times with respect to guiding ideas and plans of guideline revision. In the survey on major problems for revision of the guideline, 20 issues have been proposed including: goal of blood pressure (BP) control for treatment of hypertension, management of hypertension among special populations, role of β-blocker in treatment of hypertension. In response to these survey results, document retrieval has been extensively performed. Document database include China Biology Medicine (CBM), Wanfang Data

Knowledge Service Platform, CNKI Platform, PubMed, Excerpta Medica Database (EMBASE), and platform of Institute of Medical Information & Library, CMAS&PUMC. After writing the first draft of the revised guideline, nearly 30 symposiums had been held by the committee for revision of guidelines, in which in-depth discussions were made regarding problems and development trends of management of hypertension. For those controversial issues, the consensuses were made with anonymous electronic voting by committee members. In early 2018, the newly revised guideline was reviewed and revised by a special group consisting 10 members of the committee, including five cardiologists, two epidemiologists, one endocrinologist, and one nephrologist. Subsequently, "the 2018 exposure draft for Chinese guidelines for the management of hypertension" was released to collect peers comments, based on which the final draft was completed.

By referring to the guideline development processes of World Health Organization (WHO) and Chinese Medical Association (CMA), assessment had been performed regarding important content, level of evidence (Table 1), and class of recommendation in this revised Guidelines (Table 2), with considerations on recent scientific developments on related fields, and current reality of hypertension management in China, the 2018 guidelines provide guidance for prevention and intervention, diagnosis and evaluation, classification and stratification and treatment and management of hypertension.

Revision Committee of 2018 Chinese Guidelines for Hypertension

Table 1. Class of recommendation.

	Definition	Recommended expression
Class I	Evidence and/or overall agreement that the treatment or method is beneficial, useful or effective	Recommended/indication
Class II	Inconsistent evidence and/or disagreement about the use/effect of the treatment or method	
Class IIa	Evidence/views tend to be useful/effective	Should be considered
Class IIb	Evidence/views are not sufficient to establish useful/effective	Can consider it
Class III	Evidence and/or expert agree that the treatment or method is useless/ineffective and may be harmful in some cases	Not recommended

Table 2. Level of evidence.

Level A	Data from multiple randomized controlled clinical trials or meta-analyses consisting of randomized controlled clinical trials
Level B	Data from a single randomized clinical trial or multiple large randomized controlled trials
Level C	Data from expert consensus and/or small-scale studies, retrospective studies or registration studies

1. Prevalence of hypertension in Chinese population

Key point 1. Prevalence of hypertension in Chinese population

- The prevalence of hypertension is still increasing in China. There are two significant features of the population with hypertension in China: the prevalence of hypertension increases from the south to the north; the prevalence of hypertension varied among different ethnic groups.
- The awareness, treatment and control (crude rate) of hypertension patients in China have increased significantly in recent years, reaching 51.6%, 45.8% and 16.8% respectively, but is overall at a low level.
- The important risk factors for hypertension are high sodium intake, low potassium diet, overweight, and obesity in Chinese population.

1.1 Prevalence, incidence and epidemic trend of hypertension in Chinese population

The 2012–2015 national hypertension survey,[1] showed the overall crude prevalence of hypertension was 27.9% (weighted rate 23.2%) in Chinese residents aged 18 and over. Compared with the previous national surveys of hypertension conducted in 1958–1959, 1979–1980, 1991, 2002 and 2012,[2] the trend keeps on increasing, although the total participants, age and diagnostic criteria for each survey are not completely consistent (Table 3).

The prevalence of hypertension increases significantly with age. According to the 2012–2015 national survey, the prevalence of hypertension in young people aged 18 to 24, 25 to 34, and 35 to 44 years old was 4.0%, 6.1%, and 15.0%, respectively.[1] It is higher in male than in female. The prevalence of hypertension in the north is still higher than that in the south. The pattern is gradually changing into a higher prevalence of hypertension in large and medium-sized cities, such as in Beijing (35.9%), Tianjin (34.5%) and Shanghai (29.1%),[1] respectively. The prevalence of hypertension in rural areas increased faster than that in urban areas. According to the national survey from 2012 to 2015, the prevalence rate in rural areas (crude rate 28.8%, standardized rate 23.4%) surpassed that of in urban areas (crude rate: 26.9%, standardized rate: 23.1%) for the first time. Compared with different ethnic groups, the prevalence of

Table 3. Results of six prevalence rates surveys of hypertension in China.

Year	No. of surveyed province/ city/autonomous region	Age, yrs	Diagnostic criteria	No. of participants	No. of hypertension	Prevalence, %
1958–1959	13	≥ 15	Disunity	739,204	37,773	5.1^{Δ}
1979–1980	29	≥ 15	Diagnosis of hypertension: ≥ 160/95 mmHg Critical hypertension: 140–159/90–95 mmHg	4,012,128	310,202	7.7^{Δ}
1991	29	≥ 15	≥ 140/90 mmHg and (or) taking antihypertensive drugs within two weeks	950,356	129,039	13.6^{Δ}
2002	29	≥ 18	≥ 140/90 mmHg and (or) taking antihypertensive drugs within two weeks	272,023	51,140	18.8^{Δ}
2012	31	≥ 18	≥ 140/90 mmHg and (or) taking antihypertensive drugs within two weeks	-	-	25.2¶
2015	31	≥ 18	≥ 140/90 mmHg and (or) taking antihypertensive drugs within two weeks	451,755	125,988	27.9^{Δ}

$^{\Delta}$Rough rate; ¶Comprehensive adjustment of prevalence.

hypertension among Tibetans, Manchus and Mongolians is higher than that of Han, but is lower in Hui, Miao, Zhuang and Buyi.[3]

The research on the incidence of hypertension is relatively insufficient. One study with 10,525 non-hypertensive patients aged over 40 years of age showed: the cumulative incidence of hypertension in males and females was 28.9% and 26.9%, respectively, after follow up for an average of 8.2 years in 1991–2000,[4] the incidence increased with age (Figure 1).

1.2 Awareness, treatment and control of hypertension in China

Awareness, treatment and control of hypertension are important parameters for reflecting status of the treatment and prevention of hypertension. According to the 2015 survey, the awareness, treatment, and control of hypertension in people over 18 years of age were 51.6%, 45.8%, and 16.8%, respectively, which were significantly higher than those in 1991 and 2002 (Table 4).[1] The prospective study of chronic diseases in China[5] from 2004 to 2009 showed that the rate of hypertension control was lower than that of 2002, which may be related to the sample method.

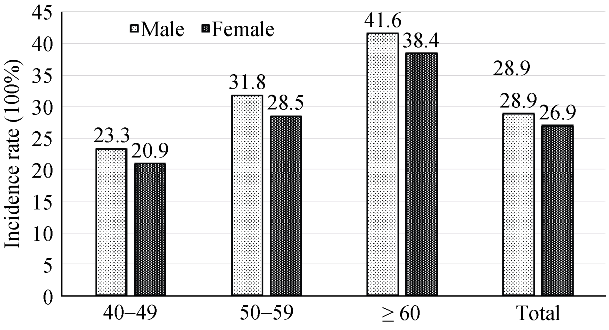

Figure 1. Cumulative incidence of hypertension in different genders and baseline age groups from 1991 to 2000

Table 4. Survey results of awareness, treatment and control (roughness rate) of hypertension in China.

Year	Age, yrs	Awareness, %	Treatment, %	Control, %
1991	≥ 15	26.3	12.1	2.8
2002	≥ 18	30.2	24.7	6.1
2012	≥ 18	46.5	41.1	13.8
2015	≥ 18	51.5	46.1	16.9

The awareness, treatment and control rate were higher in female than in male, and the treatment was significantly higher in urban than that in rural areas.[6] At the same time, these rates of residents in the southern region are higher than the north.[7,8] With respect to ethnic groups, the treatment and control of hypertension is lower among ethnic minority than that of Han nationality.[1,9]

1.3 Important risk factors for hypertension in Chinese population

Risk factors for hypertension include genetic factors, age, and unhealthy lifestyles. The clustering of risk factors is prevalent in the population. As the number and severity of hypertension risk factors increase, BP levels increase, and the risk of hypertension increases.[10–13]

1.3.1 High sodium, low potassium diet

High sodium and low potassium diets are important risk factors for hypertension in Chinese population. The INTERSALT study found that the median 24-h urinary sodium excretion increased by 2.3 g (100 mmol/day), and the median systolic blood pressure (SBP)/diastolic blood pressure (DBP) increased by an average of 5–7/2–4 mmHg. A survey found that the average cooking salt intake of residents aged 18 and over in China was 10.5 g in 2012, although it was lower than 12.9 g in 1992 and 12.0 g in 2002, but was still 75.0% higher than the recommended salt intake

level,[15] while the Chinese population was generally sensitive to sodium.[14,16]

1.3.2 Overweight and obesity

Overweight and obesity significantly increase the risk of all-cause mortality in the global population[17] and are also important risk factors for hypertension. For people aged 35–64, the overweight rate is 38.8%, and the obesity rate is 20.2%, which are higher in female than in male, in urban than in rural areas, and in north than south.[18] A follow-up study[19] on the relationship between overweight/obesity and hypertension in Chinese adults found that the risk of hypertension was 1.16 to 1.28 times higher in the overweight and obesity groups than in the normal weight group along with the increase of body mass index (BMI).[1]

Visceral obesity is closely related to hypertension. With the increase of visceral fat index, the risk of hypertension increases.[20] In addition, visceral obesity is closely related to metabolic syndrome, which can lead to abnormal glucose and lipid metabolism.[21]

1.3.3 Excessive drinking

Excessive drinking includes dangerous drinking (male: 41–60 g, female 21–40 g) and harmful drinking (more than 60 g for men and 40 g for women). Drinking is prevalent in China. The harmful drinking rate is 9.3%[15] among residents over 18 years old. Restricted drinking was significantly correlated with a drop in BP, with an average reduction in alcohol intake of 67%, a decrease in SBP of 3.31 mmHg and in DBP of 2.04 mmHg.[22] At present, there is insufficient evidence supports the correlation between cardiovascular health and less alcohol intake. However, some relevant research shows that even for people who drink a small amount of alcohol, reducing alcohol intake can improve cardiovascular health and reduce the risk of cardiovascular disease.[23]

1.3.4 Long-term nervousness

Long-term nervousness is a risk factor for hypertension, and mental stress can activate sympathetic nerves and increase BP.[24,25] A meta-analysis of 13 cross-sectional studies and eight prospective studies[26] showed that the risk of hypertension was 1.18 times (95% CI: 1.02–1.37) and 1.55 times (95% CI: 1.24–1.94) among people with mental stress (defined as anxiety, stress, anger, panic or fear) than the normal population.

1.3.5 Other risk factors

In addition to the above risk factors for hypertension, other risk factors include age, family history of hypertension, lack of physical activity, and diabetes, *etc*. Studies have shown that exposure to $PM_{2.5}$, PM_{10}, SO_2, and O_3 is associated with increased risk of hypertension and increased mortality from cardiovascular disease.[27,28]

2 Hypertension and cardiovascular risk

Key point 2. Hypertension and cardiovascular risk

- BP is continuously, independently, directly and positively associated with cardiovascular risk.
- Stroke is still the most important complication of hypertension in China by now, while the incidence of coronary events also increased evidently. Other complications include heart failure, left ventricular hypertrophy, atrial fibrillation and end-stage renal disease.

2.1 Relationship between BP and cardiovascular risk

There is a close causal relationship between BP and cardiovascular morbidity and mortality. A global prospective observational study of 61 populations (about 1 million people aged 40–89 years with the baseline BP ranging from 115/75 mmHg to 185/115 mmHg and with an average follow-up of 12 years)[29] showed that clinic SBP or DBP was continuously, independently, directly and positively associated with the risk of stroke, coronary heart disease (CHD) events and cardiovascular mortality. Each 20 mmHg increase in SBP or 10 mmHg increase in DBP was associated with doubled risk of cardiovascular and cerebrovascular morbidity.

In the Asia Pacific cohort study (APCSC) which consists of 13 Chinese populations,[30] clinic BP was closely associated with the risk of stroke and ischemic heart disease events, and the association between elevated BP and stroke or ischemic heart disease events in Asian populations was stronger than in Australia and New Zealand populations. For each 10 mmHg increase in SBP, the risk of stroke and fatal myocardial infarction increased by 53% and 31% respectively in Asian populations, while by 24% and 21% respectively in Australian and New Zealand populations.

There is also a causal relationship between BP level and heart failure. Clinical follow-up data showed that the incidence of heart failure increased with BP level.[31] Heart failure and stroke were the two complications most closely related to BP level. Chronic hypertension, left ventricular hypertrophy, heart failure constitute of an important chain of events. Hypertension mainly leads to heart failure with preserved ejection fraction, while if combined with CHD or myocardial infarction, it can also lead to heart failure with reduced ejection fraction.

Hypertension is an important cause of atrial fibrillation.[32] Hypertension-atrial fibrillation-cerebral embolism constitute of an important but easily overlooked chain of events.

Long-term clinical follow-up studies revealed that the incidence of end-stage renal disease (ESRD) also increased significantly with clinic BP.[33] The incidence of ESRD was 11 times higher in severe hypertension, and even 1.9 times higher in subjects with high normal BP compared to that in normotensive subjects.

The relationship between clinic BP and the above-mentioned complications and cardiovascular diseases has also been confirmed in studies on ambulatory or home BP monitoring.[34,35] The 24-h ambulatory BP, nocturnal BP and morning BP were even more closely and significantly correlated with the risk of cardiovascular and cerebrovascular disease. Recent studies have also shown that long-term BP variation (BPV), which reflects BP fluctuations, may also be associated with cardiovascular risk.[35]

2.2 Cardiovascular risk characteristics of hypertension population in China

According to the population-based surveillance data in China,[36] cardiovascular and cerebrovascular diseases account for more than 40% of the total number of deaths. The annual incidence of stroke is 250/100,000, and the annual incidence of CHD events is 50/100,000. The incidence of stroke is five times higher than that of CHD events. In recent years, despite the ascending trend of the incidence of CHD events, the incidence difference between stroke and CHD events is still significant. In clinical trials, the incidence ratio of stroke/myocardial infarction is about 5:1–8:1 in hypertensives in China, while about 1: 1[37–41] in hypertensives in the Western countries. Therefore, stroke is still the major cardiovascular risk threatening the Chinese hypertensive population, and stroke prevention is an important goal of hypertension treatment in China.

3 Diagnostic evaluation

Diagnostic evaluation comprises the following three aspects: (1) to establish the diagnosis of hypertension, determine the stage of BP level; (2) to identify the etiology of hypertension, distinguish the primary or secondary hypertension; and (3) to search for other cardiovascular and cerebrovascular risk factors, target organ damage and their associated clinical conditions, so as to facilitate the differential diagnosis of hypertensive etiologies, assess the cardiovascular and cerebrovascular risk, and guide the diagnosis as well as the treatment.

3.1 Medical history

Patient’s medical history should be comprehensively and thoroughly understood, including the following contents: (1) family history: the patients should be asked whether they have a family history of hypertension, stroke, diabetes mellitus, dyslipidemia, CHD or kidney disease, including the age at which cardiovascular and cerebrovascular events occur in first-degree relatives. (2) Clinical course: the time and place where hypertension was first detected or diagnosed the highest level of BP. If having been treated with anti-hypertensive drugs, type, dose, efficacy and side effects of anti-hypertensive drugs used in the past and at present should be recorded. (3) Symptoms and past history: inquire about the present and past history, symptoms and treatment of stroke or transient ischemic attacks, CHD, heart failure, atrial fibrillation, peripheral vascular disease, diabetes, gout, dyslipidemia, sexual dysfunction and kidney disease, *etc*. (4) Secondary hypertension clues: such as nephritis history or anemia history; muscle weakness, paroxysmal soft paralysis, *etc*; paroxysmal headache, palpitation, sweating; snoring with apnea; whether long-term use of drugs which can result in BP elevation. (5) Lifestyle: the dietary intake of salt, alcohol and fat, smoking status, physical activity, weight change, sleep habits, *etc*. (6) Socio-psychological factors: including patient's family situation, work environment, education background and history of psychological trauma.

3.2 Physical examination

Careful physical examinations are helpful in finding clues of secondary hypertension and target organ damages. Physical examinations include: BP measurement (see BP measurement section 3.5 for details), measurement of heart rate, BMI, and waist and hip circumference; observing whether there are features of Cushing's syndrome, skin stigmata of neurofibromatosis, eye signs suggestive of hyperthyroidism, or edema at the lower extremities; searching for murmurs via the auscultation at the carotid arteries, thoracic aorta, abdominal aorta and the femoral arteries; palpating the thyroid glands, examining thoroughly the heart and lungs, examining the abdomen to find out whether there is enlargement of kidneys (polycystic kidney) or masses, palpating the arterial pulses at the extremities, and performing neurological examinations.

3.3 Laboratory examinations

Routine examinations: blood chemistry (potassium, sodium, fasting blood glucose, lipids, uric acid and creatinine), blood routine test, urinalysis (urine protein, sugar, sedimentary microscopic examination), electrocardiogram, *etc*.

Recommended examinations include: echocardiography,

carotid ultrasonography, oral glucose tolerance test, glycated hemoglobin, blood high-sensitivity C-reactive protein, urinary albumin/creatinine ratio, quantitative estimation of urinary protein, eye fundoscopy, chest X-ray, pulse wave velocity (PWV), and ankle-brachial BP index (ABI), *etc*.

Optional examinations include: blood homocysteine, for patients suspected of secondary hypertension. The following examinations might be performed if indicated: plasma renin activity or renin concentration, blood and urine aldosterone, blood and urine cortisol, blood free metanephrine and normetanephrine, blood or urine catecholamine, renal artery ultrasound and angiography, echogram, computed tomography (CT) or magnetic resonance imaging (MRI) of kidneys and adrenal glands, adrenal venous sampling, and polysomnography, *etc*. Hypertensives with comorbidity should be examined for relevant cardiac function, renal function and cognitive function.

3.4 Genetic analysis

Although genome-wide association studies (GWAS) of hypertension has reported a number of gene loci related to BP level or hypertension,[42] the current clinical genetic diagnosis is only applicable to Liddle syndrome,[43] glucocorticoid-remediable aldosteronism[44] and other monogenic inherited hypertension.

3.5 BP measurement

Key point 3A. Procedures for clinic BP measurement

- Patient should be seated for at least 5 min in a quiet room before BP measurements, and keep the upper arm at the heart level.
- Validated upper arm medical electronic sphygmomanometer is recommended, and mercury sphygmomanometer will be deprecated gradually.
- Use a standard cuff (with a bladder of 12 cm wide and 22–26 cm long) for most patients, larger cuffs should be used for obese patients or those with large arm circumference (> 32 cm).
- Measure BP on both arms at the first visit. Use the arm with higher value as the reference.
- Two BP measurements should be taken 1–2 min apart and averaged for records. An additional measurement is required if the first two readings differ by > 5 mmHg, and the mean value of the three readings should be recorded.
- Measure BP 1 min and 3 min after standing from a seated position in case of elderly patients, diabetic patients, and patients suspected orthostatic hypotension.
- Heart rate should be measured together with BP.

Key point 3B: evaluation of various BP measurement methods

- Clinic BP measurement is by now the common method to diagnose hypertension, grade the BP level category and observe the antihypertensive efficacy in China.
- If possible, out-of-office BP measurement should be performed to diagnose white-coat hypertension and masked hypertension, evaluate the efficacy of antihypertensive therapy, and assist the diagnosis and treatment of resistant hypertension.[45]
- Ambulatory BP monitoring can evaluate 24-h BP circadian rhythm, orthostatic hypotension, and postprandial hypotension, *etc*.
- Home BP monitoring may assist in adjusting treatment regimens. Remote real-time BP monitoring based on internet is a new mode of BP management. Patients with high psychological anxiety are not recommended to have their BP checked frequently.

BP measurement is the fundamental means and method of evaluating BP level, diagnosing hypertension and observing antihypertensive efficacy. Clinic and out-of-office BP measurement are adopted frequently in clinical practice and population disease prevention and control, the latter refers to ambulatory BP monitoring (ABPM) and home BP monitoring (HBPM). Out-of-office BP measurement can provide large amount of BP data outside the medical environment, and its relationship with target organ damage is more significant than that of clinic BP, and it is better than clinic BP in predicting cardiovascular risk.[34,46]

3.5.1 Clinic BP measurement

The clinic BP measurement is carried out by doctors or nurses, under standard conditions and unified guidelines. It is the common method to diagnose hypertension, grade the BP level category and observe the antihypertensive efficacy.

Upper arm medical electronic sphygmomanometer, which has been validated by international standardized protocols (ESH, BHS and AAMI) (electronic sphygmomanometer certification results are available in the following website: http://www.dableducational.org, http://www.bhsoc.org/default.stm), or mercury sphygmomanometer, which is up to the metrological standard (will be deprecated gradually), are used for the measurement. Automated office BP measurement (AOBP) can reduce the white-coat effect, which is worthy of further research and promotion.[47] See key point 3A for the measurement procedure. If mercury column sphygmomanometer is used for BP measurement, rapid inflation is required to let the inner pressure of the bladder go further up by 30 mmHg after the disappearance of the radial artery pulse downstream, and then deflate the bladder slowly at a speed of 2.0 mmHg per second. The deflation speed should be even slower if the heart rate is slow. Deflate the bladder quickly to zero after DBP reading is obtained; listen to the Korotkoff sound carefullly during the deflation of the bladder, watch the convex meniscus

surface of the mercury column at its perpendicular height at the moment of Korotkoff phase I (the first sound) and phase V (the disappearance of the sound). Take the reading at Korotkoff phase I as SBP and the reading at Korotkoff phase V as DBP. In the case of children (< 12 years of age), pregnant women, patients with severe anemia, hyperthyroidism, aortic valve incompetence, or with the Korotkoff sound not disappearing, take the Korotkoff sound of phase IV (when the sound changes abruptly) as DBP. When reading the BP value, the last digit value can only be 0, 2, 4, 6, 8, and cannot be 1, 3, 5, 7, 9, and pay attention to avoiding the last digit preference. When measuring BP in patients with atrial fibrillation, there is often a long period of Korotkoff sound auscultation interval, which requires multiple measurements to obtain the mean value.

3.5.2 ABPM

ABPM with the use of automatic device can acquire multiple BP readings, avoid white-coat effect and there is no observer error. It is possible to measure BP during sleep at night, identify white-coat hypertension and masked hypertension, and diagnose isolated nocturnal hypertension. At present, the major clinical use of ABPM are: to diagnose white-coat hypertension, masked hypertension and isolated nocturnal hypertension; to observe abnormal BP rhythm and variation; to evaluate the efficacy of antihypertensive therapy and BP control over all time periods (including morning and sleep). (1) ABPM should be validated using international standardization protocols, and be calibrated regularly.[45] (2) Usually the monitors are programmed to measure BP every 15 to 20 min during the day and every 30 min during sleep at night. Ensure that BP is monitored effectively throughout 24-h, with at least one BP reading per hour, at least 70% of expected valid BP readings, and at least 20 measurements during the day and 7 measurements at night. (3) ABPM indices: 24-h, daytime (awakening), nighttime (sleep) SBP and DBP mean values.

3.5.3 HBPM

BP is measured by the person who is being measured, and can also be done with the help of family members, known as self-measured BP or HBPM. HBPM can be used to evaluate anti-hypertensive efficacy and long-term BP variation for days, weeks, months or even years, and can help to enhance patient's awareness of health participation, improve patient's compliance and adherence to treatment, and is suitable for long-term BP monitoring of patients.[48] With the development of BP telemonitoring technology and equipment, internet-based remote monitoring and management of HBP is expected to become a new model of BP management in the future, but more studies are needed to provide evidence of effectiveness and cost-effectiveness.

HBPM[48,49] can be used for the BP monitoring of general hypertensive patients, so as to identify white-coat hypertension, masked hypertension and resistant hypertension, evaluate the long-term BP variation, assist in the assessment of anti-hypertensive efficacy, and predict cardiovascular risk and prognosis, *etc*. HBPM requires the selection of appropriate BP measurement devices, and patient education on BP self-measurement knowledge, skills and protocols. (1) Use upper-arm automatic electronic sphygmomanometer validated by international standardized protocols for home use. Wrist sphygmomanometer, finger sphygmomanometer, mercury column sphygmomanometer are not recommended for HBPM. Electronic sphygmomanometer should be calibrated regularly during use, at least once a year. (2) Monitoring protocol: for patients with firstly diagnosed hypertension or unstable hypertension, HBPM should be performed every morning and evening with 2–3 measurements each time and take the average; it is recommended to measure HBP for consecutive seven days and take the average BP of the last six days. Patients with stable and controlled BP are advised to have their BP measured one to two days a week, once in the morning and once in the evening. It is better to self-measure BP in the sitting position at fixed time after getting up in the morning, before taking antihypertensive medicines and breakfast, and after urination. (3) Keep a detailed record of the date and time of each BP measurement as well as all BP readings, not just the average. Complete BP records should be provided to the physician whenever possible. (4) HBPM is not recommended for patients with high psychological anxiety.

3.6 Evaluation of target organ damage

For patients with hypertension, the evaluation of target organ damage is an important part of the diagnostic evaluation of hypertension, especially the detection of asymptomatic subclinical target organ damage. Subclinical target organ damage can be reversed if detected early and treated timely. It is recommended to adopt methods, which are fit to individuals and local conditions, relatively simple, appropriate cost-effectiveness and easy to popularize, to screen and prevent subclinical target organ damage.

3.6.1 Heart

Left ventricular hypertrophy (LVH) is an independent risk factor for cardiovascular events. Routine examination methods include electrocardiogram and echocardiography. Electrocardiogram is simple and feasible, which can be used as a screening method for LVH. Commonly used parame-

ters are: Sokolow-Lyon voltage (SV1+RV5) and Cornell voltage-duration product.[50] The sensitivity of echocardiography in the diagnosis of LVH is superior to that of electrocardiogram. Left ventricular mass index (LVMI) can be used to detect and diagnose LVH, and LVMI is a strong predictor of cardiovascular events. Other methods of assessing hypertensive cardiac damage include chest X-ray, exercise test, cardiac isotope imaging, computed tomography coronary angiography (CTA), cardiac magnetic resonance imaging (MRI) and magnetic resonance angiography (MRA), coronary angiography, *etc*.

3.6.2 Kidney

Kidney damage is mainly characterized by elevated serum creatinine, reduced estimated glomerular filtration rate (eGFR), or increased urinary excretion of albumin. Microalbuminuria has been demonstrated as an independent predictor of cardiovascular events.[51] Hypertensive patients, especially those with diabetes mellitus, should be regularly monitored for urinary albumin excretion, 24-h urinary albumin excretion or urinary albumin/creatinine ratio. eGFR is a simple and sensitive indicator of renal function, which can be calculated by using "CKD-EPI formula",[52] "MDRD formula"[53] or MDRD modified formula[54] proposed by Chinese researchers. Increased serum uric acid level may also have predictive value for cardiovascular risk.[55]

3.6.3 Large arteries

Carotid intima media thickness (IMT) can predict cardiovascular events,[56] and atherosclerotic plaques are more predictive than IMT.[57] There is growing evidence showing that increased large arterial stiffness predicts cardiovascular risk. Increased pulse wave velocity (PWV) is a strong predictor of cardiovascular events and all-cause mortality. [58] Carotid-femoral PWV (cfPWV) is the gold standard of arterial stiffness measurement. [59] Ankle-brachial index (ABI) is effective in screening and diagnosing peripheral arterial disease and predicting cardiovascular risk.[60]

3.6.4 Eye fundoscopy

Retinal arteriopathy can reflect small vascular lesions, and fundoscopy is particularly important for patients with both hypertension and diabetes mellitus. Among hypertensive fundus changes detected by conventional fundoscopy, according to Keith-Wagener and Barker four-grade taxonomies, Grade 3 or Grade 4 retinopathy in hypertensives is valuable for prognosis.[61] Recently, new fundoscopy techniques have been used to observe and analyze the remodeling of retinal arterioles.[62]

3.6.5 Brain

Cranial MRA or CTA can help to detect lacunar lesions, asymptomatic cerebrovascular lesions (such as intracranial arterial stenosis, calcification and plaque lesions, and hemangioma), and white matter lesions,[63] but it is not recommended for clinical screening of target organ damage. Transcranial doppler ultrasonography is helpful for the diagnosis of cerebral vasospasm, stenosis or occlusion. At present, the screening and evaluation of cognitive function mainly adopts the mini-mental state examination.

4 Classification and stratification of hypertension

Key point 4

- Definition of hypertension: a clinic systolic BP ≥ 140 mmHg and/or diastolic BP ≥ 90 mmHg without the use of anti-hypertensive medications. Hypertension is divided into grade 1, grade 2 and grade 3 based on the BP levels;
- Cardiovascular risk was stratified according to BP level, cardiovascular risk factors, target organ damage, associated clinical complications and diabetes mellitus, which were divided into four levels: low risk, moderate risk, high risk and very high risk.

4.1 Classification by BP levels

At present, normal (SBP < 120 mmHg and DBP < 80 mmHg), high normal (SBP 120–139 mmHg and/or DBP 80–89 mmHg) and hypertension (SBP ≥140 mmHg and/or DBP ≥ 90 mmHg) are used to classify BP levels in China. The above classification applies to adults over 18 years of age.

In China, BP level of 120–139/80–89 mmHg is defined as 'high normal' mainly because our epidemiological studies have shown that the 10-year cardiovascular risk among this population is two times higher than those with BP < 110/75 mmHg. In addition, among the middle-aged population with a BP of 120–129/80–84 and of 130–139/85–89 mmHg, 45% and 64% respectively progressed to hypertension in ten years.[64]

Hypertension is defined as a clinic systolic BP ≥ 140 mmHg and/or diastolic BP ≥ 90 mmHg without the use of anti-hypertensive medications at three visits on different days. Systolic BP ≥ 140 mmHg and diastolic BP ≤ 90 mmHg is defined as isolated systolic hypertension. Subjects with a BP < 140/90 mmHg but having hypertensive history and currently are taking anti-hypertensive medication should also be diagnosed as hypertensives. Hypertension is divided into grade 1, grade 2 and grade 3 based on the levels of BP (Table 5). The diagnostic thresholds for hypertension

Table 5. Definitions and classification of BP levels

Category	SBP, mmHg	DBP, mmHg
Normal	< 120	< 80
High normal	120–139 and (or)	80–89
Hypertension	≥ 140 and (or)	≥ 90
Grade 1 (mild)	140–159 and (or)	90–99
Grade 2 (moderate)	160–179 and (or)	100–109
Grade 3 (severe)	≥ 180 and (or)	≥ 110
Isolated systolic hypertension	≥ 140 and	< 90

based on ABPM is: 24-h mean SBP/DBP ≥ 130/80 mmHg; daytime SBP/DBP ≥ 135/85 mmHg; nighttime SBP/DBP ≥ 120/70 mmHg. The diagnostic thresholds of hypertension based on HBPM ≥ 135/85 mmHg, which is corresponding to 140/90 mmHg of clinic BP.

When a patient's systolic and diastolic BPs fall separately into different categories, the higher category should be taken. BP: blood pressure; DBP: diastolic blood pressure; SBP: systolic blood pressure.

Due to the low frequency of clinic BP measurement and the obvious fluctuation of BP, it is necessary to take multiple measurements within a few weeks to evaluate BP elevation, especially for grade 1 and grade 2 hypertension. If possible, 24-h ABPM or HBPM should be conducted.

4.2 Cardiovascular risk stratification

Although hypertension is an independent risk factor for the incidence and prognosis of cardiovascular events, it is not the only determinant. Most hypertensive patients have cardiovascular risk factors other than elevated BP. Therefore, the diagnosis and treatment of hypertension should base not only BP levels, but also comprehensive cardiovascular risk assessment and stratification. Cardiovascular risk stratification in hypertensive patients may help in determining appropriate time of starting antihypertensive therapy, optimizing antihypertensive therapy, establishing appropriate BP control target and conducting comprehensive management of patients.

For risk stratification, this guideline adopts the principle and basic contents of the 2005 and 2010 Chinese Hypertension Guidelines.[64,65] Patients with hypertension are divided into four levels of cardiovascular risk: low risk, moderate risk, high risk and very high risk (Table 6). According to the implementation of the previous Chinese hypertension guidelines and relevant research progress, the contents affecting risk stratification have been partially modified (Table 7): BP range of 130–139/85–89 mmHg has been added; diagnostic criteria for hyperhomocysteinemia has been modified as ≥ 15 umol/L; inclusion of atrial fibrillation as a concomitant clinical disease; diabetes mellitus was divided into newly diagnosed and treated but not controlled according to the level of blood glucose (fasting and postprandial) and glycated hemoglobin, respectively.

5 Treatment of hypertension

5.1 Goal of hypertension treatment

Key point 5A

- The fundamental goal of hypertension treatment is to reduce the overall risk of developing heart, brain, kidney and vascular complications and death.
- The benefits of antihypertensive treatment derive primarily from the BP reduction *per se*.
- On the basis of lifestyle improvement, antihypertensive drugs should be administered according to the overall risk level of hypertensive patients, while intervening in correctable risk factors, target organ damage and coexisting clinical diseases.
- Intensive antihypertensive treatment strategy should be adopted to achieve maximum cardiovascular benefit if conditions allowed.
- Goal of anti-hypertensive treatment: for general hypertensive patients, their BP should be reduced to < 140/90 mmHg (Ⅰ, A),[1,40,66] and further lower level (< 130/80 mmHg) could also be applicable if patients can tolerant it or belongs to high-risk category (Ⅰ, A).[37,38,67,68]

Table 6. Cardiovascular risk stratification in patients with elevated BP.

Other risk factors and medical history	BP, mmHg: SBP 130–139 and (or) DBP 85–89	SBP 140–159 and (or) DBP 90–99	SBP 160–179 and (or) DBP 100–109	SBP ≥ 180 and (or) DBP ≥ 110
No other risk factors		Low risk	Moderate risk	High risk
1–2 risk factors	Low risk	Moderate risk	Moderate to high risk	Very high risk
≥ 3 risk factors, TOD or CKD grade 3 or diabetes mellitus without organ damage	Moderate/high risk	High risk	High risk	Very high risk
Clinical complications, or CKD grade ≥ 4, or diabetes mellitus with organ damage	High/very high risk	Very high risk	Very high risk	Very high risk

BP: blood pressure; CKD: chronic kidney disease; DBP: diastolic blood pressure; SBP: systolic blood pressure; TOD: target organ damage.

Table 7. Important factors influencing cardiovascular prognosis in hypertensive patients.

Cardiovascular risk factors	TOD	Concomitant clinical diseases
· Hypertension (Grade 1–3)<br>· Man > 55 years<br>· Woman > 65 years<br>· Smoking or passive smoking	· Left ventricular hypertrophy<br>electrocardiogram: Sokolow-Lyon voltage > 3.8 mV or Cornell product > 244 mV·ms<br>Echocardiogram: LVMI (man ≥ 115 g/m^2, woman ≥ 95 g/m^2)	· Cerebrovascular disease<br>Cerebral hemorrhage<br>Ischemic stroke<br>Transient ischemic attack
· Impaired glucose tolerance (7.8–11.0 mmol/L for 2-h blood glucose) and/or impaired fasting glucose (6.1–6.9 mmol/L)	Carotid ultrasonography (IMT ≥ 0.9 mm) or atherosclerotic plaque	· Heart disease<br>History of myocardial infarction<br>Angina pectoris<br>Coronary revascularization<br>Congestive heart failure<br>Atrial fibrillation
· Dyslipidemia<br>TC ≥ 6.2 mmol/L (240 mg/dL) or LDL-C ≥ 4.1 mmol/L (160 mg/dL) or HDL-C < 1.0 mmol/L (40 mg/dL)	· Carotid-femoral pulse wave velocity ≥ 12 m/s (*optional)<br>· Ankle/Brachial index < 0.9 (*optional)	· Renal disease:<br>Diabetic nephropathy<br>Renal dysfunction<br>Including eGFR < 30 mL/min per 1.73 m^2; elevated serum creatinine: man ≥ 133 μmol/L (1.5 mg/dL), woman ≥ 124 μmol/L (1.4 mg/dL); proteinuria: (≥ 300 mg/24 h)
· Family history of early onset cardiovascular disease (onset of first-degree relatives at age < 50 years)<br>· Abdominal obesity (waist circumference: Man ≥ 90 cm, Woman ≥ 85 cm) or obesity (BMI ≥ 28 kg/m^2)	· Reduced estimated glomerular filtration rate (eGFR 30–59 mL/min per 1.73 m^2) or slight increase in serum creatinine:<br>Man 115–133 μmol/L (1. 3–1. 5 mg/dL,<br>Woman 107–124 μmol/L (1.2–1.4 mg/dL)	· Peripheral vascular disease<br>· Advanced retinopathy:<br>Hemorrhages or exudates;<br>Papilloedema
· Hyperhomocysteinemia (≥ 15 μmol/L)	· Microalbuminuria: 30–300 mg/24 h or albumin/creatinine ratio ≥ 30 mg/g (3.5 mg/mmol)	· Diabetes mellitus<br>Newly diagnosed:<br>Fasting blood glucose ≥ 7.0 mmol/L (126 mg/dL);<br>postprandial blood glucose ≥ 11.1 mmol/L (200 mg/dL)<br>Treated but not controlled:<br>Glycated hemoglobin: (HbA1c) ≥ 6.5%

BMI: body mass index; eGFR: estimated glomerular filtration rate; HDL-C: high-density lipoprotein; IMT: intima media thickness; LDL-C: low-density lipoprotein; LVMI: left ventricular mass index; TC: total cholesterol; TOD: Target organ damage.

The fundamental goal of hypertension treatment is to reduce the overall risk of developing heart, brain, kidney and vascular complications and the occurrence of death. Considering that hypertension is a cardiovascular syndrome, which is often combined with other cardiovascular risk factors, target organ damage and clinical disease, it is necessary to determine the timing and intensity of lifestyle improvement and antihypertensive drugs based on the BP level and overall risk level of hypertensive patients; meanwhile, it is also essential for intervening in other risk factors, target organ damage and coexisting clinical diseases that are detected. Given the unchanged situation in China that the major complication of hypertensive patients is still stroke,[36] intensive antihypertensive treatment strategy should therefore be adopted when conditions permit.

Based on the evidence from previous studies,[2,3,15,40] the goal of BP control should be managed below 140/90 mmHg in general patients; under tolerable and sustainable conditions, some patients with diabetes, proteinuria, *etc.* who belong to high-risk category can control their BP below 130/80 mmHg. Although evidence suggests lower or higher BP goal in special population exists,[37,38] but this mainly depends on the patients' tolerance and the complexity of their treatment. If a patient can reduce BP to lower level without carrying out a complex treatment regimen and can tolerate it, then there is no need to change the regimen to get the BP restored.

When selecting the kind and the intensity of treatment regimen, it should be balanced between long-term benefits and patient tolerability, so as to reduce or avoid withdrawal due to the patients' intolerance. Intensive interventions for high-risk and very-high-risk patients are justified, as well as aggressive interventions to reverse target organ damage in patients with subclinical target organ damage without severe comorbidities. However, when antihypertensive drugs are administered to high-normal BP populations with low or moderate risk, there is still insufficient evidence from clinical trials with the prognostic endpoint as the research objective.

Although some studies[39,69,70] have shown that elderly hypertensive patients' BP have a higher goal than those general patients, recent subgroup analysis of some studies also showed that a lower BP goal (SBP < 130 mmHg) is beneficial to the elderly population.[38] It should be noted that age growth is not a sufficient condition for setting a higher goal for antihypertensive treatment. For elderly patients, based on the severity of patients' comorbidities, physicians should evaluate the compliance and the adherence to treatment comprehensively, and determine their treatment goals of BP.

5.2 Therapeutic strategies

Key point 5B

- The way to achieve antihypertensive treatment target: in addition to hypertensive emergencies and hypertensive urgencies, most hypertensive patients' BP should be gradually reduced to the target level within 4 weeks or 12 weeks according to their condition (Ⅰ, C).
- Timing of antihypertensive drug therapy: on the basis of lifestyle improvement, patients with BP still ≥ 140/90 mmHg and/or above target BP should initiate drug therapy (Ⅰ, A).

(1) Target of antihypertensive treatment: the purpose of antihypertensive treatment for hypertensive patients is to effectively prevent or delay the occurrence of complications such as stroke, myocardial infarction, heart failure and renal insufficiency by reducing their BP, and to effectively control hypertension's progression and prevent the occurrence of severe hypertension such as hypertensive emergencies and hypertensive urgencies. Earlier antihypertensive trials with the inclusion criteria of DBP (≥ 90 mmHg) showed that each 5 mmHg lowering in DBP (10 mmHg lowering in SBP) would result in a decrease of stroke risk by 40% and ischemic heart disease by 14%, respectively;[71] later trials of antihypertensive treatment for isolated systolic hypertension (SBP ≥ 160 mmHg, DBP < 90 mmHg) showed that each 10 mmHg lowering in SBP (4 mmHg lowering in DBP) would result in a decrease of stroke risk by 30% and ischemic heart disease by 23%, respectively.[72] Recent studies, such as Systolic Blood Pressure Intervention Trial (SPRINT), Action to Control Cardiovascular Risk in Diabetes (ACCORD) also showed that intensive BP control is beneficial for patients of different age groups and/or patients with heart, kidney or diabetes comorbidities.[37,38]

(2) The way to achieve antihypertensive treatment target: reducing BP to the target level can significantly reduce the risk of cardio-cerebrovascular complications. In addition to hypertensive emergencies and hypertensive urgencies, most hypertensive patients' BP should be gradually reduced to the target level within 4 weeks or 12 weeks according to their condition. For the young and for patients with shorter course of hypertension, BP might be reduced faster, whereas for elderly patients with longer course of hypertension, poor tolerability and combined with comorbidities, BP might be reduced slightly slower. Subgroup analysis of the FEVER study suggested that after medication, patients who reach the target BP within one month may further reduce their risk of cardiovascular events than those who meet the criteria behind that time.

(3) Timing of antihypertensive drug therapy: the timing of antihypertensive drug therapy depends on the level of cardiovascular risk assessment. On the basis of lifestyle improvement, patients with BP still ≥ 140/90 mmHg and/or above target BP should initiate drug therapy. For patients with high and very high risk, drug therapy must be started soon, and comprehensive treatment should be performed for the associated risk factors and clinical conditions; patients with moderate risk can be observed for several weeks to assess target organ damage and improve lifestyle, if target BP is still not achieved, drug therapy ought to be started; patients with low risk, can be observed for one to three months with a close follow-up, performing out-of-office BP monitoring whenever possible, assessing target organ damage and improving lifestyle, if target BP could still not be achieved, drug therapy can be started.

This strategy should be followed especially for newly diagnosed hypertensive patients. The assessment and monitoring procedures are shown in Figure 2.

5.3 Lifestyle intervention

Key point 5C

- Lifestyle interventions are reasonable and effective treatments for any hypertensive patients (including normal high-value patients and hypertensive patients requiring medication) at any time, aim to lower BP, control other risk factors and clinical conditions.
- Lifestyle interventions have positive effects towards reducing BP and cardiovascular risk, [73–75] which should be used in all patients. The main measures include:

To reduce sodium intake, gradually reduce the daily salt intake to < 6 g, and increasing potassium intake (Ⅰ, B)[74–79]

Reasonable meal, balanced diet (Ⅰ, A)[80–83]

To control body weight to make BMI < 24, and to make waist circumference < 90 cm

for male and < 85 cm for female (Ⅰ, B)[84–86]

Do not smoke, completely quit smoking, and avoid passive smoking (Ⅰ, C).[87,88]

Do not drink or restrict alcohol (Ⅰ, B)[22,89–91]

To increase exercise, medium intensity; 4–7 times per week; 30–60 min each time (Ⅰ, A)[92–94]

To reduce mental stress and maintain psychological balance (Ⅱ a, C)[95]

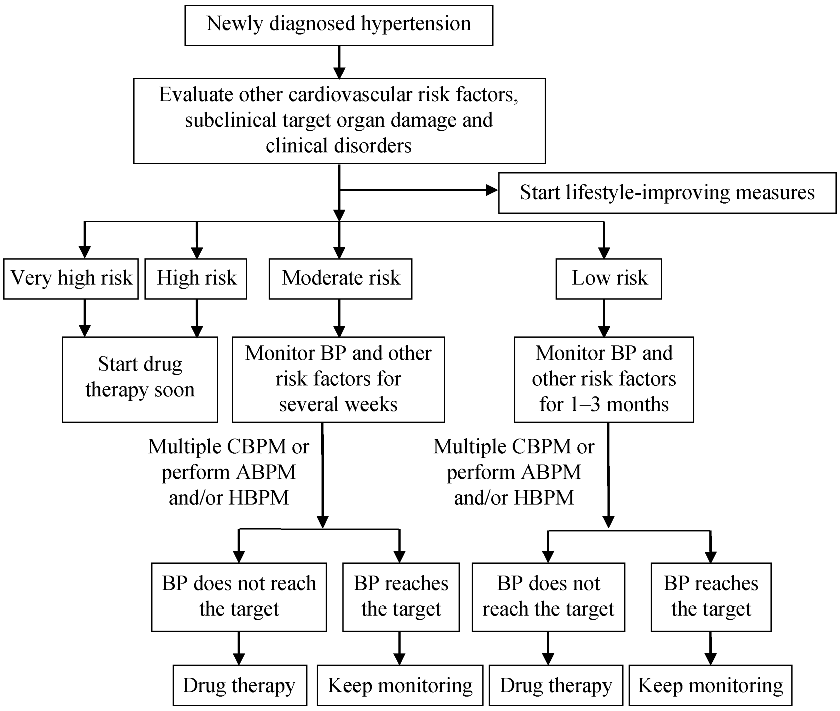

Figure 2. Evaluation and monitoring procedures for newly diagnosed hypertension. Diagnostic criteria of hypertension for ABP is daytime mean SBP ≥ 135 mmHg or DBP ≥ 85 mmHg, nighttime mean SBP ≥ 120 mmHg or DBP ≥ 70 mmHg, or 24-h mean SBP ≥ 130 mmHg or DBP ≥ 80 mmHg; that criteria for home blood pressure is mean SBP ≥ 135 mmHg or DBP ≥ 85 mmHg. Moderate risk patients with BP ≥ 160/100 mmHg should start drug therapy immediately. ABPM: ambulatory blood pressure monitoring; BP: blood pressure; CBPM: clinic blood pressure monitoring; DBP: diastolic blood pressure; HBPM: home blood pressure monitoring; SBP: systolic blood pressure.

Lifestyle interventions can reduce BP, prevent or delay the onset of high BP, and reduce the risk of cardiovascular disease.[96–99] In this guideline, lifestyle interventions include promoting healthy lifestyles and eliminating behaviors and habits that are unconducive to physical and mental health. Lifestyle interventions should be continued throughout the entire process of hypertension treatment, in combination with medication,[100] if necessary. The details are as follows.

5.3.1 Sodium intake reduction and potassium intake increase

Sodium salt can significantly increase BP and the risk of hypertension, and the moderate reduction of sodium intake can effectively lower BP.[75,79] Excessive sodium intake and/or insufficient potassium intake, as well as a low ratio of potassium to sodium intake are important risk factors for hypertension in China.[101,102]

Around 75.8% of the sodium in the diet of Chinese residents comes from household cooking salt, followed by high-salt seasoning. As dietary patterns change, sodium salt in processed foods will also become important pathways for sodium intake.[103] In order to prevent high BP and reduce BP in hypertensive patients, sodium intake should be reduced to 2400 mg/day (6 g sodium chloride). All hypertensive patients should take various measures to limit sodium intake. The main measures include: (1) reducing cooking salt and sodium-containing condiments (including monosodium glutamate, soy sauce); (2) avoiding or reducing processed foods with high sodium content, such as pickles, ham, all kinds of roasted seeds and vegetables; and (3) it is recommended to use a quantitative salt spoon as much as possible during cooking to serve as a warning.

Increasing potassium intake in the diet can reduce BP.[74] The main measures are: (1) increasing the intake of potassium-rich foods (fresh vegetables, fruits and beans); and (2) individuals with good kidney function can choose low-sodium potassium-rich alternative salts. It is not recommended to take potassium supplements (including drugs) to reduce BP. Patients with renal insufficiency should consult a doctor before potassium supplementation.

5.3.2 Reasonable diet

A reasonable diet mode can reduce the risk of hypertension and cardiovascular disease in the population.[80–83] Hypertensive patients and those with normal BP who are at risk of developing hypertension are recommended that the diet

should be based on fruits, vegetables, low-fat dairy products, whole grains rich in dietary fiber, and protein derived from plants to reduce saturated fat and cholesterol intake. DASH (Dietary Approaches to Stop Hypertension) diet is rich in fresh vegetables, fruits, low-fat (or skim) dairy products, poultry, fish, soybeans and nuts, it has less sugar, sugary drinks and red meat, it also has low levels of saturated fat and cholesterol, and it is rich in trace elements such as potassium, magnesium and calcium, high quality protein and cellulose.[80] For hypertensives, DASH diet can reduce SBP 11.4 mmHg and DBP 5.5 mmHg respectively,[80] the general population can reduce SBP 6.74 mmHg and DBP 3.54 mmHg respectively. If hypertensives control caloric intake, BP drop will be greater.[81] Compliance with DASH diet can effectively reduce the risk of CHD and stroke.[82,83]

5.3.3 Weight control

It is recommended to maintain weight in a healthy range (BMI: 18.5–23.9 kg/m^2, waist circumference < 90 cm for male, < 85 cm for female).[104] All overweight and obese patients were recommended to lose weight. Weight control is an intervention that includes controlling calorie intake, increasing physical activity, and behavioral interventions. Firstly, calorie intake control should include reducing daily total calorie intake, controlling intake of high-calorie foods (high-fat foods, sugary drinks and alcohol), and proper control of carbohydrate intake, and they all base on dietary balance. Then, increasing physical activity is advancing regular moderate-intensity aerobic exercise and reducing sedentary time. In addition, behavior therapy, such as establishing diet awareness, making meal plans, recording the type and weight of food intake, and calculating calories, can help to reduce weight. For those who are not ideal for weight loss in comprehensive lifestyle interventions, it is recommended to use medication or surgery. For special individuals, such as lactating women and the elderly, individualized weight loss measures should be used as appropriate.[105] The weight loss plan should be adhered to in the long term, the speed varies from person to person, no quick results should be rushed for. It is recommended to make a target that losing 5% to 10% of the initial weight in a year.[106,107]

5.3.4 Smoking cessation

Smoking is an unhealthy behavior and one of the major risk factors for cardiovascular disease and cancer. Passive smoking significantly increases the risk of cardiovascular disease.[108] Although smoking cessation does not help for controlling BP,[109] it can reduce the risk of cardiovascular disease.[88]

The benefits of quitting smoking are very positive, therefore, physicians should strongly recommend and urge patients with hypertension to quit smoking, they also should ask each patient about the daily amount and smoking habits, and then, advise them to quit smoking in a clear, strong, and personalized way. After assessing the smoker's willingness to quit, physicians should help the smoker to start smoking cessation with the "sudden stop method" after 1–2 week preparation period. At the same times, they should instruct patients to apply smoking cessation drugs for withdrawal symptoms, such as nicotine patches, nicotine chewing gum (over-the-counter), bupropion hydrochloride sustained-release tablets and varenicline. Above all, following up and supervising successful quitters to avoid relapse.

5.3.5 Restrict drinking

Excessive drinking significantly increases the risk of developing hypertension, and its risk increases with the increasing consumption of alcohol. Limiting drinking could reduce hypertension. Hypertension patients are not advised to drink alcohol. In case of drinking, better mild wine than strong wine. Daily alcohol intake does not exceed 25 g for men and 15 g for women. The weekly alcohol intake does not exceed 140 g for men and 80 g for women.[110] Liquor, wine, and beer intake are less than 50 mL, 100 mL, and 300 mL,[22] respectively.

5.3.6 More exercise

Exercise can improve BP levels. Aerobic exercise reduced SBP by 3.84 mmHg, DBP 2.58 mmHg on average.[94] Cohort studies have found that regular exercise in hypertensives reduces the risk of cardiovascular death and all-cause mortality.[93] Therefore, it is recommended that non-hypertensives who want to reduce the risk of high BP or hypertensive who is willing to reduce BP should execute moderate-intensity exercise with accumulated for 30 to 60 min per day, 4 to 7 days per week,[111] such as walking, jogging, cycling, swimming, *etc*. The types of exercise can be categorized under aerobic, impedance and stretching.[94] Aerobic exercise is the priority type, and anaerobic exercise as the supplementary type. Exercise intensity must vary from person to person. The maximum heart rate during exercise is used to assess exercise intensity. Medium-intensity exercise is an exercise about 60% to 70% of achieving maximum heart rate [maximum heart rate (times/min) = 220 – age]. High-risk patients need to be evaluated before exercise.

5.3.7 Reducing stress and maintain psychological balance

Mental stress can activate the sympathetic nerves which could raise hypertension,[24,25] the main reasons of increas-

ing mental stress include excessive work, life stress and morbid psychology. Morbid psychology covers depression, anxiety, Type A personality, social isolation, lack of social support and so on. Doctors should manage stress of hypertensives by guiding them to individualized cognitive behavioral interventions. If necessary, doctors could use psychotherapy combined with medication to relieve anxiety and stress in patients. Drugs that are primarily suitable for anxiety disorders include benzodiazepines which include alprazolam, lorazepam and selective 5-HT1A receptor agonists such as buspirone, tandospirone and so on. Patients can also be advised to visit a professional medical institution to avoid BP fluctuations due to mental stress.

5.4 Pharmacological therapy of hypertension

Key point 5D. Basic principles of anti-hypertensive treatment

- All five classes of anti-hypertensive drugs commonly used are suitable for the initiation of anti-hypertensive therapy. It is recommended that the choice of specific drugs should be based on the type of special population and comorbidities so as to the individualized treatment.
- The intimal selection between mono-therapy and combination therapy should be based the baseline BP and the cardiovascular risk factors.
- Ordinary patients start with regular dose, while the elderly is initiated with lower effective therapeutic dose. It can be considered to gradually titrate to the full dosage according to the treatment demand.[70,112–115]
- It is preferred to use long-acting agents to control 24-h BP, which can prevent cardio- and cerebrovascular complications more effectively.[40,116–118]
- It is recommended that high-risk group of patients with BP ≥ 160/100 mmHg or 20/10 mmHg higher than that of the target BP, or patients who receive mono-therapy and do not achieve the goal BP should be treated with combination therapy (Ⅰ, C), including fixed combination preparations or a free combination of two or more agents.[119]
- It is feasible to initiate with small dose combination therapy for patients with BP ≥ 140/90 mmHg (Ⅰ, C). [67,120–123]

5.4.1 Clinical trail basis of anti-hypertensive treatment

Since the 1950s, a series of randomized controlled trials of anti-hypertensive therapy which mainly targeted cardio- and cerebrovascular complications has established a theoretical basis for the treatment and management of hypertension.

These clinical trials can be divided into four types. (1) Early trials, which had a placebo control or untreated control, has demonstrated anti-hypertensive treatment can significantly reduce the risk factors of cardio- and cerebrovascular complications in various group of patients.[71,72] These studies have established the most important theoretical basis for the treatment and management of hypertension.

(2) The compared trials involving different types of anti-hypertensive regimens mainly focus on the comparison between newer type of anti-hypertensive agents drugs (CCB, ACEI and ARB) and traditional ones (thiazide diuretics, β-blockers, *etc*.). The outcome reveals that reducing BP *per se* is the most important reason for these drugs to reduce cardio- and cerebrovascular complications.[124,125] Differences between antihypertensive agents are generally small, but their effects on cause-specific outcomes still exist, and there are also differences in the results of different combination therapy trials.

(3) Trials taking hypertensive patients as the research subjects target to certain the most appropriate goal BP to treat hypertension by comparing the effect of intensive blood-pressure control and that of standard treatment.[37,38,126]

(4) Trials taking patients with moderate or high cardiovascular risk as the research subjects suggest that after BP is controlled under the target level of 140/90 mmHg, to further reduce BP should be tailored to individual conditions, which demands taking the patient's disease characteristics and the treatment plan including its composition and implementation method into full consideration.

China has also independently completed a series of clinical trials of antihypertensive therapy and contributed to several international multi-center clinical trials. Three early trials Syst-China,[39,127] STONE[128] and CNIT[129] all confirmed that active anti-hypertensive treatment based on calcium channel blockers (CCBs, such as nitrendipine, nifedipine, *etc*.) can significantly reduce Chinese hypertensive patients' incidence and mortality of stroke. Furthermore, the FEVER trial showed that although the SBP/DBP decrease is as small as 4/2 mmHg induced by the add-on therapy of felodipine to hydrochlorothiazide, the incidence of fatal and non-fatal stroke was markedly reduced by 27%.[42] Further subgroup analysis of the FEVER revealed that the stroke, cardiac events and total mortality were lowest when the mean BP level was below 120/70 mmHg after treatment.[66] The elderly patients with SBP < 140 mmHg benefit more from anti-hypertensive therapy than those of higher BP. The study phase report of CHIEF documented that initial treatment of low-dose amlodipine combination with telmisartan or compound amiloride can significantly reduce the BP level in hypertensive patients, with the control rates reached about 80%, which suggests CCB-based combination therapy is one of the optimal antihypertensive medication for Chinese hypertensive patients.[120]

The Post-stroke Antihypertensive Treatment Study (PATS) completed by China independently was the first large-scale, placebo-controlled clinical trial of antihypertensive therapy for the post-stroke secondary prevention in the world. The results showed a decrease of 5/2 mmHg and 29% recurrence in the BP and stroke respectively induced by indapamide (2.5 mg/day) compared with the placebo.[130,131] Thereafter, the result of the perindopril protection against recurrent stroke study (PROGRESS) in which China has cooperated showed that perindopril-based therapy reduced the recurrence of stroke by 28%, and the combination with indapamide produced larger reductions in BP than mono perindopril did.[132] The outcome of subgroup analysis showed that the risk of stroke reduction was larger in Asian participants such as the Chinese and the Japanese.[133] The *post-hoc* analysis determined that no "J-curve" phenomenon was observed even when the average BP fell to 112/72 mmHg after the treatment.[134] A further study was followed up in the 1560 enrolled Chinese patients and the average 6-year data confirmed that antihypertensive treatment significantly reduced the risk of stroke recurrence, total mortality and myocardial infarction.[135]

Chinese also participated in the three international multi-center clinical trials: "the Hypertension in the Very Elderly Trial" (HYVET),[70] "the Action in Diabetes and Vascular disease: preterAx and diamicroN-MR Controlled Evaluation" (ADVANCE)[122] and "the Heart Outcomes Prevention Evaluation" (HOPE-3).[67] The HYVET study showed that hypertension treatment based on indapamide (sustained release) in the very elderly (≥ 80 years old) with SBP above 160 mmHg, aimed to achieve a target BP of 150/80 mmHg, is beneficial and is associated with reduced risks of death from stroke and any cause.[70] The ADVANCE trial showed that compared to antihypertensive conventional treatment, a low-dose fixed combination of perindopril/indapamide produced a reduction of 5.6/2.2 mmHg and down to 135/75 mmHg in patients with diabetes, lowering the combined endpoints rate of major macrovascular or microvascular event by 9%.[122]

In the HOPE-3 trial, treatment with candesartan plus hydrochlorothiazide lowered BP by 6.0/3.0 mmHg as compared with placebo. Patients in the treatment group with SBP above 143.5 mmHg had significantly lower rates of cardiovascular risk than those in the placebo group. By contrast, no reduction of cardiovascular risk was observed among the patients in the active-treatment group who had an SBP below 131.5 mmHg.[67] The SPRINT study, mainly recruiting the US hypertensive patients, was a clinic trial conducted with a strategy of intensive blood-pressure control by using multiple antihypertensive drugs. Patients in the intensive-treatment group with the mean SBP reduced to 121 mmHg had lower incidence of cardio- and cerebrovascular complications, especially in the rates of heart failure, compared with those in the standard-treatment group with the mean SBP reduced to 135 mmHg.[38]

Hyperhomocysteinemia is positively associated with stroke risk.[136–138] Clinical trials conducted in China (such as multivitamin treatment trials,[139] meta-analysis of folic acid treatment trials[140] and CSPPT–China stroke primary prevention trial[41,141,142]) have shown that supplementation with folic acid can reduce plasma homocysteine concentration and reduce the risk of stroke. However, the role of folic acid in the prevention of stroke in hypertensive patients with high homocysteine still requires multi-center clinical trials for further validation.

5.4.2 Principles of drug treatment

(1) Initial dose: Ordinary patients start with regular dose, while the elderly is initiated with lower effective therapeutic dose. It can be considered to gradually titrate to the full dosage according to the treatment demand.[70,113–116]

(2) Long-acting agents: it is preferred to use long-acting agents to control 24-h BP, which can prevent cardio- and cerebro-vascular complications more effectively.[40,117–120] If intermediate-acting or short-acting agents are used, they should be administered two to three times per day in order to achieve a smooth BP control.

(3) Combination therapy: it is recommended that high-risk group of patients with BP ≥ 160/100 mmHg and 20/10 mmHg higher than that of the target BP, or patients who receive mono-therapy and do not achieve the goal BP should be treated with combination therapy, including fixed combination preparations or a free combination of two or more agents.[71] For patients with BP ≥ 140/90 mmHg, it is also feasible to initiate with small dose combination therapy.[67,122–125]

(4) Individualized treatment: the choice of anti-hypertensive drugs should be based on the individual conditions of the patient, the patients' comorbidities, drug efficacy and tolerance, as well as the personal will and the long-term financial reach.

(5) Pharmacoeconomics: hypertension requires lifelong treatment and the cost/benefit should be taken into consideration.

5.4.3 Classes of anti-hypertensive drugs

Currently recommended anti-hypertensive drugs include five classes, *i.e.*, calcium channel blockers (CCB), angio-

tensin-converting enzyme inhibitors (ACEI), angiotensin receptor blockers (ARB), diuretics, β-blockers, and fixed-ratio compound preparations composed of the above drugs.

All the 5 classes of anti-hypertensive drugs are suitable for the initiation and maintenance of anti-hypertensive therapy. Rational use of drugs should be based on patient risk factors as well as target organ subclinical damage plus clinical disease, and give preference to certain types of these drugs.[143,144] (Tables 8 & 9). These clinical conditions can be referred to as strong indications (Table 10). Sometimes α-blockers and other types of antihypertensive drugs can also be used in certain hypertension populations. The selection criteria of these anti-hypertensive drugs for clinical practice are listed Table 11.

Table 8. Anti-hypertensive drugs commonly available.

Oral agents	Does per day, mg (initiation–full)	Times per day	Major side effects
Dihydropyridines CCB			Ankle edema, headache and flushing
Nifedipine	10–30	2–3	
Slow release	10–80	2	
Control release	30–60	1	
Amlodipine	2.5–10	1	
Levamlodipine	2.5–5	1	
Felodipine	2.5–10	2	
Slow release	2.5–10	1	
Lacidipine	4–8	1	
Nicardipine	40–80	2	
Nitrendipine	20–60	2–3	
Benidipine	4–8	1	
Lercanidipine	10–20	1	
Manidipine	5–20	1	
Cinildipine	5–10	1	
Barnidipine	10–15	1	
Non-dihydropyridines CCB			A-V block and negative inotropic cardiac effect
Verapamil	80–480	2–3	
Slow release	120–480	1–2	
Diltiazem	90–360	1–2	
Thiazide diuretic			Hypokalemia, hyponatremia and hyperuricemia
Hydrochlorothiazide	6.25–25	1	
Chlorthalidone	12.5–25	1	
Indapamide	0.625–2.5	1	
Slow release	1.5	1	
Loop diuretics			Hypokalemia
Furosemide	20–80	1–2	
Torasemide;	5–10	1	
Potassium sparing diuretics			Hyperkalemia
Amiloride	5–10	1–2	
Triamterene	25–100	1–2	
Aldosterone receptor antagonists			
Spirolactone	20–60	1–3	Hyperkalemia and gynecomastia
Eplerenone	50–100	1–2	
β-blockers			Bronchospasm and negative inotropic cardiac effect
Bisoprolol	2.5–10	1	
Metoprolol	50–100	2	
Slow release	47.5–190	1	
Atenolol	12.5–50	1–2	
Propranolol	20–90	2–3	
Betaxolol	5–20	1	

Table 8. Cont.

Oral agents	Does per day, mg (initiation–full)	Times per day	Major side effects
α- and β-blockers			Postural hypotension and bronchospasm
Labetalol	200–600	2	
Cavedilol	12.5–50	2	
Arotinolol	10–20	1–2	
ACEI			Coughing, hyperkalemia and angioedema
Captopril	25–300	2–3	
Enalapril	2.5–40	2	
Benazepril	5–40	1–2	
Lisinopril	2.5–40	1	
Ramipril	1.25–20	1	
Fosinopril	10–40	1	
Cilazapril	1.25–5	1	
Perindopril	4–8	1	
Imidapril	2.5–10	1	
ARB			Hyperkalemia and angioedema (rare)
Losartan	25–100	1	
Valsartan	80–160	1	
Irbesartan	150–300	1	
Telmisartan	20–80	1	
Candesartan	4–32	1	
Olmesartan	20–40	1	
Allisartan Isoproxil	240	1	
α-blockers			Postural hypotension
Doxazosin	1–16	1	
Prazosin	1–10	2–3	
Terazosin	1–20	1–2	
Centrally acting agents			
Reserpine	0.05–0.25	1	Nasal congestion, depression, bradycardia, and digestive ulceration
Clonidipine	0.1–0.8	2–3	Hypotension, xerostomia, drowsiness
Clonidipine patch	0.25	1/week	Skin allergy
Methyldopa	250–1000	2–3	Liver function damage and immune dysfunction
Vasodialatators			
Minoxidil[a]	5–100	1	Hypertrichosis
Hydralazine[b]	25–100	2	Lupus syndrome
Renin inhibitors			
Aliskiren	150–300	1	Diarrhea and hyperkalemia

[a]Listed in Europe and the United States, unlisted in China; [b]Approved in China. ACEI: angiotensin converting enzyme inhibitor; ARB: angiotensin receptor blockers; CCB: calcium channel blockers.

(1) CCB: CCB mainly plays a role in dilating blood vessels and lowering BP by blocking calcium channel on vascular smooth muscle cells, which includes dihydropyridine CCB and non-dihydropyridine CCB. In previous large-scale clinical trials of antihypertensive therapy, dihydropyridine CCB was commonly used as the research agent, and it was confirmed that the antihypertensive treatment based on dihydropyridine CCB can significantly reduce the risk of stroke in hypertensives.[145–148] Dihydropyridine CCB can be combined with other four classes of drugs, especially suitable for patients with elderly hypertension, isolated systolic hypertension, stable angina pectoris, coronary or carotid atherosclerosis or peripheral vascular disease.[149] Common side effects include reflex sympathetic activation leading to rapid heartbeat, facial flushing, edema of the ankle, hyperplasia of the gums, and so on. Dihydropyridine CCB has no

Table 9. Single-pill combination preparations.

Major components and single dose	Tablets per day	Times per day	Major side effects
Losartan pot./Hydrochlorothiazide			Angioneurotic edema (occasional) and abnormal serum potassium
50 mg/12.5 mg	1	1	
100 mg/12.5 mg	1	1	
100 mg/25 mg	1	1	
Valsartan/Hydrochlorothiazide 80 mg/12.5 mg	1–2	1	Angioneurotic edema (occasional) and abnormal serum potassium
Irbesartan/Hydrochlorothiazide 150 mg/12.5 mg	1	1	Angioneurotic edema (occasional) and abnormal serum potassium
Telmisartan/Hydrochlorothiazide 40 mg/12.5 mg	1	1	Angioneurotic edema (occasional) and abnormal serum potassium
80 mg/12.5 mg	1	1	
Olmesartan/Hydrochlorothiazide 20 mg/12.5 mg	1	1	Angioneurotic edema (occasional) and abnormal serum potassium
Captopril/Hydrochlorothiazide 10 mg/6 mg	1–2	1–2	Coughing, angioneurotic edema (occasional), abnormal serum potassium
Lisinopril/Hydrochlorothiazide 10 mg/12.5 mg	1	1	Coughing, angioneurotic edema (occasional), abnormal serum potassium
Enalapril/Hydrochlorothiazide 5 mg/12.5 mg	1	1	Coughing, angioneurotic edema (occasional), abnormal serum potassium
Benazepril/Hydrochlorothiazide 10 mg/12.5 mg	1	1	Coughing, angioneurotic edema (occasional), abnormal serum potassium
Perindopril/Indapamide 4 mg/1.25 mg	1	1	Coughing, angioneurotic edema (occasional), abnormal serum potassium
Perindopril/Amlodipine 10 mg/ 5 mg	1	1	Dizziness, headache and coughing
Amlodipine/Valsartan 5 mg/80 mg	1	1	Headache, ankle edema, angioneurotic edema (occasional)
Amlodipine/Telmisartan 5 mg/80 mg	1	1	Headache, ankle edema, angioneurotic edema (occasional)
Amlodipine/Benazepril 5 mg/10 mg	1	1	Headache, ankle edema, angioneurotic edema (occasional)
2.5 mg/10 mg	1	1	Headache, ankle edema, angioneurotic edema (occasional)
Amiloride/Hydrochlorothiazide 2.5 mg/25 mg	1	1	Abnormal serum potassium, hyperuricemia
Nitrendipine/Atenolol 10 mg/20 mg	1	1–2	Headache, ankle edema, bronchospasm, bradycardia
5 mg/10 mg	1–2	1–2	
Reserpine/Hydrochlorothiazide/ Dihydralazine/Promethazine 0.032 mg/3.1 mg/4.2 mg/2.1 mg	1–3	2–3	Digestive ulceration and sleepiness
Reserpine/Triamterene Hydrochlorothiazide/Dihydralazine 0.1 mg/12.5 mg/12.5 mg/12.5 mg	1–2	1	Digestive and headache
Zhenjujiangya tablet Clonidipine 0.03 mg/ Hydrochlorothiazide 5 mg	1–3	2–3	Hypotension and abnormal serum potassium
Enalapril/Folic acid 10 mg/0.8 mg	1–2	1–2	Coughing, nausea, angioneurotic edema (occasional), headache, ankle edema, myalgia
Amiloride/Atorvastatin 5 mg/10 mg	1	1	Transaminase elevation
Candesartan/Hydrochlorothiazide 16 mg/12.5 mg	1	1	Upper respiratory tract infection, backache, abnormal serum potassium

For details on the usage of antihypertensive drugs, please refer to the relevant manual approved by China Food and Drug Administration.

Table 10. Strong indications for antihypertensive drugs commonly available.

Indication	CCB	ACEI	ARB	diuretic	β-blockers
Left ventricular hypertrophy	+	+	+	±	±
Stable CAD	+	+[a]	+[a]	–	+
Post-myocardial infarction	–[b]	+	+	+[c]	+
Heart failure	–[e]	+	+	+	+
Prevention of atrial fibrillation	–	+	+	–	–
Cerebrovascular disease	+	+	+	+	±
Carotid intima-media thickness	+	±	±	–	–
Proteinuria/Microproteinuria	–	+	+	–	–
Renal inadequacy	±	+	+	+[d]	–
Elderly hypertension	+	+	+	+	±
Diabetes	±	+	+	±	–
Dyslipidemia	±	+	+	–	–

[a]Secondary prevention of CHD; [b]long-acting CCB can be used to control hypertension in patients with a history of myocardial infarction; [c]spironolactone; [d]loop diuretics should be chosen when eGFR < 30 mL/min; [e]amlodipine and felodipine are also applicable. +: applicable; −: insufficient evidence or not applicable; ±: likely applicable. ACEI: angiotensin converting enzyme inhibitor; ARB: angiotensin receptor blockers; CCB: calcium channel blockers; eGFR: estimated glomerular filtration rate.

absolute contraindications, but patients with tachycardia and heart failure should be used with caution. Nifedipine is generally not recommended for patients with acute coronary syndrome, because it is a short-acting agent.

Non-dihydropyridine CCB commonly used in clinic is also applicable for antihypertensive therapy, the major side effects include inhibition of cardiac systolic function and conduction function, second to third degree atrioventricular block. Heart failure patients are forbidden to use it. Gingival hyperplasia induced by non-dihydropyridine CCB sometimes occurs. Therefore, doctors should detail patients' medical history and perform electrocardiogram before prescription, and the patients should be reviewed within two to six weeks after using it.

(2) ACEI: the mechanism of ACEI is to inhibit angiotensin-converting enzyme, block the production of renin angiotensin II and the degradation of kininase, so it can play a role in antihypertensive treatment.Various large-scale clinical trials conducted in hypertensive patients of the United States and Europe have demonstrated their good protection on target organ and preventive effects in cardiovascular end points.[68,150,151] ACEI has a definite antihypertensive effect and has no adverse effects on glycolipid metabolism. Salt restriction diet or combination with diuretic can increase its antihypertensive effect. ACEI is especially suitable for patients with chronic heart failure, cardiac insufficiency after myocardial infarction, prevention of atrial fibrillation, diabetic nephropathy, non-diabetic nephropathy, metabolic syndrome, proteinuria or microalbuminuria. The most commonly seen side effect is dry cough which often occurs in the early stage of medication. Patients with mild symptoms can keep taking the drug, while those who cannot tolerate should switch to ARB. Other side effects include hypotension, rash, occasional angioedema and dysgeusia. Long-term administration may lead to hyperkalemia; therefore, serum potassium and creatinine levels should be monitored regularly. Patients with bilateral renal artery stenosis, hyperkalemia, and pregnancy are the contraindication of ACEI.

(3) ARB: ARB plays its antihypertension role by the mechanism of blocking angiotensin II type 1 receptor. Kinds of large-scale clinical trials conducted in hypertensives of the United States and Europe have demonstrated that ARB can reduce the incidence of cardiovascular complications[152] and risk of cardiovascular events[153] in patients with a history of cardiovascular disease (such as CHD, stroke or peripheral arterial disease). ARB can also reduce the level of proteinuria and microalbuminuria in patients with diabetes or kidney disease.[113,115] It is especially suitable for patients with left ventricular hypertrophy, heart failure, diabetic nephropathy, CHD, metabolic syndrome, microalbuminuria/proteinuria, or patients who cannot tolerate ACEI. There is also a prevention effect of atrial fibrillation in ARB.[154,155] This class of drugs has less side effects and only occasionally observed diarrhea. Long-term administration may lead to hyperkalemia, so it should be paid attention to monitoring changes of serum potassium and creatinine. ARB is forbidden used by pregnant women and patients with bilateral renal artery stenosis or hyperkalemia.

(4) Diuretics: diuretics mainly exert their antihypertensive effect by urinating sodium and reducing the capacity load. The diuretics used to control BP are mainly thiazide

Table 11. Selection of principal anti-hypertensive drugs for clinical practice.

Classes	Indications	Contraindications	
		Absolute	Relative
Dihydropyridines CCB	Elderly hypertension<br>Peripheral vascular disease<br>Isolated systolic hypertension<br>Stable angina pectoris<br>Carotid atherosclerosis<br>Coronary atherosclerosis		Tachydysrhythmia<br>Heart failure
Non-dihydropyridines CCB	Angina pectoris<br>Carotid atherosclerosis<br>Supraventricular tachycardia	A-V block (grade 2 or 3)<br>Heart failure	
ACEI	Heart failure<br>Coronary heart disease<br>Left ventricular hypertrophy<br>Left ventricular dysfunction<br>Prevention of atrial fibrillation<br>Carotid atherosclerosis<br>Non-diabetic nephropathy<br>Diabetic nephropathy<br>Proteinuria/Microproteinuria<br>Metabolic syndrome	Pregnancy<br>Hyperkalemia<br>Bilateral reno-arterial stenosis	
ARB	Diabetic nephropathy<br>Proteinuria/Microproteinuria<br>Coronary heart disease<br>Heart failure<br>Left ventricular hypertrophy<br>Prevention of atrial fibrillation<br>ACE-inhibitor coughing<br>Metabolic syndrome	Pregnancy<br>Hyperkalemia<br>Bilateral reno-arterial stenosis	
Diuretics (thiazides)	Heart failure<br>Elderly Hypertension<br>Old-aged Hypertension<br>Isolated systolic hypertension	Gout	Pregnancy
Diuretics (loop diuretics)	Renal insufficiency<br>Heart failure		
Diuretics (anti-aldosterone)	Heart failure<br>Post-myocardial infarction	Renal failure<br>Hyperkalemia	
β-blockers	Angina pectoris<br>Post-myocardial infarction<br>Tachydysrhythmia<br>Chronic heart failure	A-V block (grade 2 or 3)<br>asthma	COPD<br>Peripheral vascular disease<br>glucose intolerance<br>Athletes
α-blockers	Prostatic hyperplasia<br>Hyperlipidemia	Orthostatic hypotension	Heart failure

ACEI: angiotensin converting enzyme inhibitor; ARB: angiotensin receptor blockers; CCB: calcium channel blockers; COPD: chronic obstructive pulmonary disease.

diuretics, which are divided into thiazide diuretics and thiazide-like diuretics. The former includes hydrochlorothiazide, benzfluorothiazide, *etc*., while the latter includes chlorthalidone, indapamide and so on. The PATS[130] study confirmed that indapamide treatment can significantly reduce the risk of stroke recurrence. Low-dose thiazide diuretics (such as hydrochlorothiazide 6.25–25 mg) have little effect on metabolism, and can significantly increase antihypertensive effect of the latter agent when combined with other antihypertensive drugs (especially ACEI or ARB). This class of drugs is especially suitable for elderly hypertension, isolated systolic hypertension and heart failure.

They are also one of the basic drugs for treating refractory hypertension.[156] The side effects of diuretics are closely related to the dosage, so small doses should generally be prescribed. Thiazide diuretics can lead to hypokalemia, long-term users should regularly monitor serum potassium, and supply potassium salt appropriately. Patients with gout are forbidden to use. Patients with hyperuricemia and obvious renal insufficiency should be used with caution. If the latter requires diuretics, loop diuretics such as furosemide are preferred.

Potassium-sparing diuretics such as amiloride, aldosterone receptor antagonists such as spironolactone, *etc*., are also applicable for the control of refractory hypertension. They do not increase potassium excretion while urinating sodium. When combined with other antihypertensive drugs with potassium-sparing effect such as ACEI or ARB, it should be aware of the risk of hyperkalemia. Long-term administration of spironolactone may lead to some side effects such as gynecomastia.

(5) β-blockers: β-blockers play a role in antihypertensive treatment mainly by inhibiting over-activated sympathetic nerve activity and myocardial contractility, plus heart rate slowing. Highly selective β1 blockers have a high affinity for β1 receptor, thus fewer side effects are induced by blocking the β2 receptor, which not only can reduce BP, but also can protect target organs and reduce the risk of cardiovascular events.[157,158] β-blockers are especially suitable for patients with tachyarrhythmia, CHD, chronic heart failure,[159,160] increased sympathetic activation, and high-dynamic hypertension. β-blockers' side effect commonly include fatigue, cold limbs, agitation, gastrointestinal upset, *etc*., and it may also affect glucose and lipid metabolism. Patients with second/third degree atrioventricular block and asthma are banned to use. Athletes and patients with chronic obstructive pulmonary disease, peripheral vascular disease or abnormal glucose tolerance should be used with caution. β-blockers are generally not preferred when glucose and lipid metabolism is abnormal, if necessary, highly selective β-blockers can also be used with caution. Long-term users of β-blockers can exhibit rebound phenomenon when suddenly withdrawal, which means the original symptom is worsen or new performance (such as BP rebound, headache, anxiety, *etc*.) is appeared. That is so called "withdrawal syndrome".

(6) α-blockers: α-blockers are not the first choice for the antihypertension treatment, and are mainly used for patients with hypertension and benign prostatic hyperplasia. It is also suitable for the treatment of refractory hypertension.[161] Initial medication should be administered before bedtime in order to prevent postural hypotension. The sitting and standing BP should be monitored during the application and the controlled release formulation is preferred. Patients with postural hypotension are banned to use while those with heart failure can be used with caution.

(7) Renin inhibitors: the mechanism of renin inhibitors is a direct inhibition of renin, to reduce the production of angiotensin II, which can significantly reduce BP levels in hypertensive patients.[162–165] Other effects of renin inhibitors may also be helpful in reducing BP and protecting tissues, such as lowering plasma renin activity, blocking renin/renin receptors, and reducing production of intracellular angiotensin II. These drugs are well tolerated. Rash and diarrhea are their most common side effects.

5.4.4 Combination therapy of antihypertensive drugs

Combination therapy has become the basic method to treat hypertension.[166] In most cases, it requires two or more drugs to have BP controlled below the target BP.

(1) Indications for combination therapy: high-risk group of patients with BP ≥ 160/100 mmHg or 20/10 mmHg higher than that of the target BP often require two drugs for initial treatment. It is also feasible to initiate with small dose combination therapy if the patient's BP exceeds 140/90 mmHg. If the target BP is still not achieved, the dosage may be increased on the basis of the original medication, sometimes three or more than three drugs may be needed for antihypertension treatment. CHIEF study shows that initial combination therapy has a good antihypertensive effect on the middle-aged and elderly patients with moderate or high risk of cardiovascular disease in Chinese population, and significantly improves BP control rate.[120]

(2) Method of combination therapy: when the two drugs are combined, their antihypertensive mechanism should be complementary; meanwhile, they should have an additive antihypertensive effect and can offset or alleviate the side effects of each other. For example, the addition of a small dose of thiazide diuretic to the medication of ACEI or ARB can reach or exceed the antihypertensive effect which doubles the original ACEI or ARB did. Equally, the addition of dihydropyridine CCB has a similar effect of that.

(3) The regimen of combination therapy (Figure 3).

a. ACEI/ARB+thiazide diuretic: ACEI and ARB can increase serum potassium slightly, and can antagonize side effects such as hypokalemia caused by long-term administration of thiazide diuretics. The combination of ACEI or ARB plus thiazide diuretics has a synergistic effect, which is beneficial to improve the antihypertensive effect.

b. Dihydropyridine CCB+ACEI/ARB: CCB has the function of directly dilating the arteries, while ACEI or ARB can dilate both the arteries and the veins, thus the

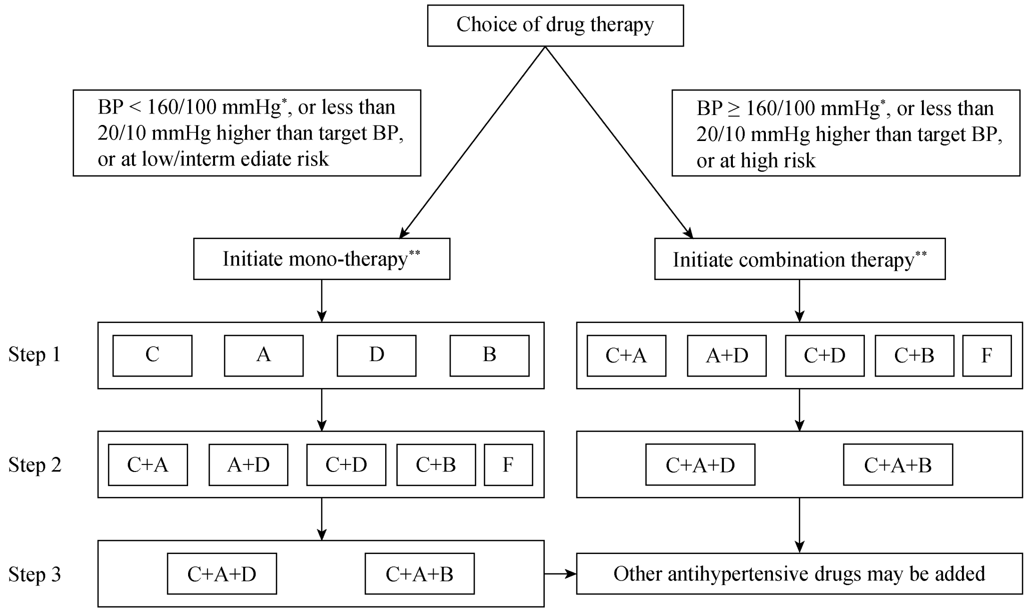

Figure 3. Flowchart for the choice of initial single versus combination antihypertensive therapy. A: ACEI or ARB; B: β-blockers; C: dihydropyridines CCB; D: thiazide-type diuretics; F: fixed-dose combination drugs. *For those with BP ≥ 140/90 mmHg and at high risk, initial low-dose combination therapy can also be recommended; **Including dosage titration and sequential addition of other agents to achieve BP target. ACEI: angiotensin converting enzyme inhibitor; ARB: angiotensin receptor blockers; BP: blood pressure; CCB: calcium channel blockers.

combination of the two drugs has a synergistic antihypertensive effect. A common side effect that dihydropyridine CCB has is ankle edema, which can be alleviated or offset by ACEI or ARB. The CHIEF study showed that low-dose long-acting dihydropyridine CCB preparation plus ARB used in hypertensive patients newly diagnosed can significantly improve the control rate of BP.[122] In addition, ACEI or ARB can also partially block the adverse reactions induced by CCB to increase patients' reflex sympathetic tone and heart rate.

c. Dihydropyridine CCB+Thiazide diuretic: the FEVER study[40] has confirmed that combination therapy of dihydropyridine CCB plus thiazide diuretic can reduce the risk of stroke in hypertensive patients.

d. Dihydropyridine CCB+β-blockers: CCB has the effect of dilating blood vessels and slightly increasing heart rate, which can offset the vasoconstriction and reduction of heart rate induced by β-blockers. The combination of the two drugs can alleviate their side effects.

The combination therapy below is optimized and recommended in China: dihydropyridine CCB+ARB; dihydropyridine CCB+ACEI; ARB+thiazide diuretic; ACEI+ thiazide diuretic; dihydropyridine CCB+thiazide diuretic; dihydropyridine CCB+β-blockers.

The combination therapy below can be taken into consideration: thiazide diuretic+β-blockers; α-blockers+β-blockers; dihydropyridine CCB+potassium-sparing diuretics; Thiazide diuretic+Potassium-sparing diuretics.

The combination therapy below that is not regularly recommended but can be used with caution if necessary: ACEI+β-blockers; ARB+β-blockers; ACEI+ARB; centrally acting agents+β-blockers.

The combination of multiple drugs: combination therapy of three drugs, the medication based on the combination of two drugs above plus another antihypertensive drug. One of the most commonly used combination is dihydropyridine CCB+ACEI (or ARB) + thiazide diuretics. Combination therapy of four drugs: on the basis of combination three drugs above, the fourth drug (such as β-blockers, aldosterone receptor antagonists, triamterene, Clonidine or α-blockers, *etc*.) is added. This medication is mainly for patients with refractory hypertension.

(4) Single-pill combination (SPC): SPC is a group of agents commonly used for antihypertension treatment, usually composed of two or more antihypertensive drugs with different mechanism.[167] Compared with the freely combined use of various drugs, the advantage of it is convenient to use and may help improve curative effect patient compliance, which is the new trend of combination therapy. It should be paid attention to the contraindications or possible side effects of their corresponding components when SPC is administrated.

Traditional combination preparations in China, such as "Fu-fang-jiang-ya-pian (Composite hypotensor)", "Fu-fang-li-xue-ping-an-ben-die-ding-pian", "Zhen-ju-jiang-ya-yin" and so on, included reserpine, hydralazine, and hydrochlorothiazide as the main components wildly used at that time. These compound preparations are still wildly used in grass-root clinical practice, long-acting "Fu-fang-li-xue-ping-an-ben-die-ding-pian" is one of the representatives.

New single-pill combinations are generally composed of two drugs with different mechanisms. Most of them are taken orally once per day, which is convenient to use and can improve patient compliance. These agents available in China mainly include ACEI plus thiazide diuretics, ARB plus thiazide diuretics; dihydropyridine CCB plus ARB, dihydropyridine CCB plus ACEI, dihydropyridine CCB plus β-blockers, thiazide diuretics plus potassium-sparing diuretics and so on.

5.5 Advance in device-based hypertension treatment

Key point 5E

- Renal denervation (RDN) is not recommended for the routine treatment of resistant hypertension outside of the framework of clinical trials until enough evidence regarding safety and efficacy of such therapy becomes available.

RDN is a novel BP-lowering technique. Although the result from SYMPLICITY HTN-3 study is negative, the technique should not be denied. The study raised many issues, such as screening criteria for appropriate patient, operating skill and improvement of RDN device, *etc*., which all should be considered in clinical research.[168] In recent years, novel RDN devices are emerging, which will be hopeful to achieve more effective renal denervation.[169] Interim data from two recent RCTs (SPYRAL HTN-OFF MED and SPYRAL HTN-ON MED) with a sham procedure control showed that renal denervation is effective and safe in lowing BP in patients with grade I –II hypertension.[170,171] Further RCT studies are needed before RDN can be recommended for the routine treatment of hypertension outside of the framework of clinical trials.

Other device-based therapies for hypertension, such as carotid baroreceptor stimulation,[172] central iliac arteriovenous anastomosis,[173] carotid body ablation, deep brain stimulation and device-guided breathing exercise are all undergoing early stages of investigation, whose safety and efficacy are unclear. So far, we do not know which approach in them will be accepted in clinical practice in the future.

5.6 Treatment of associated risk factors

5.6.1 Lipid treatment

Key point 5F

- Patients with hypertension and dyslipidemia should be actively treated with antihypertensive therapy and moderate lipid-lowering therapy based on changes in therapeutic lifestyle.
- For patients with low and intermediate risk of atherothrombotic cardiovascular disease (ASCVD), when the blood lipid level cannot reach the target value after strict implementation of lifestyle intervention for six months, drug lipid-lowering therapy should be considered.
- For patients with hypertension at risk of ASCVD, statin therapy should be initiated immediately. Moderate-strength statins (IA) can be used, if cholesterol-lowering drugs can be combined with when necessary.[173]

Hypertension and dyslipidemia are important risk factors for atherosclerotic cardiovascular and cerebrovascular diseases. Hypertension associated with dyslipidemia significantly increases the risk of cardiovascular events. The Guidelines for the Prevention and Treatment of Dyslipidemia in Chinese Adults (Revised 2016)[174] for the first time clarified that the ideal cholesterol level in the primary prevention population of Chinese ASCVD is LDL-C < 2.6 mmol/L (non-HDL-C < 3.4 mmol/L).

A large number of randomized controlled clinical trials (including China's completed CCSPS study[174-182]) have shown that statin lipid-lowering therapy can significantly reduce the risk of all-cause mortality and cardiovascular events in patients with hypertension and dyslipidemia. It suggests low-medium-strength statins are safe and effective for primary prevention in patients with hypertension and dyslipidemia. However, as a primary prevention strategy for cardiovascular events, not all hypertensive patients require statin therapy. Analysis of existing data shows that low- and medium-intensity statin therapy can significantly reduce cardiovascular risk in patients with intermediate or high-risk cardiovascular disease including hypertensive patient.[183,184] The therapy is safe and tolerable.

Hypertensive patients should consider applying statins under the following conditions: hypertension with more than one metabolic risk factor or with target organ damage. Statins should be used as primary prevention for cardiovascular disease. Hypertension with clinical disease (including heart, brain, kidney, blood vessels, *etc.*) should use statins as secondary prevention. In patients with hypertension, statins can be used as primary prevention. Low-intensity statins can be used. If multiple risk factors are combined (≥ 3) or target

organ damage is severe, moderate-intensity statins can be used. Hypertensive patients use statins as secondary prevention. They can choose a moderate-intensity statin for initial treatment and high-intensity statins or statins in combination with other lipid-lowering drugs (specific intestinal cholesterol absorption inhibitors) if necessary.

In patients with hypertension and dyslipidemia, lipid-lowering therapy is treated according to the Guidelines for Prevention and Treatment of Dyslipidemia in Chinese Adults (2016 Revision).

5.6.2 Antiplatelet therapy

Key point 5G

· Antiplatelet therapy is recommended for patients with hypertension associated with ischemic cardiovascular and cerebrovascular disease (I A).[185,186]

The role of anti-platelet therapy in secondary prevention of cardiovascular and cerebrovascular diseases has been confirmed by a large number of clinical trials, which can effectively reduce the risk of cardiovascular events by 19%–25%, including 1/3 of nonfatal myocardial infarction, 1/4 of nonfatal stroke, and 1/6 of fatal vascular events.[187,188]

The following hypertensive patients should be actively treated with antiplatelet therapy: (1) patients of hypertension complicated with ASCVD should be treated with low-dose aspirin (ASA) (100 mg/day) for long-term secondary prevention;[185,186] and (2) in the case of acute episodes of thrombosis, such as acute coronary syndrome, ischemic stroke, transient ischemia, or occlusive peripheral atherosclerosis, ASA should be used in combination with a P2Y12 receptor inhibitor according to relevant guidelines. P2Y12 receptor inhibitor includes clopidogrel and ticagrelor, usually administered in the acute phase at a loading dose (ASA: 300 mg, clopidogrel: 300–600 mg or ticagrelor 180 mg), ASA (100 mg/day) was combined with clopidogrel (75 mg/d) or ticagrelor 180 mg/day for 3 to 12 months, followed by low-dose ASA (100 mg/day) for long-term secondary prevention.

The benefits of antiplatelet therapy on primary prevention of cardiovascular and cerebrovascular diseases are mainly reflected in high-risk groups, such as hypertension with diabetes, hypertension with chronic kidney disease, people aged 50–69 years with high cardiovascular risk (10-year total cardiovascular risk ≥ 10% or hypertension with three or more risk factors), so primary prevention can be carried out with small dose ASA (75–150 mg/day).[189–192] Patients with ASA intolerance can take clopidogrel (75 mg/day) as a replacement. Long-term use of ASA in patients with hypertension should be noted:[193,194] (1) it should be taken after BP well controlled (< 150/90 mmHg). ASA may increase the risk of cerebral hemorrhage in uncontrolled hypertensive patients. (2) Aspirin enteric-coated tablets are recommended on an empty stomach to reduce gastrointestinal reactions. (3) Among patients with high risk factor of gastrointestinal bleeding, such as digestive tract disease (ulcer disease and its complications), more than 65-years-old, taking corticosteroids, anticoagulants or non steroidal anti-inflammatory drugs, preventive measures should be taken, including screening and treatment of helicobacter pylori infection, the prophylactic use of proton pump inhibitors, and adopting the reasonable scheme of combination of antithrombotic drugs. (4) Patients with active gastric ulcer, severe liver disease, renal failure, hemorrhagic disease should taking ASA with caution or stopping taking. (5) Patients with severe gastrointestinal bleeding after taking ASA should stop taking, and treatment should be conducted according to the related path of hemorrhage, and proton pump inhibitor treatment should be added for mild cases.

5.6.3 Blood glucose control

Key point 5H

· Target of blood glucose control: HbA1c < 7%; fasting blood glucose (FBG) 4.4–7.0 mmol/L; two hours postprandial blood glucose or high peak blood glucose < 10.0 mmol/L. Target of blood glucose control could be more relaxed for patients prone to hypoglycemia, with long course of disease, elder, with comorbidity or various complications.

It's common for hypertensive patients to have hyperglycemia, who are often combined with a variety of other metabolic cardiovascular risk factors, such as obesity, dyslipidemia, fatty liver, proteinuria, hyperuricemia, *etc*., which would promote and aggravate the pathogenesis and development of cardiovascular risk. Therefore, it requires comprehensive control of multiple factors including healthy lifestyles and drugs against various metabolic cardiovascular risks, to achieve blood glucose control.

Target of blood glucose control: HbA1c < 7%; FBG 4.4–7.0 mmol/L; two hours postprandial blood glucose or high peak blood glucose < 10.0 mmol/L. Target of blood glucose control could be more relaxed for patients prone to hypoglycemia, with long course of disease, elder, with comorbidity or various complications, which also applies to patients with type 1 diabetes mellitus complicated with kidney disease and fundus disease. The basic principle is no hypoglycemia or hyperglycemia occurrence.

The principle of dietary adjustment: control total calorie, and carbohydrates should account for 55% to 65% of total calories; protein should be no more than 15% of total calories. Try best to control the body weight within the normal range. Eat little and often with the total calories being the same.

Principles of exercise and activity: the exercise and activity should be moderate, regular and individualized. It is recommended to take skeletal muscle isotonic exercises, such as walking, swimming, *etc*., whereas heavy sports and heavy exercise should be restricted. Hours should be regular for patients receiving insulin therapy, for example, meals and exercise should be regular with a fixed quantity.

Main principles of drug treatment:[189,195]

(1) For most patients with type 2 diabetes mellitus, metformin is preferred.

(2) Light-weight petients and patients whose blood sugar cannot be effectively controlled with metformin alone should switch to or add sulfonylurea or glinide-type hypoglycemic agent or dipeptidyl peptidase-4 inhibitor, α-glycoside enzyme inhibitor.

(3) The novel sodium-glucose cotransporter 2 (SGLT2) inhibitor or glucagon-like peptide-1 (GLP-1) receptor agonist, could reduce blood glucose effectively as well as reduce SBP and weight slightly. It was shown in recent clinical trials that the SGLT2 drugs, empagliflozin and canagliflozin and the GLP-1 receptor agonist, liraglutide, can reduce cardiovascular mortality, and dapagliflozin can reduce the risk of cardiovascular death or hospitalization for heart failure.[196–198]

(4) If it is difficult to control blood glucose after two kinds of medium-dose hypoglycemic drugs have been used, hypoglycemic agents can be administrated orally during daytime, and intermediate-acting or super-long-acting insulin treatment can be injected before bedtime; if blood glucose is still not controlled effectively, multiple insulin injections can be adopted everyday.

(5) For new-onset diabetes with fasting blood glucose above 11 mmol/L or HbA1c over 9%, short-term intensive insulin therapy could be adopted so as to control blood glucose and preserve islet β-cell function as soon as possible.

(6) In the process of antihypertensive treatment, the effect of antihypertensive drugs on glycemic control should be taken into consideration. For instance, long-term application of thiazide diuretics with large doses could result in blood glucose elevating; β−recepter blockers may mask hypoglycemia responses including heart rate increase, *etc*..

For detailed guidance on diabetes prevention and treatment, or precautions for insulin application, see China Guidelines for Diabetes Prevention and Treatment (Edition 2017).[199]

5.6.4 Treatment of hypertension complicated with atrial fibrillation (AF)

Key point 5I

· In hypertensive patients with risk of atrial fibrillation (such as left atrial enlargement, left ventricular hypertrophy or reduced cardiac function), it is recommended to use renin-angiotensin aldosterone system (RAS) inhibition drugs (especially ARB) to reduce the occurrence of atrial fibrillation (IIA, B).[200]

· Patients with atrial fibrillation and risk factors for thromboembolism should be treated with anticoagulant therapy in accordance with the current guidelines (I, A).[201]

Hypertension is an important risk factor for atrial fibrillation.[202] In hypertensive patients with risk of atrial fibrillation, such as left atrial enlargement, left ventricular hypertrophy, or reduced cardiac function, It is recommended to use RAS inhibition drugs (especially ARB) to reduce the occurrence of atrial fibrillation.[200] A common important complication of hypertension and AF is stroke. Hypertension is one of the risk factors for stroke and systemic embolism in non-valvular AF patients. Uncontrolled hypertension is also a risk factor for bleeding in patients with atrial fibrillation. All hypertensive patients with non-valvular AF should be assessed for risk of thromboembolism based on $CHADS_2$ or CHA_2DS_2-VASc scores and risk assessment of bleeding.

Anticoagulant therapy should be given in accordance with the current guidelines for patients with hypertension complicated with AF who have risk factors for thromboembolism.[201] Oral anticoagulant warfarin can be used under the guidance of the international standardized ratio (INR) to control the INR in 2.0–3.0. Because of the metabolic gene characteristics of warfarin metabolism in Chinese population, special consideration and attention should be given in the dose of initial or adjustment of warfarin treatment in order to ensure the efficacy and avoid bleeding side effect. The novel oral anticoagulants were compared with warfarin in clinical trials in patients with non-valvular AF with the results of non-inferior or superior efficacy in stroke and systemic embolism prevention with no more than or less bleeding complications than warfarin,[203–206] and all drugs significantly reduced intracranial hemorrhage. It is recommended to follow the recommendations in indication and contraindication in relevant guidelines to use and follow up appropriately. Rate or rhythm control should be given in patients with symptomatic atrial fibrillation in accordance with the current guidelines.[201]

It is recommended to use the average value of three BP measurements for the potential measurement error by the irregular rhythm in AF. If possible an electronic sphygmomanometer with the function of detecting atrial fibrillation can be used.

5.6.5 Management of hypertension with multiple risk factors

Key point 5J

- Lifestyle intervention is the basis of cardiovascular disease prevention in hypertensive patients with multiple risk factors. It is suggested that hypertensive patients with elevated homocysteine level should be supplemented with fresh vegetables, fruits and folic acid (IIa, B),[41,137] if necessary.
- Lifestyle intervention is the basis of cardiovascular disease prevention in hypertensive patients with multiple risk factors. The management of hypertensive patients is an important approach for intervention of the multiple risk factors.

Studies showed that intervention of multiple risk factors with health education, lifestyle intervention and drug treatment may significantly improve the individual risk factor control[207–210] and risk factor aggregation status[211] of high-risk population of cardiovascular disease in community. Lifestyle interventions are detailed in 5.3. The fixed-proportion combination of antihypertensive drugs and other cardiovascular drugs includes dihydropyridine CCB+statin, *etc*. The use of such drugs should be based on the risk factors and concurrent clinical diseases of the patient, the indications and contraindications of antihypertensive drugs and non-antihypertensive drugs should be grasped.

Treatment of hypertension with elevation in homocysteine (HCY) level: nutrition intervention study in Lin County[137] and CSPPT study[41] suggest supplement of folic acid could reduce the risk of first stroke. Appropriate supplement of fresh vegetables and fruits is recommended in hypertensive patients with elevation of HCY level, and the supplement of folic acid should be conducted as necessary.

5.7 Follow-up, referral and medical record of anti-hypertensive therapy

5.7.1 Purpose of follow-up

The purpose of follow-up are to assess the treatment response, to investigate the patient's tolerance to the drug, to analyze whether or not the BP treat to target stably and the status of other risk factors, and to establish the good relationship of reciprocal trust between physician and patient.

5.7.2 Contents of follow-up

During the follow-up, office BP measurement and/or ambulatory BP monitoring are recommended, the numerical value of BP and the status of BP targeting are mastered and the adherence to drug therapy are inquired. According to the fluctuation of BP and the adverse effect of drug, the drug of hypertensive treatment will be modified. Order the patient to take medicine on time, direct the patient to improve the lifestyle and persistent long-term treatment, not to stop taking drugs at will.

5.7.3 Interval of follow-up

The interval of follow-up will be determined by total cardiovascular risk along with BP level. Patients with high–normal BP or grade 1 hypertension, with low- or moderate-risk, or taking only one kind of anti-hypertensive agent, should be visited for follow-up every two to three months. The interval of follow-up of patients with new-defined high-risk and complicated case should be shortened, and for high-risk patients whose BP not at goal or with clinical symptoms, a shorter interval (2–4 weeks) should be considered. The patient with targeting BP stably should be follow-up every month or a longer interval. The patient, whose BP could not reach the target after taking at least three kinds of anti-hypertensive agents, should be transferred to hypertensive specialist clinics for further diagnosis and treatment.

5.7.4 Medical record

Every follow-up should be recorded formally, the follow-up case should be created and community-based hospital should create patient's follow-up file. At every visit, the numerical values of BP and heart rate, together with the symptom related with BP, dosage, category and adverse effect of drugs, should be recorded in the follow-up case.

6 Treatment of hypertension for special populations

6.1 Hypertension in the elderly

Key point 6A

- In the general population aged 65 to 79 years with BPs ≥ 150 mmHg systolic and 90 mm Hg diastolic, drug therapy is recommended (IA), and when BPs is ≥ 140 mmHg systolic and 90 mmHg diastolic, pharmacologic treatment should be considered (Ⅱa, B); for the elderly ≥ 80 years of age with SBP ≥ 160 mmHg, initiate the pharmacologic treatment (Ⅱa, B).[110,212]
- In the elderly population aged 65 to 79 years, initiate pharmacologic treatment to lower BP at SBP of 150 mmHg and DBP of 90 mmHg. If the treatment is well tolerated, BP can be lowered to < 140 mmHg systolic and 90 mmHg (Ⅱa, B), and for the elderly ≥ 80 years of age, BP should be lowered to < 150 mmHg systolic and 90 mmHg (Ⅱa, B).[213]

In 2012, the prevalence of hypertension among rural and urban population aged ≥ 60 years in China was 60.6% and 57.0%, respectively, and the awareness, treatment, and control rates of hypertension were 53.7%, 48.8% and 16.1%,[2] respectively. It is uniformly accepted that ≥ 65 years of age is defined as elderly hypertension, and when SBP is more than 140 mmHg, and DBP is less than 90 mmHg it is defined isolated systolic hypertension (ISH).

6.1.1 Clinical Features

(1) Increase of SBP and high pulse pressure: ISH is the most common type of elderly hypertension, accounting for 60%–80% of elderly hypertension,[214] and reaching to 80%–90% in elderly population aged 70 years and over.[215] Elevation in SBP increases the risk of stroke, CHD and end stage renal disease.

(2) BP fluctuation: number of hypertensive patients with postural BP variability and postprandial hypotension has been increasing, including orthostatic hypotension and supine hypertension. Fluctuation in BP affects treatment results and remarkably increases the risk of cardiovascular events.

(3) The incidence of abnormal circadian rhythm of BP is high: with high presence of nocturnal hypotension and hypertension as well as the high occurrence of morning hypertension.

(4) There is an increasing number of patients with white-coat hypertension and pseudohypertension.[216]

(5) It is generally accepted that hypertension commonly coexists with other chronic diseases such as CHD, heart failure, cerebrovascular disease, renal insufficiency and diabetes, *etc*. Such condition makes the treatment more difficult.

6.1.2 Lifestyle modification

For more information on lifestyle modification, please refer to 5.3 lifestyle intervention

6.1.3 Drug therapy for the elderly hypertension

(1) Research evidence: a meta-analysis showed that drug treatment could significantly reduce the risk of stroke, CHD and all-cause mortality.[217] HYVET study (≥ 80 years) showed a reduction in the rate of stroke 30%, all-cause 21%, heart failure 64% and cardiovascular events 34%, respectively.[70] The results of clinical trials in China have proved that elderly and even very elderly can benefit greatly from the antihypertensive drug treatment.[39,70,127]

(2) BP thresholds for initiating treatment: drug therapy should be instituted for the elderly aged 65 to 79 years with BPs ≥ 150 mmHg systolic and 90 mmHg diastolic, and drug therapy should be considered when BPs is ≥ 140 mmHg systolic and 90 mmHg diastolic; and it should be initiated when the elderly turn ≥ 80 years of age with SBP ≥ 160 mmHg.[110,212]

(3) Thresholds and goals for BP treatment: the primary goal of hypertension treatment is to attain and maintain SBP treatment goals. The decision to lower BP targets and to set BP treatment goals should be on an individual basis after comprehensive assessment of comorbidities and frailty among the adult group. For the elderly aged 65 to 79 years of age, first step is to lower BP to < 150/90 mmHg, and the target BP should be maintained at < 140/90 mmHg if it is tolerated, and be lowered to < 150/90 mmHg for the elderly aged 80 years or over. If pharmacologic treatment results in SBP lower than130 mm Hg and is well tolerated, the treatment for hypertension should be maintained and does not need to be adjusted. When patients have > 75% bilateral carotid stenosis, central perfusion pressure is decreased, and intensive blood-pressure lowering may increase the risk of cerebral ischemia. Antihypertensive treatment should be based on the inhibition for the avoidance of cerebral ischemia symptoms. It is however, common to lose control of BP targets.[218] The BP of frail and elderly patients on medication to lower BP, should be regularly monitored. It is also advisable not to lower the BP excessively and at a high pace.

6.1.4 Drug application

Drug selection for optimal treatment of hypertension in the elderly: current recommended drugs to be considered as initial therapy or combination therapy for BP lowering are diuretics, calcium channel blockers (CCB), angiotensin-converting-enzyme inhibitors (ACEI), and angiotensin II receptor blocking (ARB),[69,70,219–223] which should be initiated at a low dose and be increased gradually up to the maximum dose. β-blockers are usually not the first choice for the elderly hypertensive patients without commodity disease. Diuretics may reduce glucose tolerance, induce hypokalemia, hypeluricemia and dyslipidemia. Therefore, they should be used in small doses. Alpha-adrenergic receptor antagonists (α-blockers) are drugs of second line (adjuvant drugs) which can be used for patients with benign prostatic hyperplasia or refractory hypertension. When given to the very elderly patients with postural BP variations, α-blockers should be used with caution and they may cause postural hypotension.

Drug therapy for elderly patients with isolated systolic hypertension (ISH): for patients with DBP < 60 mmHg, when SBP is < 150 mmHg, no medication is recommended. If SBP is 150–179 mmHg, small dose of antihypertensive drugs can be initiated, and when SBP is ≥ 180 mmHg, anti-

hypertensive drugs should be used, but observation for adverse effects and altering BP is recommended.

6.2 Hypertension in children and adolescents

Key point 6B

- Routine blood pressure measurement should begin at three years of age; appropriate cuff size is important for accurate blood pressure measurement, and most children aged ≥ 12 years can use adult cuffs;
- The diagnosis of hypertension in children is based on blood pressure measurements on three occasions. Childhood hypertension is defined as SBP and/or DBP ≥ 95% on three occasions. A child can be diagnosed with hypertension when SBP and/or DBP reach stage 2 hypertension even one time;
- Lifestyle interventions are recommended for all children with stage 1 hypertension. Medication treatment for stage 2 hypertension begins with low-dose single medication, and treatment regimen and course should be adjusted individually.

6.2.1 Clinical features and epidemiology of hypertension

6.2.1.1 Clinical features

Primary hypertension is more common among children and adolescents < 18 years (referred to as "children"). Children with primary hypertension often are asymptomatic with only slightly increased BP. Thus, diagnosis of childhood hypertension is difficult without regular BP measurement. The proportion of primary hypertension increases with age, and hypertension that occurs during puberty is mostly primary.[224,225]

6.2.1.2 Epidemiology

Data from the Chinese National Surveys on Students' Constitution and Health in 2010 showed that the prevalence of hypertension among children was 14.5% (16.1% for boys versus 12.9% for girls).[226] Studies have shown that the prevalence after multiple separate occasions is around 4%–5%.[227,228] A variety of risk factors for childhood primary hypertension has been identified. Obesity is the most important risk factor with approximately 30%–40% of children with primary hypertension being obese.[226] Other risk factors include parental hypertension history, low birth weight, premature birth, high salt intake, sleep insufficiency, and physical inactivity.

6.2.1.3 Secondary hypertension in children

Secondary hypertension in children is usually characterized by dramatical increase in BP but sometimes characterized by mild or moderate increase. Secondary hypertension usually has definite causes, including as kidney disease, renal artery stenosis, aortic coarctation, endocrine diseases, and drugs. Renal disease is the most common cause accounting for about 80% of secondary hypertension.[229]

6.2.2 Short- or long-term impacts of childhood hypertension

Early target-organ damages have occurred among approximately 30% to 40% of hypertensive children.[230–232] Abnormal left ventricular geometry is the most common target-organ damage, and other damages include increased vascular intima-media thickness, reduced arterial elasticity, reduced renal function, and fundus arteriosclerosis.[233–235]

Childhood hypertension can continue into adulthood, and about 40% of hypertensive children still have hypertension in adulthood without intervention[236,237] Hypertensive children have significantly increased risks of cardiovascular and kidney diseases in adulthood.[238–241]

6.2.3 BP measurement and diagnostic evaluation in children

6.2.3.1 BP Measurement

The methods, operations and sphygmomanometer selection for childhood BP measurement are described in office BP section in this guideline (*i.e.*, 3.5.1).

Selecting appropriate cuff size is important for accurate BP measurement in children. Table 12 shows commonly used cuff sizes and the corresponding upper arm circumference and age. In order to be consistent with method of BP measurement in the criteria, BP at the radial artery of right upper arm should be measured in routine examinations.[242,243] For children having first BP measurement, all four limbs should be measured to exclude aortic stenosis and should be measured in different positions (sitting, lying, and standing) to detect orthostatic hypertension.

Childhood hypertension is identified based on three or more separate occasions with at least 2-week time intervals. Multiple BP measurements on the first instance can significantly reduce the rate of false positive and the burden of measurements on second occasion.[228] Three BP measurements on each occasion are recommended and the average

Table 12. Arm circumference and age for cuff size model.

Cuff size model	Arm circumference, cm	Age group, yrs
XS	12–18	3–5
S	18–22	6–11
M	22–32	≥ 12
L	32–42	-
XL	42–50	-

value of the last two readings or the lowest value is recommended to be used for diagnosis. Children ≥ 3 years old should have their BP measured and physical development evaluated annually if possible.

6.2.3.2 Childhood hypertension criteria

Given the influence of height on BP in children, we established sex-, age- and height-specific BP reference standard for Chinese children aged 3 to 17 years old (referred to Supplement Tables) based on the 2010 standard.[243] Childhood hypertension can be categorized according to the age- and height- specific 50th, 90th, 95th, and 99th percentile values in boys and girls: hypertension is classified as having SBP and/or DBP ≥ 95th percentile; high- normal BP as having SBP and/or DBP 90th–95th percentile or ≥ 120/80 mmHg. The standard has been shown to have a good predictive value for adult subclinical cardiovascular damage,[244] and can be used for childhood hypertension diagnosis in the epidemiological and clinical settings.

In order to facilitate the clinician's rapid diagnosis of hypertension in children, it is recommended to use the simplified "Formula Standard" (Table 13) when making preliminary diagnosis. The consistency rates for diagnosing hypertension between "Formula Standard" and "Table Standard" is up to 95%, and childhood hypertension defined by "Formula Standard" performs well in predicting the risk of adult hypertension and subclinical cardiovascular target-organ damage.[245,246] Children who have been diagnosed suspected hypertension by "Formula Standard" needs definite diagnosis by "Table Standard".

Table 13. Simplified formula standard for screening hypertension in Chinese children and adolescents aged 3 to 17 years.

Sex	SBP, mmHg	DBP, mmHg
Male	100 + 2 × Age	65 + Age
Female	100 + 1.5 × Age	65 + Age

The unit of age is 'years'. The formula is established based on 95th percentile values of BP from "Table Standard", which can be used for rapid screen for childhood hypertension. DBP: diastolic blood pressure; SBP: systolic blood pressure.

6.2.3.3 Diagnostic evaluation

The process of diagnostic evaluation for primary hypertension in children includes:[224] (1) evaluating BP authenticity and classifying hypertension; (2) excluding secondary hypertension; (3) evaluating degree of target-organ damage; and (4) assessing comorbidities as diabetes. Clinicians can make treatment strategy based on above evaluation results.

Repeated BP measurements on three consecutive occasions with at least two week intervals are required to make individual hypertension diagnosis. Hypertension is diagnosed when SBP and/or DBP ≥ 95th percentile for sex, age, and height on all three occasions. Childhood hypertension can be categorized into two stages: stage 1 hypertension is defined as SBP and/or DBP ranging from 95th to 99th + 5 mmHg; stage 2 hypertension is defined as SBP and/or DBP ≥ 99th + 5 mmHg (Figure 4).[224,225]

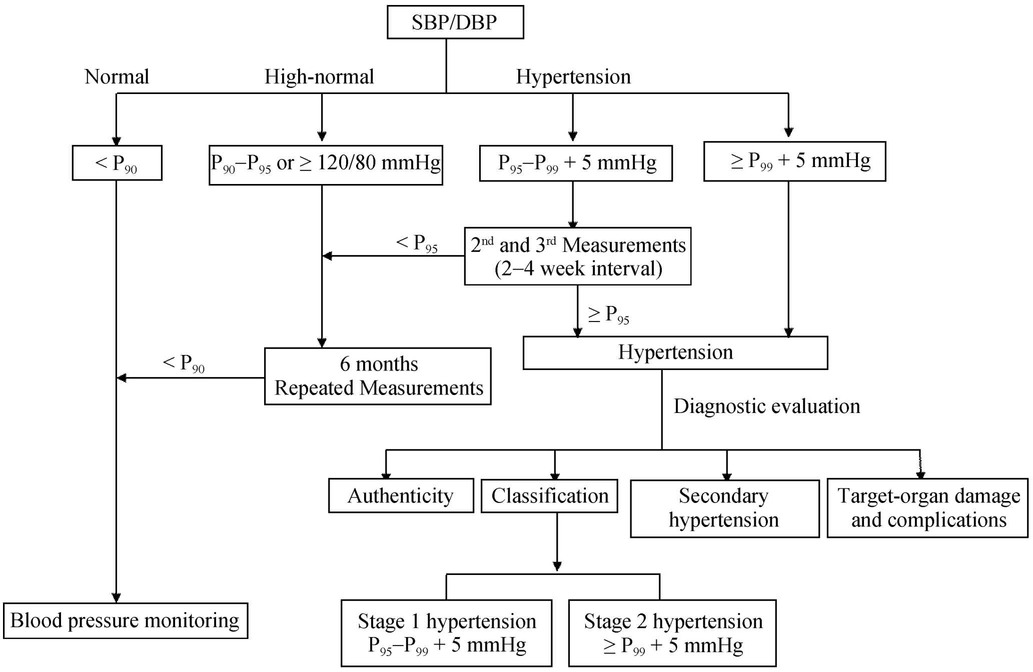

Figure 4. Flow chart for diagnostic evaluation of hypertension in children. DBP: diastolic blood pressure; SBP: systolic blood pressure.

"White-coat hypertension" and "Orthostatic hypertension" in children are common and can be identified using ambulatory BP monitoring and head-up tilt table test, respectively.

6.2.4 Treatment

6.2.4.1 Target for BP treatment

The treatment goal for children with primary hypertension should be reduction of BP to below 95th percentile. For hypertensive children combined with kidney disease, diabetes or target-organ damage, BP should be reduced to below 90th percentile to reduce target-organ damage and long-term risk of cardiovascular disease.[224]

6.2.4.2 Etiology treatment

Etiology treatment is preferred for children with secondary hypertension.

6.2.4.3 Lifestyle intervention

Children with hypertension should improve their lifestyles initially and throughout the whole treatment process: (1) obese children should control their body weight, delay the rising trend of BMI and reduce body fat without damaging normal development; (2) increase aerobic and resistance exercise and reduce sedentary time; (3) improve the dietary patterns and diversity, and limit total and fat-sourced energy; control salt and sugar intake and develop healthy dietary habits according to WHO recommendations for children; (4) avoid persistent mental stress; and (5) ensure sufficient sleep. Most hypertensive children can acquire optimal BP after lifestyle interventions.[75,247–250]

Hypertensive children should have BP monitored annually. For children with persistently high BP, ambulatory BP monitoring is recommended to identify white coat hypertension and obtain the circadian rhythm of BP.

6.2.4.4 Pharmacological therapy

Pharmacological therapy should be started in children who have stage 2 hypertension or hypertensive patients combined with one or more of the following conditions: (1) clinical symptoms; (2) diabetes; (3) secondary hypertension; and (4) target-organ damage.

Children should receive pharmacological therapy when failing to achieve target BP after lifestyle interventions for about six months and when developing stage 2 hypertension or clinical symptoms during the period of lifestyle interventions.

Pharmacologic therapy of childhood hypertension should be initiated using a low-dose and single drug and needs to be considered individually. The treatment strategy and course should be adjusted according to effect and BP levels, and combination therapy should be implemented if possible. See *Practical Pediatrics* for detailed operations.

At present, the China Food and Drug Administration (CFDA) has apporved limited antihypertensive drugs applied in children, as follows:

(1) ACEI: ACEI is one of the most commonly used antihypertensive drugs for children. Captopril is the only CFDA-approved ACEI drug.

(2) Diuretics: CFDA-approved diuretics include triamterene, chlorthalidone, hydrochlorothiazide, and furosemide.

(3) Dihydropyridine CCB: amlodipine is the only CFDA-approved dihydropyridine CCB.

(4) Adrenergic receptor blockers: CFDA-approved medications include propranolol, atenolol and prazosin.

(5) ARB: None ARB drug has been approved by CFDA.

Note: Refer to the package inserts or medication guides to confirm whether or not antihypertensive medicine is appropriate in children.

6.3 Hypertensive disorders in pregnancy

Key point 6C

- For patients with hypertension in pregnancy, the threshold for the initiation of antihypertensive drug treatment is BP ≥ 150/100 mmHg. The target of treatment is 150/100 mmHg (IIb, C).[251,252]
- The drug treatment for hypertension can be withheld until BP ≥ 160/110 mmHg if the patient has no proteinuria or other target organ damage.[253]
- In pregnant women with mild hypertension, non-pharmacologic intervention should be emphasized, with simultaneous monitoring of BP, proteinuria/target organ damage, and so on.

The prevalence of hypertensive disorders accounts for 5%–10% of pregnant women. Seventy percent of hypertensive disorders in pregnancy develop 20 weeks after gestation, while 30%, predate pregnancy.[254,255] Hypertension in pregnancy is associated with increased risk of placental abruption, cerebral hemorrhage, disseminated intravascular coagulation, acute hepatic failure, acute renal failure, and intrauterine growth retardation. It is also an important factor associated with maternal and fetal mortality.

6.3.1 Classification of hypertensive disorders in pregnancy

Hypertensive disorders in pregnancy can be divided into four categories: gestational hypertension, preeclampsia/eclampsia, chronic hypertension and chronic hypertension with superimposed preeclampsia. Gestational hypertension develops after 20 weeks of gestation without any manifestations of target organ damage and recovers within the first 12 weeks after delivery. Chronic hypertension predates pregnancy or develops within the first 20 weeks of gestation, and/or

persists after delivery. Preeclampsia is pregnant hypertension in association with proteinuria (≥ 300 mg/day) or established organ damage (heart, lung, liver or renal) but no proteinuria. Severe eclampsia is diagnosed as hypertension (≥ 160/110 mmHg) in association with clinical proteinuria and/or cerebral malfunction, blurred vision, pulmonary edema, renal failure, thrombocytopenia (platelet count < 100 × 10^9/L), hepatic failure, abnormal placental circulatory failure, etc.

6.3.2 Treatment strategy

The primary objective of antihypertensive treatment is to maintain maternal-fetal safety, support a smooth delivery and reduce perinatal mortality. The threshold for the initiation of antihypertensive drug treatment is BP ≥ 150/100 mmHg. The target of treatment is 150/100 mmHg.[251,252] The drug treatment can be withheld until BP ≥ 160/110 mmHg if the patient has no proteinuria or other target organ damage.[253,256] BP should not be lower than 130/80 mmHg, in order to avoid placental hypoperfusion.

6.3.3 Management of hypertension in pre-pregnancy women

It is highly recommended that women with chronic hypertension should be clinically evaluated before pregnancy to identify the cause of hypertension and the severity of BP elevation. The treatment includes non-pharmacological interventions, and antihypertensive drug treatment, if necessary. Some patients can have their BP reduced to less than 150/100 mmHg under non-pharmacological interventions to minimize the duration and intensity of drug treatment. Patients with uncontrolled BP (≥ 160/110 mmHg) need to be advised to avoid conception.

6.3.4 Non-pharmacologic treatment of hypertension in pregnancy

Non-pharmacological treatment of hypertension in Pregnancy includes salt restriction, regular exercise, emotional relaxing, weight controlling, adequate sleep, and so on. Salt intake can be reduced to about 6 g/day, while excessive salt restriction may lead to hypovolemia and placental hypoperfusion and should be avoided.[253]

6.3.5 The management of mild hypertension in pregnancy

Non-pharmacological treatment should be emphasized in uncomplicated mild hypertension, with BP, proteinuria, and blood cell counts closely monitored. In hypertensive patients treated with multi-drug or complicated by target organ damage, dosage and number of drugs used should be as less as possible.

Patients with mild hypertension complicated by preeclampsia, since the probability of developing eclampsia is only about 0.5%, routine use of magnesium sulfate is not recommended. However, maternal BP and proteinuria, state of the fetus need to be monitored carefully.[253]

6.3.6 The management of severe hypertension in pregnancy

The primary objective of the treatment is to reduce maternal-fetal morbidity and mortality. The persistence of treatment, the target of BP, drug selection, and the indication for pregnancy termination should be prespecified. In patients with severe eclampsia, intravenous magnesium sulfate should be administrated if no contraindication exists. Patients with very high BP (≥ 180/120 mmHg) should be treated as a hypertensive emergency.

6.3.7 Drug treatment of hypertension in pregnancy

The most commonly used oral antihypertensive drugs in pregnant women include labetalol, methyldopa, and nifedipine. Low dose thiazides can also be considered in necessary (Table 14). Angiotensin-converting enzyme inhibitors

Table 14. Commonly used oral antihypertensive drugs in pregnant women.

Name	Mode of action	Dosage	Categories**	Side effects
Methyldopa	Decreasing sympathetic tone of the brain stem	200–500 mg, 2–4 times/day	B	Depression, Extremely sedation, Hypotension
Labetalol	Blocking α- and β-receptors	50–200 mg q12h, max. 600 mg/day	C	Fetal bradycardia, Pruritus
Nifedipine	Inhibiting calcium influx of arteriolar smooth muscle cells	5–20 mg q8h or sustained release preparation 10–20 mg q12h or controlled release preparation 30–60 mg q.d.	C	Hypotension
Hydrochlorothiazide*	Diuresis and natriuresis	6.25–12.5 mg/day	B	Placenta bloodstream reduction with high dose diuretics

*Diuretics should be avoided in patients with reduced placenta bloodstream (preeclampsia or fetal growth retardation). **Pregnancy Risk Categories: A, Adequate and well-controlled studies have failed to demonstrate a risk to the fetus in the first trimester of pregnancy; B, Animal reproduction studies have failed to demonstrate a risk to the fetus, and there are no adequate and well-controlled studies in pregnant women; C, Animal reproduction studies have shown an adverse effect on the fetus, and there are no adequate and well-controlled studies in humans, but potential benefits may warrant use of the drug in pregnant women despite potential risks.

and angiotensin receptor blockers are contraindicated in pregnant as well as in pre-pregnancy hypertensive women.

In patients with previous gestational hypertension, chronic renal disease, autoimmune diseases, diabetes, chronic hypertension, complicated by ≥ 1 risk factors of preeclampsia (uniparas, > 40 years old, BMI > 35, family history of preeclampsia, multifetation), low dose aspirin can be prescribed from the twelfth week of the gestation until one week before delivery.[257]

6.4 Hypertension with stroke

Key point 6D

- For patients with stable stroke, antihypertensive therapy should be initiated if BP ≥ 140/90 mmHg, and the target of lowering BP is < 140/90 mmHg (Ⅱa, B).
- For patients with acute ischemic stroke, the BP should be controlled at < 180/110 mmHg if thrombolysis is planned.
- Antihypertensive therapy for acute intracerebral hemorrhage: Aggressively intravenous antihypertensive agents are recommended if SBP > 220 mmHg. Intravenous antihypertensive agents are recommended if SBP > 180 mmHg. The reference goal for lowering BP is 160/90 mmHg (Ⅱb, B).

6.4.1 BP management in stable stroke

Systematic analysis showed that antihypertensive agents significantly reduced the risk of stroke recurrence by 22%.[131] For stable stroke patients, the goal for lowering BP should be < 140/90 mmHg. For patients with stroke or TIA attributable to intracranial atherosclerotic stenosis (stenosis degrees 70%–99%) the recommended BP would be < 140/90 mmHg. For stroke or TIA patients due to hemodynamic reasons, the speed of lowering BP and the range to be maintained should be based on the tolerance and hemodynamics conditions of each patient. Selection of the type and dosage of antihypertensive drugs and BP targets should be individualized after fully considering these three factors: drugs, characteristics of stroke and patient conditions.

6.4.2 Antihypertensive therapy for acute intracerebral hemorrhage

For patients with acute ischemic stroke, the BP should be controlled at < 180/110 mmHg if thrombolysis is planned. Patients with elevated BP within 24 h after ischemic stroke should be carefully treated. Anxiety, pain, nausea, vomiting, and elevated intracranial pressure should be treated in priority. For patients with continuous elevation of BP, SBP ≥ 200 mmHg or DBP ≥ 110 mmHg, or with severe cardiac insufficiency, aortic dissection or hypertensive encephalopathy, antihypertensive agents should be given. Intravenous agents, such as Lorol or Nicardia, could be administered, as well as agents of lowering BP rapidly should be avoided.

Antihypertensive therapy for acute intracerebral hemorrhage: the BP of patients should be evaluated comprehensively and the causes of BP escalation should be investigated. Antihypertensive therapy for acute intracerebral hemorrhage: aggressively intravenous antihypertensive agents are recommended if SBP > 220 mmHg. Intravenous antihypertensive agents are recommended if SBP > 180 mmHg. The reference goal for lowering BP is 160/90 mmHg. Early aggressive antihypertension is safe, but the effectiveness of improving prognosis needs to be further investigated. The BP should be closely monitored during antihypertension therapy. BP should be monitored every 15 min.

6.5 Hypertension with coronary artery disease (CAD)

Key point 6E

- In patients with CAD, it is recommended: the target BP is to < 140/90 mmHg (Ⅰ, A), if tolerated, the target BP is to < 130/80 mmHg (Ⅱa, B).[38, 68] It should be noted that DBP should not be too low (Ⅱb, C).[258–263]
- In patients with stable angina pectoris, beta-blocker or CCB should be recommended first (Ⅰ, A).

6.5.1 Target BP

In patients with CAD, the target BP is to < 140/90 mmHg, if tolerated, the target BP is to < 130/80 mmHg.[38,68] It should be noted that DBP should not be lower than 60 mmHg.[254] In elderly patients or patients with severe coronary stenosis, BP should not be too low.

6.5.2 Selection of antihypertensive drugs for patients with stable angina pectoris

The β-blocker and CCB can reduce myocardial oxygen consumption and angina pectoris attack, so they should be the first choice. If BP control is not ideal, ACEI/ARB and diuretics can be used together.

6.5.3 Selection of antihypertensive drugs for patients with non-ST-segment elevation acute coronary syndrome

The β-blocker and CCB are still the first choice for patients with worsening exertional angina pectoris. If BP control is not ideal, RAS blocker and diuretic can be used together. In addition, when considering the existence of vasospasm factors, large doses of beta-blocker should be avoided as it may induce coronary spasm.

6.5.4 Selection of antihypertensive drugs for patients with acute ST-segment elevation myocardial infarction

Long-term use of β-blocker and RAS blocker can sig-

nificantly improve the prognosis of patients with myocardial infarction as secondary prevention. They should be used early and without contraindications. CCB and diuretic can be used in combination when BP control is not ideal.

6.6 Hypertension with heart failure

Key point 6F

- For patients with both hypertension and heart failure the recommended BP goal is < 130/80 mmHg (Ⅰ, C).
- For patients with heart failure and reduced ejection fraction (HFrEF) the antihypertensives of choice are ACEIs (or an ARB for patients intolerant to ACEIs), β-receptor blockers and aldosterone receptor blockers (Ⅰ, A).

6.6.1 Epidemiology and mechanism

In China, the incidence of hypertension among heart failure patients is 54.6%.[258] The incidence of heart failure in patients with hypertension is 28.9%, stroke (30.0%). Chronic and continuous hypertension eventually leads to heart failure with preserved ejection fraction or heart failure with reduced ejection fraction.[259]

6.6.2 Clinical benefits of antihypertensive therapy

A review of large scale meta-analysis suggests a 10 mmHg reduction in SBP can reduce the incidence of heart failure by 28%.[260] Recent studies among hypertensive patients show that compared to standard BP management (SBP < 140 mmHg), an aggressive BP management strategy (SBP < 120 mmHg) can reduce the incidence of heart failure by 38%, and stroke by 43%.[38]

6.6.3 Antihypertensive goals

The recommended target BP is < 130/80 mmHg, this recommendation is supported by randomized controlled studies. For patients with hypertension and left ventricular hypertrophy, but without heart failure, the BP may be lowered to < 140/90 mmHg,. A target of < 130/80 mmHg has the added advantage of preventing heart failure, in patients with high tolerance levels.

6.6.4 The management of hypertension with chronic heart failure

Hypertension with chronic HFrEF: The drug of choice is ACEI (or ARB in intolerant patients), β-receptor blockers and aldosterone receptor blockers. The concomitant use of these three classes of drugs also forms the basis of the treatment of HFrEF, and can reduce mortality and improve the prognosis, aside from having excellent antihypertensive efficacy. Most of these heart failure patients need to regularly use loop diuretics and thiazide diuretics, which also have good antihypertensive activity. If the above cannot effectively control BP, the use of amlodipine and felodipine are recommended.

Hypertension with HFpEF: the pathogenesis most often involves hypertension, hypertension may be present even after the appearance of symptoms of heart failure. The three drugs mentioned above cannot reduce the mortality and improve the prognosis of these patients. However, these drugs are safe and still recommended for their antihypertensive effect. If the BP still cannot be controlled, the administration of amlodipine or felodipine is recommended. The use of αblockers, centrally acting antihypertensive agents (such as moxonidine) are not recommended. Negatively chronotropic CCBs such as diltiazem and verapamil cannot be used in HFrEF, however can be safely used in HFpEF patients.

6.6.5 Management of hypertensive with acute heart failure

The clinical characteristics include hypertension, primarily left heart failure, rapid progressive, and most often HFpEF. BP needs to be controlled while concomitantly controlling heart failure, primarily by the administration of intravenous diuretics and vasodilators, including nitroglycerin, sodium nitroprussideand urapidil. When mild, BP can be gradually reduced over 24–48 h.When severe with concomitant acute pulmonary edema the reduction in BP should not exceed 25% of baseline in the initial one hour, and may be further reduced to 160/100–110 mmHg in the proceeding 2–6 h, and gradually reduced to normal in 24–48 h.[261,262]

6.7 Hypertension in chronic kidney disease (CKD)

Key point 6G

- We recommend that in CKD and urine albumin excretion < 30 mg/24 h (or equivalence), the goal of treatment with anti hypertension drugs is < 140/90 mmHg (Ⅰ, A), with albuminuria ≥ 30 mg/24 h (or equivalence) the goal is < 130/80 mmHg (Ⅱ A, B).
- We suggest 18–60 years old of hypertension in patients with CKD in ≥ 140/90 mmHg should be evaluated and initiate antihypertensive drug treatment (Ⅰ, A)
- The initial antihypertensive therapy for hypertension in CKD patients should include one ACEI (IIa) or ARB (IIb) alone or in combination with other antihypertensive drugs, but the combination of ACEI and ARB is not recommended (A).[263,264]

6.7.1 The relationship between hypertension and CKD

Hypertension and kidney disease are closely related to each other as etiology and aggravating factors. Hypertension

caused by various CKD, which is called renal hypertension, is mainly divided into renal vascular hypertension and renal parenchyma hypertension. The prevalence of hypertension in non-dialysis CKD patients in China is 67.3%–71.2%.[265,266] while the prevalence of hypertension in dialysis patients is as high as 91.7%.[267]

6.7.2 The goal of lowering BP in CKD patients

Drug antihypertensive therapy was initiated in patients with CKD complicated with hypertension when SBP ≥ 140 mmHg or DBP ≥ 90 mmHg. The target of antihypertensive therapy is < 140/90 mmHg when urine albumin excretion < 30mg/24 h (or equivalence), and < 130/80 mmHg when albuminuria ≥ 30 mg/24 h (or equivalence). The antihypertensive target can be appropriately relaxed for patients over 60 years old.

Proteinuria is a risk factor for renal dysfunction and CVD disease and CVD death in CKD patients. This guideline recommends a more stringent target of 130/80 mmHg BP reduction for patients with proteinuria.

6.7.3 Principles of antihypertensive drugs in CKD patients

ACEI/ARB, CCB, α-receptor blocker, β-blockers, and diuretics can all be used as initial drug choices.

ACEI/ARB not only has antihypertensive effect, but also can reduce proteinuria, delay the decline of renal function, and improve the renal prognosis of CKD patients.[263,264] Initial antihypertensive therapy should include an ACEI or ARB, either alone or in combination with other antihypertensive drugs, the combination of the two drugs is not recommended. ACEI/ARB can be used cautiously even if the serum creatinine increased is less than 30% of basic value, and the dosage reduction or withdrawal can be considered if the serum creatinine increased-exceeds 30% of basic value.

Dihydropyridine and non-dihydropyridine long-acting CCB can be used in the treatment of hypertension in CKD and its renal protection ability mainly depends on its antihypertensive effect.

Thiazide diuretics were effective in patients with GFR > 30 mL/min per 1.73 m^2) (CKD stage 1–3), patients with GFR < 30 mL/min per 1.73 m^2) (stage 4–5 CKD) can use loop diuretics. Diuretics should be given in low dose, excessive diuresis can lead to insufficient blood volume, hypotension, or a decrease in GFR. The combination of aldosterone antagonists with ACEI or ARB may accelerate the risk of renal function deterioration and hyperkalemia.[268]

β-blockers can play a hypotensive role against the excessive activation of the sympathetic nervous system, and alpha beta blockers have a better advantage to play a cardio-renal protective role and can be applied to the hypotensive treatment of patients with CKD at different stages.

Other antihypertensive drugs, such as α-receptor blocker and central alpha receptor agonists, may be used in combination with other antihypertensive drugs as appropriate.

6.7.4 Antihypertensive therapy in dialysis patients with end-stage renal diseases (stage 5 CKD)

Resistant hypertension in CKD5D is an important clinical issue and the blood control needs a combination of more than three antihypertensive medications in most cases. The serum, potassium and creatinine levels should be monitored when receiving RAAS blockers in dialysis patients. Antihypertensive drugs should be avoided at the stage of rapid decrease of dialysis blood volume to avoid severe hypotension. The dosage of antihypertensive drugs should be adjusted to take into account the hemodynamic changes and the clearance of drugs by dialysis. BP measured before dialysis or in the clinic cannot reflect the average BP of dialysis patients very well, so it is recommended to measure the BP of patients at home. BP variation in dialysis patients should not be too large. The ideal target of blood post dialysis is 120–140 mmHg.[269]

6.8 Concomitant hypertension and diabetes mellitus

Key point 6H

- The recommended target BP in patients with diabetes mellitus is less than 130/80 mmHg (II a, B).
- Nonpharmacologic methods lasting less than three months can be instituted in diabetic patients with systolic BP 130–139 mmHg or diastolic BP 80–89 mmHg. If the target BP cannot be achieved by rational nonpharmacologic methods, antihypertensive medication is indicated.
- Patients with confirmed BP ≥ 140/90 mmHg should, in addition to lifestyle therapy, have prompt initiation of pharmacologic therapy to achieve BP targets. In patients also present with established microalbuminuria, pharmacologic therapy should be started directly (I, A).
- Initial choices of medication include ACEI or ARB therapy. When combination therapy is needed, ACEI or ARB is recommended to remain as one of the antihypertensive agents (I, A).

Diabetes mellitus and hypertension often coexist. In Chinese patients with hypertension followed up in outpatient clinic, 24.3% of them also suffered from diabetes.[270] Patients with both diabetes and hypertension are at increased risk for cardio-cerebrovascular morbidity. Studies have showed that antihypertensive therapy significantly reduced the rates of all-cause mortality, cardio-cerebro-

vascular morbidity, and some other adverse clinical outcomes.[271,272]

6.8.1 The BP target level for patients with diabetes

Analysis of the previous studies showed that every 10 mmHg reduction in systolic BP is associated with a 12% reduction in the risk of any diabetes-related complications, and a 15% reduction in the risk of death. Studies also suggested that the lowest incidence of major cardiovascular events occurred at a mean achieved DBP of 82.6 mmHg.[273,274] Currently, the recommended target BP level is 130/80 mmHg for patients with diabetes. For elder diabetic patients and those with severe cardiovascular diseases, BP should be reduced gradually to a slightly higher target of 140/90 mmHg.

6.8.2 The choice and application of antihypertensive agents in patients with diabetes

Nonpharmacologic methods, lasting less than three months, can be instituted in diabetic patients with systolic BP 130–139 mmHg or diastolic BP 80–89 mmHg. If the target BP cannot be achieved by rational nonpharmacologic methods, antihypertensive medication is indicated. Patients with confirmed BP ≥ 140/90 mmHg should, in addition involve themselves in lifestyle therapy and have prompt initiation of pharmacologic therapy to achieve BP targets. In patients, also present with established microalbuminuria, pharmacological treatment should be started directly. Initial choices of antihypertensive agents include ACEI or ARB therapy.[113–115] When combination therapy is needed, diuretics or CCB can be added on ACEI or ARB therapy. For those patients with angina, β-blockers are recommended. Diuretics should be used with caution in diabetic patients with coexisting hyperuricemia. β-blockers should be used with caution in those who are prone to recurrent hypoglycemia, since patients' symptoms of hypoglycemia can be masked by β-blockers. Diuretics and β-blockers should be started in low dosage if prescribed. In patients with BP which cannot be controlled satisfactorily or with prostate hypertrophy, α-blockers might be considered. In general, multiple-drug therapy is often required to achieve BP targets.

6.9 Metabolic syndrome

The prevalence of metabolic syndrome in Chinese adults has increased from 13.8% in 2002 to 18.2% in 2009.[273] The prevalence increases with aging and peaks at 60 to 69 years.[275]

6.9.1 Diagnosis

Metabolic syndrome can be diagnosed with at least three of the following: (1) abdominal obesity, waist circumference 90 cm in male and 85 cm in female or greater; (2) elevated BP: BP 130/85 mmHg or greater, or diagnosed as hypertension and treated; (3) dyslipidemia: fasting triglycerides 1.7 mmol/L (150 mg/dL) or greater, or HDL-cholesterol < 1.04 mmol/L (40 mg/dL), or diagnosed with dyslipidemia and treated; and (4) hyperglycemia: fasting glucose 6.1 mmol/L (100 mg/dL) or greater, or postprandial glucose 2 h after sugar loading 7.8 mmol/L (100 mg/dL) or greater, or diagnosed with diabetes and treated.

Among the components of the metabolic syndrome, hypertension has the highest incidence of 65.4%, followed by dyslipidemia (53.6% in male with hypertriglyceridemia, 49.4% in female with decreased HDL, respectively).[275,276]

6.9.2 Cardiovascular risks

Chinese population studies showed that patients with metabolic syndrome had a 1.85-fold increased risk of 10-year cardiovascular events compared with non-metabolic syndrome patients, and the risk of ischemic and hemorrhagic stroke increased by 2.41 and 1.63 times, respectively. Among the combinations of metabolic syndrome components, abdominal obesity accompanied by hypertension and low HDL-C had the highest cardiovascular risk (increased by 5.25 times), and the risk is further increased by 16.58 times if hyperglycemia is added in.[189,275–277]

6.9.3 Management

Early and comprehensive intervention is crucial in reducing cardiovascular risk and prevents target organ damages for patients with metabolic syndrome.

Treatment lifestyle changes such as healthy diet and regular exercise, are fundamental and effective for metabolic syndrome management. Chinese community studies showed that exercise reduced the risk of metabolic syndrome by 10% to 20%.[278]

ACEI and ARB are recommended as the first-line antihypertensive drugs, especially for patients with diabetes or obesity. Dihydropyridine CCB is also the alternative. Thiazide diuretics and beta-blockers can be used in patients with cardiac dysfunction or CHD.

6.10 Antihypertensive treatment for peripheral arterial disease (PAD)

Key point 6I

- The BP of the patients with lower extremity arterial disease with hypertension should be controlled at < 140/90 mmHg.
- CCB, ACEI or ARB should be adopted first. The application of selective β_1 receptor blockers treating PAD can be considered in condition. Generally, diuretics are not recommended.

PAD is a common manifestation of systemic atherosclerosis. It was shown via epidemiological investigation abroad that the prevalence rate of the same is 3%–10% in the general population, and 15%–20% in the seniors over 70 years old.[279,280] It is 2%–4% in the general population in China, which is as high as 16.4% in the population over 60 years old. It is even higher in patients with risk factors, such as hypertension, diabetes and metabolic syndrome.[281] Approximately half of PAD patients are suffering from hypertension, which also increases the risk of cardiovascular diseases and even death.[282]

BP of the patients with lower extremity arterial disease with hypertension should be controlled at < 140/90 mmHg. The BP targeting not only reduces the incidence of cardiovascular and cerebrovascular events in these patients, but also slows down the progression of the lesion, as well as reduces the amputation rate of the patients.[283,284] The blood flow of the affected limb may reduce during the BP control process, which could be tolerated by most patients.

Drug selection: CCB and RAS inhibitors including ACEI or ARB can improve the endothelial function of the affected blood vessels while reducing BP.[283,285] Generally, selective β_1 receptor blockers can be considered in condition, for they are effective for treating PAD with hypertension, and do not increase the resistance of the affected blood vessels, which has a certain preventive effect on CHD events. Generally, diuretics are not recommended due to their reduction of blood volume and increase of blood viscosity.

6.11 Refractory hypertension

Key point 6J

- Out-of-office BP measurements are often used to determine whether patients are refractory hypertension.
- It is necessary to find out the causes of poor BP control and the coexisting disease factors.
- Conventional dosage of RAS inhibitor + CCB + thiazide diuretics is recommended, and the dosage of each drug should be increased according to patient’s characteristics and tolerance and should reach the full dose.

6.11.1 Definition and cause screening of refractory hypertension

On the basis of improved lifestyle when the application of the three reasonable and tolerable dose of antihypertensive drugs including thiazide diuretics at least four weeks after treatment, the inadequate control of BP is confirmed by the office and out-of-office (including home BP or ambulatory BP monitoring), or at least four drugs are needed to achieve the BP standard, called refractory hypertension. The prevalence of refractory hypertension is unknown, and there is no exact epidemiological data in China.

To determine whether a patient has refractory hypertension, out-of-office BP measurements (home BP measurements and dynamic BP monitoring) are usually required to exclude the effects of White-coat phenomenon and pseudoresistant hypertension.

Look for causes that affect poor BP control and coexisting disease factors: (1) the more common reason is that patients have poor treatment compliance (failure to adhere to medication). (2) Improper selection and use of antihypertensive drugs (unreasonable drug combination and insufficient drug dose). (3) The substances antagonizing hypertension drugs were used, including oral contraceptives, cyclosporine, erythropoietin, glucocorticoids, non-steroidal anti-inflammatory drugs, antidepressants, cocaine and some traditional chinese medicines (such as licorice, ephedra). (4) Other influencing factors are: unhealthy lifestyle, obesity, volume overload (inadequate diuretic treatment, excess salt intake, progressive renal insufficiency); or some coexisting diseases, such as diabetes, dyslipidemia, chronic pain, long-term insomnia, anxiety, *etc*. Patients may have more than one cause that can be corrected or difficult to correct. (5) After eliminating the above factors, we should be alert to the possibility of secondary hypertension and start the screening of secondary hypertension.

6.11.2 Therapeutic principles of refractory hypertension[287–290]

(1) It is recommended that the patient should refer to a hypertensive specialist. The diagnosis of refractory hypertension should be made by a qualified hypertension specialist.

(2) Advocate out-of-office BP measurements (home BP and ambulatory BP) and effective communication with patients. Pay attention to patient compliance with long-term medication.

(3) Nearly possible elimination of influencing factors. They are mainly obesity, metabolic disorders, excessive sodium intake and other unhealthy habits.

(4) Adjust the antihypertensive treatment combined scheme. First, check whether the composition of the multi-drug combination scheme is reasonable. It is recommended to choose the conventional dose of RAS inhibitor+CCB+thiazide diuretics. The dosage of each drug should be increased according to patient's characteristics and tolerance, and the total dose should be reached.

(5) The fourth antihypertensive drug can be added according to the characteristics of patients with unsatisfactory results. The choice can be made between aldosterone receptor antagonists, beta receptor blockers, alpha receptor

blockers or sympathetic nerve inhibitors (clonidine), but the principle of individualized therapy is still required.

6.11.3 Device-based hypertension treatment

Details can be found in 5.5. Advances in Device Intervention.

6.12 Hypertensive emergencies and hypertensive urgencies

Key point 6K

- **Treatment of hypertensive emergencies:** The target of BP reduction in the initial phase (within 1 h) is to reduce MAP by no more than 25% of the untreated level. Reduce BP to a safer level in the next 2 to 6 h, typically around 160/100 mmHg. Reduce BP to normal levels gradually in the next 24 to 48 h if such BP levels can be tolerated.
- **Treatment of hypertensive urgencies:** Slowly reduce BP to 160/100 mmHg in 24 to 48 h. There is no evidence that acute BP lowering management will improve prognosis. The BP reduction of many patients with hypertension urgencies can be achieved with oral medication.

6.12.1 Definition and evaluation

Hypertensive emergencies are situations in which the BP suddenly and severely increases (generally over 180/120 mmHg) in patients with primary or secondary hypertension, with progressive target organ damage such as the heart, brain, and kidney. Including hypertensive encephalopathy, hypertensive intracranial hemorrhage (cerebral hemorrhage and subarachnoid hemorrhage), cerebral infarction, heart failure, acute coronary syndrome (unstable angina, acute myocardial infarction), aortic dissection, pheochromocytoma crisis, using drugs such as amphetamine, cocaine, hallucinogen, *etc*., perioperative hypertension, pre-eclampsia or eclampsia, *etc*.[110,291]

It should be noted that the level of BP is not directly proportional to the extent of acute target organ damage. Some hypertensive emergencies are not accompanied by severe increased BP level, such as acute pulmonary edema, aortic dissection, myocardial infarction, *etc*., while BP is just moderately elevated, but they should also be considered as hypertension emergencies as this kind of situations have a significant impact on target organ function

The term ‘hypertensive urgencies’ is used to describe patients with severe hypertension in whom there is no acute target organ damage. Symptoms such as headache, chest tightness, nosebleeds, irritability, *etc*. caused by severe elevated BP level may be associated with these patients. Most patients’ medication compliance may be poor and have inadequate treatments.

The only criterion to distinguish between hypertensive emergencies and hypertensive urgencies is not the degree of elevated BP level, it is that whether there is occurring a new acute progressive target organ damage. Patients with suspected hypertensive emergencies should be thoroughly evaluated to determine if they are hypertensive emergencies while the initial treatment can not be delayed due to the overall evaluation process.

6.12.2 Treatment of hypertensive emergencies[110, 292]

(1) Therapeutic principles: blood pressure and vital signs should be continuously monitored; the causes and incentives of elevated BP should be eliminated or corrected promptly; the fear can be removed by appropriate usage of effective sedatives; the intravenous antihypertensive drugs should be administrated as soon as possible to control blood pressure in order to prevent further damage to target organs, and the damaged ones can be treated accordingly; and finally, complications should be reduced to improve outcomes.

(2) Choice of treatment drugs: the choice of treatment drugs should be based on the conditions of target organ involved and the patients’ hepatorenal functions. The ideal drug should be able to predict the strength and speed of antihypertension, preserve target organ function, and adjust conveniently. The commonly used drugs for hypertensive emergencies are shown in Table 15. When the blood pressure tends to level off through initial intravenous administration, oral medication can be started and the intravenous drug should be gradually reduced until it can be stopped.

(3) The degree and speed of blood pressure reduction: the blood pressure should be gradually adjusted to the appropriate level without the effect to organ perfusion. The target of BP reduction in the initial phase (within 1 h) is to reduce MAP by no more than 25% of the untreated level. Reduce BP to a safer level in the next 2 to 6 h, typically around 160/100 mmHg. Reduce BP to normal levels gradually in the next 24 to 48 h if such BP levels can be tolerated. For pregnant patients with hypertensive emergencies, blood pressure should be controlled steadily to a relatively safe range (< 150/100 mmHg) as soon as possible, and sudden drop in blood pressure should be avoided lest it affects placental blood circulation.

The degree and speed of blood pressure reduction varies from different target organ damaged in hypertensive emergencies. For patients with acute coronary syndrome and acute left heart failure, it is necessary to reduce blood pressure to the level that can improve cardiac blood supply, reduce myocardial oxygen consumption and improve cardiac function as soon as possible. For patients accompanied with aortic dissection, it is necessary to rapidly reduce blood

Table 15. Intravenous or intramuscular anti-hypertensive drugs for hypertensive emergencies.

Drug name	Dosage	Onset time	Duration	Side effects
Nitroprusside	The intravenous infusion dose stars from 6.25–12.5 μg/min and adjusts it according to blood pressure (perioperative hypertension).<br>0.25–10 μg/kg per minute, IV (hypertension emergencies)<br>Initial dose: 0.3–0.5 g/kg per minute. The dose can be gradually increased according to the blood pressure response; the maximum dose is 10 g/kg per minute (pregnancy-induced hypertension; safety level: C).	Immediately	2–10 min	Hypotension, tachycardia, headache, muscle spasms. Continuous use for more than 48 to 72 h or administration doses > 2 g/ kg per minute may induce cyanide poisoning.
Nitroglycerine	5–100 μg/min, IV (hypertensive emergency with myocardial ischemia).	2–5 min	5–10 min	Headache and vomiting.
Phentolamine	2.5–5 mg, IV (used to diagnose pheochromocytoma and to treat hypertensive episodes caused by it, including hypertension occurred during surgical resection, and can also be used to assist in the diagnosis of pheochromocytoma based on the BP response to it).	1–2 min	10–30 min	Tachycardia, headache and flushing.
Nicardipine	0.5–10 μg/kg per minute, IV, (perioperative hypertension, hypertension emergencies).<br>The initial dose is 5 mg/h, and is gradually increased to 15 mg/h according to blood pressure response (pregnancy-induced hypertension, safety level C).	5–10 min	1–4 h	Tachycardia, headache, peripheral edema, angina pectoris, nausea, dizziness, inhibition of uterine contraction (combined with magnesium sulfate).
Esmolol	0.15–0.3 mg/kg per minute, IVI (perioperative hypertension).<br>250–500 μg/kg, IV<br>Followed by 50–300 μg/kg per minute, IV, (hypertension emergencies).	1–2 min	10–20 min	Hypotension and nausea.
Metoprolol	3–5 mg, IV, repeated every 5 min, the maximum dose is 15 mg (hypertension emergencies).	5–10 min	5–10 h	Hypotension, heart failure, heart block, dizziness, fatigue, depression, bronchospasm.
Labetalol	25–50 mg, IV, repeat every 15min is permitted, and the total dose can be used up to 200 mg. Intravenous infusion is also feasible,<br>1–4 mg/min, IV (perioperative hypertension).<br>20–80 mg IV, 0.5–2.0 mg/min IVI (hypertensive emergency).	5–10 min	3–6 min	Nausea, vomiting, cephalic anesthesia, bronchospasm, conduction block, orthostatic hypotension.
Urapidil	10–50 mg IV<br>6–24 mg/h	5 min	2–8 h	Hypotension, dizziness, nausea, and fatigue.
Enalaprilat	1.25–5 mg IV q6 h	15–30 min	6–12 h	Blood pressure has a steep drop in high renin status, and the variation is high.
Diltiazem	5–10 mg IV, or 5–15 μg/ kg per minute IVI (perioperative hypertension, hypertension emergencies).	5 min	30 min	Tachycardia, AV block, hypotension, cardiac failure, peripheral edema, headache, constipation, hepatotoxicity.
Dralzine	10–20 mg IV<br>10–40 mg IM	10–20 min<br>20–30 min	1–4 h<br>4–6 h	Tachycardia, flushing, headache, vomiting and angina aggravated.
Fenoldopam	0.03–1.6 μg/kg per minute, IV	< 5 min	30 min	Tachycardia, flushing, headache and vomiting.
Magnesium sulphate*	Dilute 5 g magnesium sulphate to 20 mL and inject slowly for 5 min, followed by 1 to 2 g/h IVI for maintenance; or dilute 5 g magnesium sulphate to 20 mL and deep intramuscular injection every 4 h. The total dosage can be used to 25–30 g/d (pregnancy-induced hypertension, severe pre-eclampsia).			The drug should be stopped when urine volume < 600 mL/day, respiration < 16 times/min and tendon reflex disappeared.

*Non-hypertensive drugs. The use of emergency anti-hypertensive drugs is detailed as per the instructions. IV: intravenous injection; IM: intramuscular injection; IVI: intravenous infusion.

pressure to the lowest level required to maintain the basic perfusion of tissues and organs. Generally, it is necessary to use antihypertensive drugs in combination and pay attention to the sufficient dose of β-blockers. If not applicable (such as increased airway resistance), it is feasible to consider switching to non-dihydropyridine CCB.

Notice: Blood pressure control in hypertensive emergencies is to rapidly lowering blood pressure based on ensuring perfusion of important organs. For patients with existed target organ damage, excessively lowering blood pressure can easily lead to the reduction of tissue perfusion pressure and induce ischemic events, which should be avoided.

6.12.3 Treatment of hypertensive urgencies

BP should be slowly reduced to 160/100 mmHg in 24 to 48 hours. There is no evidence that acute BP lowering management will improve prognosis. The BP reduction of many patients with hypertension urgencies can be achieved with oral medication, such as CCBs, ACEIs, ARBs, β-blockers, α-blockers, *etc*., and loop diuretics can also be used if applicable. The initial therapy can be treated in the outpatient service or emergency department, with an observation of 5 to 6 hours after medication. The dose should be adjusted after 2 to 3 days in the clinic, after which long-acting preparation may be used to control blood pressure to the final target level. Patients attended in the emergency department should adjust their regimen of oral medication under the blood pressure is basically controlled, and then adjust their treatment regularly in the outpatient service afterwards. Patients in hypertensive urgencies associated with high risk factors such as cardiovascular disease can also be hospitalized for treatment.

6.13 Perioperative management of hypertension

Key point 6L

- Continuation of beta-blockers or CCB is recommended in hypertensive patients, while ACEI and ARB should be discontinued.
- Target BP for patients under 60 years old should be < 140/90 mmHg. For patients 60 years old or above without diabetes or chronic kidney diseases, SBP should be lowered < 150 mmHg. For patients over 80 years old without diabetes or chronic kidney diseases, SBP should be targeted between 140 and 150 mmHg and < 140/90 mmHg when combined with diabetes or chronic kidney diseases.[110,213,293]

6.13.1 Definition of perioperative hypertension and risk factors[65,294,295]

(1) Definition: perioperative hypertension refers to an elevation of BP (SBP, DBP or PP) over 30% of baseline between the decision of operation and the end of its related therapy, or SBP ≥ 140 mmHg and/or DBP ≥ 90 mmHg during this period. Perioperative hypertensive crisis refers to transient elevation of BP over 180/110 mmHg at this period.

(2) Risk factors: patients with hypertension history, uncontrolled BP before operation, secondary hypertension or intracranial hypertension, psychological factors such as stress, anxiety, fearness or sleeping disorder, especially those with DBP > 110 mmHg, are prone to develop perioperative BP fluctuation.

(3) Operations: perioperative hypertension often occurs in operation of carotid artery, abdominal aorta, peripheral blood vessels, abdominal cavity and thoracic cavity. Severe hypertension may occur in cardiac surgery, large artery operation including carotid endarterectomy and aortic surgery, nervous system operation, head and neck operation, kidney transplantation and severe trauma like empyrosis or head trauma.

6.13.2 Principles of perioperative hypertensive control and BP targets[296]

(1) Principles: basic principle is to ensure perfusion of important organs, reduce cardiac afterload and protect cardiac function. Continuation of β-blockers or CCB is recommended in hypertensive patients, while ACEI and ARB should be discontinued.

(2) BP targets: BP should be controlled < 140/90 mmHg for patients under 60 years old. For patients of 60 years old or above without diabetes or chronic kidney diseases, SBP should be < 150 mmHg. For patients over 80 years old without diabetes or chronic kidney diseases, SBP should be targeted 140 mmHg and 150 mmHg and < 140/90 mmHg when combined with diabetes or chronic kidney diseases.[110,213,293] For selective operation patients with SBP/DBP > 180/110 mmHg when entering operation room, deferring the intervention is recommended. If operation is necessary, such as tumor with hemorrhage, the operation could be performed with family members' approval. Considering possible ischemia of important target organ and side effect of antihypertensive pharmacological therapy, it is not recommended to lower blood pressure in few hours for patients with preoperative SBP/DBP > 180/110 mmHg. In patients with mild to moderate hypertension (SBP/DBP < 180/110 mmHg), there is no evidence of benefit from delaying surgery to optimize therapy.[100,297,298] Emergency operation should be performed for those with life-threatening emergencies without consulting the level of BP. For severe hypertension combined with life-threatening target organ damage or complications, such as those with left heart failure, unstable angina or variant angina pectoris, oliguric renal failure and severe hypokalemia (< 2.9 mmol/L), vital organs function improvement is recommended in the beginning for a brief period

6.13.3 Drug therapy of perioperative hypertension

Intravenous administration of antihypertensive drugs is usually recommended. Immediate target is to lower DBP to 110 mmHg in 30 to 60 min or reduce 10%–15% of DBP (but no more than 25%). Lowering BP to 160/100 mmHg in the following 2 to 6 h is recommended, if tolerated. BP should be lowered faster in patients with aortic dissection and reached the bottom line for maintaining organ perfusion

within 24 to 48 h. Highly reactive drugs are recommended (Table 15).

7 Hypertension prevention, treatment measures and strategies

Key point 7

- Incorporate hypertension prevention into the local medical and health service system and develop corresponding policies, including supervision and evaluation system, resource allocation and personnel arrangement plan.
- Community hypertension prevention and treatment should combine "all populations" and "high-risk groups".
- Hypertension needs lifelong management. Modern information technology ('Internet +' and electronic digital technology) should be applied to aid disease management, and expert services should be facilitated in places where conditions permit.

7.1 Prevention and treatment policy and health service system

It can be prevented and controlled for most of the hypertension pressure, whereas it is not easy to cure. To prevent hypertension effectively, detecting and diagnosing individuals with elevated BP timely, maintaining healthy BP, and managing BP control continuously and systematically, are critical measures to prevent the occurrence of cardiovascular and cerebrovascular diseases, nephrosis, and death in the whole population. Therefore, it requires policy support of the government and involvement of the whole society (those with the disease and those without) to prevent and control hypertension effectively. Prevention and treatment policies should be feasible, cost-effective, and sustainable.

7.1.1 Healthy lifestyles

It requires healthy lifestyles to prevent the pathogenesis of hypertension as well as to control the risk level of it in patients effectively. Departments of the government play a leading role in the improvement of health, which is implemented by the whole society. It's of great value to implement tobacco control measurements, restrict excessive drinking, reduce salt intake, and increase exercise and healthy eating. Except for public education, it needs the support of relevant laws and regulations. Additionally, appropriate sports facilities should be equipped in workplaces, schools and communities. Food choices should be safe and healthy, whereas tobacco and alcohol consumption should be controlled via price and taxation. Relevant departments of cultural and educational media should publicize healthy lifestyles widely, establish role models fulfilling a healthy lifestyle, and guide the public, especially the youth, to have healthy lifestyles. Medical and health service institutions should provide healthy lifestyle guidance for patients as well as medical interventions for those with severe alcohol and tobacco dependence.

7.1.2 Systematic hypertension management

Generally, systematic hypertension management (providing fair and continuous screening, diagnosis, treatment, referral and long-term follow-up for the whole population) is a responsibility shared by the health service system and the whole population, which includes the following four aspects:

(1) Include the prevention and treatment of hypertension into medical and health service policies. (a): Detection and management protocol should be supported based on local hypertension prevalence and economic conditions and preferential policies should be provided for drug treatment in terms of funding. (b): Regular training should be provided for community doctors according to the service areas. (c): Non-clinical doctors, nurses and pharmacists should be allowed to participate in the screening and lifestyle guiding of hypertensive patients after being trained. (d): Smooth two-way referral channels should be provided for patients with complicated or refractory hypertension; and prevention and treatment quality and effect of hypertension should be taken as the main evaluation indicators for medical and health service at all levels regarding their performance indicators.

(2) Once hypertension occurs, it requires lifelong management. Effective management is the key to preventing complications, such as severe cardiovascular and cerebrovascular diseases. The grassroots medical service is the frontier of hypertension prevention and treatment, which ought to be responsible for the detection, recording, treatment and long-term systematic management of hypertension.

(3) Establish and strengthen the unified electronic cardiovascular and cerebrovascular disease management and expert service network in places where conditions permit. A unified electronic information management network in medical and health service system can significantly improve the efficiency of treatment and management of hypertension. In addition, the expert service network regarding cardiovascular and cerebrovascular diseases can provide further education for the grassroots health care providers as well as immediate guidance and advice for patients to improve the management hypertension.

(4) Establish and implement supervision and evaluation system based on medical investigating evidence, with service quality and outcome as the index and cardiovascular and cerebrovascular health as the target of the whole community,

as well as health service policies on resource allocation and personnel arrangements guided by assessment results.

7.2 Community hypertension prevention and treatment strategy

The community should adopt comprehensive prevention and treatment strategies for the whole population, hypertension susceptible (high risk) population and patients in terms of hypertension prevention and treatment, and integrated interventions combining primary, secondary and tertiary prevention.

7.2.1 Strategy for the whole population

Health improvement theory is applied in the strategy for the whole population mainly adopts, with focus on the following aspects: (1) policy development and environment support: healthy lifestyles are called for, with particular focus on reducing salt intake, controlling body weight, promoting early detection and treatment of hypertension, developing policies and creating a supportive environment; (2) Health education: teachers in charge of community health education should strive for the support and cooperation from the local government, and carry out publicity and education on hypertension prevention and control for the whole population in various forms; (3) Community involvement: different departments should cooperate and the whole community should be encouraged to participate in the prevention and treatment of hypertension based on the current health care network; and (4) Facilities intervention: facilities to improve health are divided into five categories: first is the whole city, second is hospitals, third is communities, fourth is workplaces; and fifth is schools. Develop and implement intervention plans on hypertension based on the characteristics of different facilities.

7.2.2 Strategy for population of high-risk (susceptible) to hypertension

It requires early detection of risk factors that may lead to hypertension and effective intervention to prevent the pathogenesis of hypertension in terms of the intervention of high-risk population in the community.

(1) Screening of population susceptible to hypertension: susceptible factors for hypertension include high-normal BP values, overweight and obesity, alcoholism and high salt diet.

(2) Prevention and treatment strategies for population susceptible to hypertension: (a) physical examination: physical examination should include general enquiry, height, weight, BP measurement, routine urinalysis, blood glucose, blood lipids, renal function, electrocardiogram, *etc*; and (b) control of risk factors level: similar to the strategy for general population, perform followed up for management and lifestyle guidance for individuals susceptible to hypertension based on physical examination.

8 Community-based standardized management of hypertension

Key point 8

- Timely detection of hypertension is the first step in prevention and treatment. If there is no condition for the screening of hypertension, the system of "office BP monitoring for first-time visit" should be established. Other opportunities for BP measurement should also be provided.
- Integrate the management of hypertension into the daily medical work of general practitioners. Establish the hierarchical medical system of hypertension with general practitioners as the main body and maintain two-way referral unobstructed. Where conditions permit, a grid information management system should be established step by step.
- Improve patients' knowledge of disease prevention and self-care awareness in various ways. Where conditions permit, the technology of home BP measurement should be properly promoted.

Community-based standardized management of hypertension can improve patients' awareness rate, treatment rate and control rate.[299] As the control rate of hypertension is low at present, it is necessary to standardize and rationalize the use of antihypertensive drugs to improve the status of routine drug treatment of hypertension in China, and then improve the control rate of hypertension.[300]

8.1 Screening and registration of hypertension

BP should be measured in both adult general outpatients for first-time visit and hypertensive patients. Newly diagnosed hypertensive patients need to be registered for the scope of hypertension management.

8.2 Management of first-time visit hypertensive patients

First-time visit	Follow-up
Determine whether there is a target organ damage	BP and related symptoms and signs
Evaluate the possibility of secondary hypertension	Side effects of treatment
Comprehensive cardiovascular risk assessment and determine whether to intervene in other cardiovascular risk factors.	Obstacles affecting lifestyle changes and drug treatment compliance
Provide lifestyle guidance and medication.	
Determine the next follow-up date.	
Recommend home BP monitoring.	
Registered for the scope of hypertension management.	

8.3 Hierarchical long-term follow-up for hypertension management

According to the conditions of primary health service institutions and doctors, it is suggested that, in the long-term follow-up for hypertension patients, the management should be divided into I and II levels. Management levels are determined according to whether the patients reach target BP. The main content of follow-up is to observe BP, medication, adverse reactions. Other risk factors should also be paid attention to, such as heart rate, blood lipid, blood sugar, target organ damage and clinical diseases. Hierarchical management can effectively utilize existing resources so as to focus on the management of patients with uncontrolled hypertension, and improve the BP control rate. The contents of hierarchical follow-up management are shown in Table 16.

According to the risk factors, target organ damage and concomitant clinical diseases, blood sugar, blood lipid, renal function, urine routine and electrocardiogram can be examined regularly or irregularly.

The main methods of follow-up for hypertension are outpatient follow-up and telephone follow-up. Follow-up can be also carried out online especially for young and middle-aged people.

Table 16. The contents of hierarchical follow-up for hypertension management.

Items	Management level I	Management level II
Management object	Patients with controlled hypertension	Patients with uncontrolled hypertension
Non-drug therapy	Long-term adherence	Strengthen lifestyle interventions for a long time
Follow-up frequency	Once every three months	Once every 2–4 weeks
Drug therapy	Maintenance drug therapy<br>Keep BP up to standard	Adjust treatment regimen according to guidelines

Follow-up content: BP level, treatment measures, adverse reactions, intervention of other risk factors, treatment of clinical situation, *etc*.

8.4 Health education for hypertensive patients[301]

The hypertension management team is responsible for the health education of hypertensive patients. The main contents include:

Normal population	High-risk population of hypertension	Diagnosed hypertensive patients
· What is hypertension, the danger of hypertension, healthy lifestyle, regular BP monitoring<br>· Hypertension is preventable.	· What is hypertension, the danger of hypertension, healthy lifestyle, regular BP monitoring<br>· Risk factors for hypertension. Targeted behavioral modification and lifestyle guidance.	· What is hypertension, the danger of hypertension, healthy lifestyle, regular BP monitoring<br>· Risk factors for hypertension. Targeted behavioral modification and lifestyle guidance.<br>· Risk factors for hypertension and the comprehensive management.<br>· Importance of non-drug therapy and long-term follow-up and necessity of lifelong treatment.<br>· Hypertension is treatable. Realize the efficacy and side effects of hypertensive drugs.<br>· Capability of hypertension self-management.

8.5 Remote management of hypertensive patients

According to local conditions, each region can actively establish a clinical information system and a chronic disease management information system including hypertension.

Where conditions permit, a remote management platform for hypertension and related diseases can be further established. Patients can measure BP outside hospital by using electronic sphygmomanometer with remote transmission function. Doctors can monitor patients' BP data remotely. So that patients can get doctor's guidance and suggestions without leaving home. The dynamic management of patients' BP outside hospital between outpatient service and follow-up can also be realized. So as to improve patients' treatment compliance and enhance the quality of hypertension management in primary health service institutions.

8.6 Team building

Community health service centers should set up hypertension management teams consisting of doctors, nurses and health accountants (or physician assistant). The team members should receive regular training, and share the responsibility of hypertension management. Team members should have a clear division of labor and responsibilities, and develop team workflow.

8.7 Hierarchical medical system of hypertensive patients

With the advancement of hierarchical medical system reform, the functional orientation of hypertension diagnosis and treatment in medical institutions at all levels should be gradually clarified. General practitioners are the main force of hypertension prevention and treatment. The management

of hypertension should be integrated into the daily medical work of general practitioners. The two-way referral channels should be opened to further improve the control rate of hypertension.

8.7.1 Transfer conditions for hypertension in community first-visit

(1) Complicated with severe clinical condition or target organ damage, further evaluation and treatment are needed;

(2) The result of multiple BP measurements is up to grade 3 and further evaluation and treatment are needed;

(3) Suspected patients with secondary hypertension;

(4) Pregnant and lactating women;

(5) Hypertensive emergencies and sub-emergencies;

(6) Further examination is needed in upper hospitals for diagnosis.

8.7.2 Transfer conditions for hypertension in community follow-up

(1) BP of patients which has still not reached the target after being treated with more than two antihypertensive drugs; (2) patients with previously stable BP control, the BP rises again and is difficult to control; (3) the BP fluctuates greatly and the clinical treatment is difficult; (4) new serious clinical diseases or worsening of original diseases occurred during follow-up; (5) unexpected or intractable adverse reactions occurred after taking antihypertensive drugs; and (6) patients with multiple hypertension risk factors or target organ damage and difficult to deal with.

8.7.3 Transfer conditions for patients from superior hospitals to communities

(1) The diagnosis of hypertension is clear; (2) the treatment regimen has been determined; and (3) BP and its accompanying clinical conditions have been controlled and stabilized.

8.8 Self-management of hypertensive patients

All hypertensive patients should participate in self-management to varying degrees. (1) Improving compliance: General practitioners should use their knowledge, skills and resources to help patients in their preferred ways. In order to enhance their initiative in preventing and treating hypertension and compliance with antihypertensive drugs. (2) Patient self-management group: By combining with the neighborhood committee or village committee, the patient self-management group should carry out education for hypertensive patients. (3) Family BP measurement: Guide patients to carry out family self-measurement of BP. It is recommended that patients use the upper arm automatic sphygmomanometer qualified by international standards to measure BP when conditions permit. Guide patients to master measurement technology and standardize operation, record BP measurement results truthfully. Provide the BP data to medical staff for treatment reference during follow-up.

9 Secondary hypertension

Key point 9

- Conventional Secondary hypertension screening should be performed in patients with newly diagnosed hypertension.
- Patients with Resistant hypertension should be considered as having the possibility of secondary hypertension and should be advised to the relevant specialist when necessary.

Secondary hypertension, also known as symptomatic hypertension, is one of the symptoms of some diseases in their developments. When the primary disease is cured, BP will also drop or go back to normal. In addition to the harm caused by hypertension itself, the electrolyte disturbance, endocrine imbalance, hypoxemia that associated with secondary hypertension can also lead to the cardiovascular damage independent of BP, which is more harmful than primary hypertension. Therefore, early identification and treatment are particularly important. Conventional secondary hypertension screening should be performed in patients with newly diagnosed hypertension. Patients with resistant hypertension should be considered as the possibility of secondary hypertension and should be advised to the hypertension specialty or endocrine and nephropathy specialty when necessary.

9.1 Renal parenchymal hypertension

Common diseases that leads to renal parenchymal hypertension include various primary glomerulonephritis (IgA nephropathy, focal segmental glomerular sclerosis, membranoproliferative glomerulonephritis, *etc*.); polycystic kidney disease; tubulointerstitial disease (chronic pyelonephritis, obstructive nephropathy, reflux nephropathy, *etc*.); metabolic disease and renal damage (diabetic nephropathy, *etc*.); systemic or connective tissue disease. Renal damage by systemic or connective tissue diseases (lupus nephritis, scleroderma, *etc*.); monoclonal immunoglobulin-related kidney disease (light chain deposition disease); hereditary kidney disease (Liddle syndrome, *etc*.).

The diagnosis of renal parenchymal hypertension depends on: history of kidney disease; proteinuria and hematuria; abnormal renal function; decreased eGFR; abnormal size and shape of kidney; and should take pathological biopsy of kidney when necessary. At the same time, it should

be differentiated from the kidney damage caused by hypertension, the former often occurs before or at the same time with hypertension with high and uncontrollable BP; proteinuria/hematuria occurs early and severely, and the renal function is significantly damaged.

Patients with renal parenchymal hypertension should be given a low-salt diet (NaCl < 6.0 g/day, Na < 2.3 g/day). For patients with renal insufficiency, it is advisable to choose high-quality protein with high biological value (0.3–0.6 g/kg per day) to ensure adequate energy intake and plus alpha-ketoacid therapy. The target BP is 130/80 mmHg. The first choice for patients with proteinuria should be ACEI or ARB. Long-acting CCB, diuretics, beta-receptor blockers and alpha-receptor blockers can be used as combined therapy drugs.

9.2 Hypertension caused by renal artery stenosis and other vascular diseases

9.2.1 Renal artery stenosis

The main characteristic of renal artery stenosis is the stenosis at the main trunk or branch of renal artery, which leads to renal ischemia, significant increase of renin-angiotensin system activity thereby causing hypertension and renal hypofunction. Renal artery stenosis is one of the most important causes of hypertension and/or renal insufficiency. The prevalence of renal artery stenosis is about 1%–3% of in hypertensive patients. In China, atherosclerosis is the most common cause of renal artery stenosis, accounting for about 82%, followed by arteritis (about 12%), fibromuscular dysplasia (about 5%) and other causes accounting for 1%.

The purposes of diagnosing renal artery stenosis include: (1) defining the causes; (2) defining lesion site and degree; (3) hemodynamic significance; and (4) whether can benefit from vascular reconstruction. Transarterial angiography is still the gold standard for the diagnosis of renal artery stenosis. Antihypertensive drugs treatment is the basic treatment for renal vascular hypertension. CCB is safe and effective. ACEI or ARB is the most targeted drug, but it should be cautiously used in patients with unifunctional or bilateral renal artery stenosis. For severe renal artery stenosis with pathophysiological significance (diameter stenosis > 70%), for example: if BP is poorly controlled, kidney atrophy or renal dysfunction occurred, revascularization is recommended.[309] Endovascular therapy is the first choice for vascular reconstruction. Open surgery is recommended for failed lesions.

9.2.2 Stenosis of aorta

Aortic stenosis includes congenital and acquired aortic stenosis. Congenital aortic stenosis manifests as limited stenosis or atresia at the aorta, the onset site is usually near the orifice of the original ductus arteriosus in the isthmus of the aorta, while some of which may occur in other parts of the aorta. Acquired aortic stenosis includes Takayasu arteritis, atherosclerosis, and aortostenosis causes by Aortic dissection. The basic pathophysiological changes of the disease are blood flow redistribution caused by stenosis, water and sodium retention and RAS activation caused by renal ischemia, which results in left ventricular hypertrophy, heart failure, cerebral hemorrhage and other important organ damages. Aortic stenosis manifests as hypertension in upper limbs, weak or no arteries in lower limbs. The BP of both lower limbs was significantly lower than that of upper limbs (ABI < 0.9). There was obvious vascular murmur around stenosis vessels during auscultation. There was obvious vascular murmur around stenosis vessels during auscultation. Endovascular therapy or open surgery was selected according to the specific condition. Active Takayasu arteritis should be treated with glucocorticoids and immunosuppressive agents.

9.3 Obstructive sleep apnea syndrome

Obstructive Sleep Apnea Syndrome (OSAS) includes muscle collapse in upper respiratory tract during sleep, apnea or significant reduction of oronasal airflow, resulting in intermittent hypoxia, sleep fragmentation, sympathetic hyperexcitability, neurohumoral dysregulation and so on. The incidence of hypertension in these patients ranges from 35% to 80%.[310]

Polysomnographic breathing monitor (PSG) is the "golden standard" for diagnosing OSAS. Apnea hypopnea index (AHI) refers to the average number of sleep apnea hypopnea in per hour. According to AHI, it can be divided into mild, moderate and severe degrees, mild: AHI 5–15 times/h; moderate: AHI 15–30 times/hour; severe: AHI more than 30 times/h.

Improving life style is the basis of treatment, which includes losing weight, proper exercise, smoking cessation, alcohol restriction, lateral sleeping, *etc*. For patients with mild OSAS, oral appliance is recommended; for patients with mild OSAS with obvious symptoms (such as daytime sleepiness, cognitive impairment, depression), cardio-cerebrovascular diseases and diabetes mellitus, and for patients with moderate to severe OSAS (AHI > 15 times/h), non-invasive ventilation (CPAP) is recommended.

9.4 Primary aldosteronism and other endocrine hypertension

9.4.1 Primary aldosteronism

Primary aldosteronism is a clinical syndrome characterized by hyperaldosterone secretion in the globular zone of

the adrenal cortex, which leads to hypertension, hypokalemia and renin activity inhibition. Common types of primary aldosteronism include aldosteronism (35%) and idiopathic aldosteronism (60%). Other rare types are adrenal cortical carcinoma and familial aldosteronism, such as glucocorticoid-inhibiting aldosteronism (GRA). Primary aldosteronism accounts for about 5%–10% of hypertensive patients and only a small percentage of them have hypokalemia, which accounts for about 20% of refractory hypertension. It can increase the risk of metabolic syndrome, arteriosclerosis and cardiovascular and cerebrovascular diseases.[311,312]

The clinical diagnosis process of primary aldosteronism includes three steps: screening, diagnosis and classification. Serum aldosterone/renin ratio (ARR) was used for screening.[301] The screening subjects were refractory hypertension, hypertension with spontaneous or diuretic-induced hypokalemia, or adrenal accidental tumors, primary aldosteronism in first-degree relatives, sleep apnea syndrome, early onset hypertension or family history of cardiovascular events (< 40 years old). The confirmation tests include high sodium diet test, intravenous saline test, fluorocortisone inhibition test and captopril test. The classification diagnostic method includes adrenal imaging examination and lateral adrenal venous sampling (AVS). Patients with surgical indication and intention should undergo AVS. While, for those younger than 35 years of age and have typical manifestations (hyperaldosterone, PRA depression, hypokalemia, unilateral adrenal space–occupying lesions), AVS can be avoided. Treatment includes surgery and drug therapy. Patients younger than 35 years of age with unilateral adenomas or large nodules (> 1 cm) or unilateral dominant secretory adenomas or nodules confirmed by AVS were should take surgical treatment.

Drug therapy should be taken for those who have no surgical indication, intention or intolerance. The first-line drug is salt corticosteroid receptor antagonist, and spironolactone is recommended as the first choice.

9.4.2 Pheochromocytoma/paraganglioma

Pheochromocytoma is a neoplasm originating from adrenal medulla or pheochromocyte of extra adrenal nerve chain. Tumor can secrete excessive catecholamine (CA), which can cause persistent or paroxysmal hypertension, functional and metabolic disorders on some organs. It is a clinically curable secondary hypertension. The clinical features of pheochromocytoma appeared to be paroxysmal, persistent or paroxysmal severe hypertension. Hypertension is often accompanied by headache, palpitation and hyperhidrosis, also abnormal glucose and lipid metabolism.

The main method of qualitative diagnosis of pheochromocytoma is determination of catecholamines and its metabolites. Enhanced CT is recommended as the preferred locational method for thoracic, abdominal and pelvic lesions. MRI is recommended as the preferred locational method for skull base and neck lesions. Besides, metaiodobenzylguanidine (MIBG), 18F-FDG PET and somatostatin imaging can be used to make function and image localization for metastatic and extraadrenal tumors. Surgical treatment is important for patients with pheochromocytoma. Alpha-receptor blockers can be administered before surgery. Using beta-blockers before using alpha-blockers is not recommended. Lifelong follow-up should be taken after surgery.

9.4.3 Cushing's syndrome

Cushing's syndrome (CS) that is hypercortisolism. Hypercortisolism can be accompanied by various complications, leading to the syndrome with typical manifestations like centripetal obesity, hypertension, abnormal glucose metabolism, hypokalemia and osteoporosis.

As Qualitative and locational diagnosis of CS and its treatment is more complicated, it is suggested to communicate and cooperate with doctors in hypertension specialty or endocrinology department actively. ACEI or ARB antihypertensive drugs are preferred to use in the initial treatment of Cushing's syndrome-related hypertension.

If BP is still higher than 130/80 mmHg, they can be combined with mineralocorticoids receptor antagonists or CCB according to the severity of the disease and the presence or absence of hypokalemia. If BP is still higher than 130/80 mmHg, the alpha-receptor blocker or nitric acid can be added. If BP still cannot reach the standard, beta-blocker and diuretic can be carefully selected and used.

9.5 Other rare types of secondary hypertension

According to the present epidemiological data, there are still some rare causes of elevated BP, which account for less than 1% of the causes of hypertension, mainly includes thyroid dysfunction, hyperparathyroidism, renin tumors and so on.

9.6 Drug-induced hypertension

Drug-induced hypertension is caused by the drug itself in a conventional dose or its interactions with other drugs, and when BP exceeds 140/90 mmHg, it should be considered as drug-induced hypertension (Table 17). The drugs involved mainly include: (1) hormone drugs; (2) central nervous drugs; (3) non-steroidal anti-inflammatory drugs; (4) Chinese herbal medicine; and (5) other drugs.

Table 17. Drugs, mechanisms and treatment of drug-induced hypertension.

Category	Common drugs	Mechanism	Treatment and notes
**Hormones**			
Estrogen	Estradiol, ilestriol<br>premarin, desogestreland ethinylestradiol	(1) Water-sodium retention<br>(2) RAS activation<br>(3) Insulin resistance	Diuretic<br>ACEI (ARB)<br>β-blocker
Progestogen	Medroxyprogesterone 17-acetate, norethindrone, medroxyprogesterone acetate	Corticosteroid responses induced by	High doses of drugs
Androgen	MethyltestosteroneNandroloniPhenyl-Propionas, stanozolol	(1) Induce polycythemia<br>(2) Affecting the regulation of potassium channels and androgen receptors, leading to retention of nitrogen, sodium, potassium and phosphorus and insulin resistance	
Oxytocin		Antidiuretic effects will occur in large doses	
Pituitrin		(1) Arterioles contraction<br>(2) Increased water reabsorption	
Glucocorticoid	Hydrocortisone,<br>Prednisone, Dexamethasone	Both cortisol and corticosterone have salt corticosteroid activity	Pay attention to the change of serum potassium<br>Diuretic CCB<br>ACEI (ARB)
Mineralocorticoid	9α-Fludrocortisone,<br>Deoxycortone Acetate	Increase sodium reabsorption and Promote potassium excretion	Diuretic<br>Pay attention to the change of serum potassium
Thyroxine Sodium	Euthyrox	Increase the excitability of sympathetic nervous system	
Affecting sympathetic excitation			
Anesthetics	Ketamine, desflurane, sevoflurane, naloxone hydrochloride,	Increased sympathetic excitability	α receptor blockers, clonidine, diltiazem,
	ritali, amphetamine, cocaine	Promote the release of dopamine and NE from nerve endings and block their recovery, increase synaptic sites and prolong the action time	α receptor blockers, verapamil, nitroglycerin
Antiparkinsonian drug	L-DOPA	Stimulate dopamine receptors at the postsynaptic membrane to play the role of antiparkinsonian and elevate BP at the same time	
Diet pills	Sibutramine	Inhibit the re-uptake of 5-hydroxytryptamine and NE, increase the content of synaptic space and the excitability of sympathetic nerve	Lose weight in other ways<br>ACEI (ARB)<br>β-blocker
Adrenaline β_2-agonist	Salbutamol sulfate, bambuterol hydrochloride, terbutaline sulfate, clorprenaline	Activate the adenylate cyclase and increase the synthesis of adenosine cyclophosphate in cells	Should be used with caution for pheochromocytoma or hyperthyroidism
Methylxanthines	Aminophylline, doxofylline, dyphylline	Increased the release of endogenous epinephrine and norepinephrine	
**Nonsteroidal anti-inflammatory drugs**			
	Indomethacin, indomethacin, ibuprofen, phenylbutazone celebrex, arthrotec, antine	(1) Water-sodium retention, (2) reduce the prostaglandins in circulation, (3) renal damage	CCB<br>ACEI (ARB)
**Chinese herbal medicine**			
Glycyrrhizin	Glycyrrhizin, cholic acid, carbenoxolone	(1) Inhibits the activity of 11beta-hydroxysteroid dehydrogenas, increase the production of cortisol-mediated mineral corticoid and elevate BP<br>(2) Inhibit the synthesis of prostaglandin<br>(3) Inhibit the synthesis and release of histamine	Diuretics<br>CCB<br>ACEI (ARB)
Ephedrines	Ephedrine nasal drops, ephedrine and chlorphenamine, diphenhydramine	(1) Directly activate adrenaline α and β2 receptors<br>(2) Indirectly promote the release of NE neurotransmitters<br>(3) Significant central stimulating	α receptor blocker<br>β receptor blocker

Table 17. Cont.

Category	Common drugs	Mechanism	Treatment and notes
**Others**			
Monoamine oxidase inhibitor	Isoniazid, furazolidone, ketoconazole; Reserpine; TCA	Antagonize monoamine oxidase and other enzymes, it is not beneficial to the inactivation of catecholamines both inside and outside the cell, therefore enhances vasoconstriction.	α receptor blocker
TZDs	Rosiglitazone maleate, pioglitazone	Water-sodium retention	Use cautiously in patients with severe heart failure
Recombinant human erythropoietin		(1) Vasoconstriction and intracellular calcium homeostasis and increase sympathetic excitability<br>(2) Stimulate the synthesis of endothelin in vascular endothelial cells<br>(3) Polycythemia<br>(4) Genetic mechanisms	Preferred CCB or α receptor blocker, Diuretics and ACEI are not sensitive for antihyper-tensive effects
Ciclosporin and immuno-suppressant	Cyclosporin<br>Tacrolimus	(1) Activation of sympathetic nervous system<br>(2) Diuretic response is unresponsive when dilatation of blood volume happen<br>(3) NO-mediated vasodilation is impaired and endothelin release increased<br>(4) Afferent nerve of renal sympathetic nerve is activated after neurocalcin was blocked	CCB (may increase in serum cyclosporin concentration)<br>Combination of multiple antihypertensive drugs<br>(Clonidine)

ACEI: angiotensin converting enzyme inhibitor; ARB: angiotensin receptor blockers; CCB: calcium channel blockers.

9.7 Monogenic inherited hypertension

The mutations of monogenic inherited hypertension are mostly associated with gene mutations on renal unit ion transporters or RAS components causing dysfunction. It can be divided into the following categories: (1) Gene mutations directly affect the function of related proteins in renal tubu--lar ion channel transport system: Liddle syndrome, Gordon syndrome, apparent mineralocortixoid excess, pregnancy-I resistancenduced hypertension caused by corticosteroid receptor mutations (2) abnormal steroid synthesis in the adrenal gland caused by Gene mutations: familial hyperaldosteronism (Ⅰ, Ⅱ, Ⅲ), congenital adrenal hyperplasia (11β-hydroxylase deficiency, 7 α-hydroxylase/17, 20-lyase deficiency, 17OHD), familial glucocorticoid resistance (3) Various neuroendocrine tumors, such as pheochromocytoma, hypertension with brachydactylia, multipleendocrineneoplasm (MEN) and Von Hippel-Lindau (VHL).

10 Research prospection

In 2015, researchers from Chinese Center for Disease Control and Prevention published an article focusing on the burden of disease in China, showing that from 1990 to 2013 the age-standardized death rate of stroke decreased by 21% and that rate of hemorrhagic stroke decreased by 38% respectively.[313] Stroke, especially hemorrhagic stroke, is a major complication of hypertension, therefore the results reflects the hypertension prevention and treatment has achieved some effect in terms of cardiac outcomes. Presently, the important ongoing hypertension researches in China include Study of Antihypertensive Treatment in Patients with High-normal Blood Pressure and Risk Factors (CHINOM), Optimal Blood Pressure and Cholesterol Targets for Preventing Recurrent Stroke in Hypertensives (SHOT), Strategy of Blood Pressure Intervention in the Elderly Hypertensive Patients (STEP), Systolic hypertension in the elderly: Chinese Trial-2 (Syst-China-2), Intervention Study of Blood Pressure in People with High Normal or Borderline Hypertension and Type 2 Diabetes (IPAD), China Stroke Primary Prevention Trial-2 (CSPPT-2) and so on. The report of *Comparative Effect Study of L-amlodipine Maleate and Amlodipine Besylate in the Hypertensive Patients (LEADER Study)*, supported by the National Science and Technology Major Project for Drug Discovery, is being summarized, and important results will be released for clinical reference. The CHIEF study will also continue to summarize data on cardiovascular events.

Announcement of conflict of interest (This list is in no particular order.)

During the revision of this guideline, AstraZeneca Investment (China) Co., Ltd., Bayer Health Care Co., Ltd., Beijing Double-Crane Pharmaceutical Co., Ltd., Sinopharm Holding Distribution Co., Ltd., Hanhui Pharmaceutical Co., Ltd., Hangzhou MSD Pharmaceutical Co., Ltd., Lepu Pharmaceutical Technology Co., Ltd., Merck Serono Co., Ltd., Sanofi (Hangzhou) Pharmaceutical Co., Ltd., Shenzhen Salubris Pharmaceutical Co., Ltd., Servier (Tian-

jin) Pharmaceutical Co., Ltd., Tianjin Tanabe Pharmaceutical Co., Ltd., Tibet Kangzhe Pharmaceutical Development Co., Ltd., Nanjing Chia Tai Tianqing Pharmaceutical Co., Ltd., Jiangsu Simcere Pharmaceutical Co., Ltd., Beijing Nuohe Xinkang Gene Technology Co., Ltd., and Beijing Precision Health Management of Hypertension & Angiocardiopathy Co., Ltd. supported the conferences of revision, but did not participate in or influence the guideline's academic content and evidence review. The guideline remains independent.

References

1 Wang Z, Chen Z, Zhang L, *et al*. Status of Hypertension in China: results from the China hypertension survey, 2012–2015. *Circulation* 2018; 137: 2344–2356.

2 Li LM, Rao KQ, Kong LZ, *et al.* [Survey on Nutrition and Health Status of Chinese Residents in 2002]. *Chin J Epidemiol* 2005; 26: 478–484. [In Chinese].

3 Hu YS, Yao CH, Wang WZ, *et al*. [Prevalence of hypertension in some ethnic groups in China in 2002]. *Wei Sheng Yan Jiu* 2006; 35: 573–575. [In Chinese].

4 Gu D, Wildman RP, Wu X, *et al*. Incidence and predictors of hypertension over 8 years among Chinese men and women. *J Hypertens* 2007; 25: 517–523.

5 Lewington S, Lacey B, Clarke R, *et al*. The burden of hypertension and associated risk for cardiovascular mortality in China. *JAMA Intern Med* 2016; 176: 524–532.

6 Wu Y, Huxley R, Li L, *et al*. Prevalence, awareness, treatment, and control of hypertension in China: data from the China National Nutrition and Health Survey 2002. *Circulation* 2008; 118: 2679–2686.

7 Fan GH, Wang ZW, Zhang LF, *et al.* [Survey of prevalence, awareness, treatment rate and control rate of hypertension in rural areas in four northern districts in 2013]. *Chine J Med* 2015; 95: 616–620. [In Chinese].

8 Yang L, Yan J, Tang X, *et al*. Prevalence, awareness, teatment, control and risk factors associated with hypertension among adults in Southern China, 2013. *PLoS One* 2016; 11: e0146181.

9 Gu H, Li W, Yang J, *et al*. Hypertension prevalence, awareness, treatment and control among Han and four ethnic minorities (Uygur, Hui, Mongolian and Dai) in China. *J Hum Hypertens* 2015; 29: 555–560.

10 Wang G, Li LM, Hu YH, *et al.* [Study on the relationship between risk factors and risk of hypertension in community population in Shanghai]. *Chin J Epidemiol* 2013; 34: 307–310. [In Chinese].

11 Gu D, Gupta A, Muntner P, *et al*. Prevalence of cardiovascular disease risk factor clustering among the adult population of China: results from the International Collaborative Study of Cardiovascular Disease in Asia (InterAsia). *Circulation* 2005; 112: 658–665.

12 Li Y, Feng X, Zhang M, *et al*. Clustering of cardiovascular behavioral risk factors and blood pressure among people diagnosed with hypertension: a nationally representative survey in China. *Sci Rep* 2016; 6: 27627.

13 Ying CQ, Fu SB, Xu Q, *et al*. Multiple risk factor clustering and risk of hypertension in the Mongolian ethnic population of China. *Biomed Environ Sci* 2007; 20: 381–385.

14 Elliott P, Stamler J, Nichols R, *et al*. Intersalt revisited: further analyses of 24 hour sodium excretion and blood pressure within and across populations. Intersalt Cooperative Research Group. *BMJ* 1996; 312: 1249–1253.

15 National Health and Family Planning Commission Disease Prevention and Control Bureau. [Report on Nutrition and Chronic Disease Status of Chinese Residents (2015)]. Beijing: People's Medical Publishing House 2015; 33–50. [In Chinese].

16 Liu Z. Dietary sodium and the incidence of hypertension in the Chinese population: a review of nationwide surveys. *Am J Hypertens* 2009; 22: 929–933.

17 Global BMIMC, Di Angelantonio E, Bhupathiraju Sh N, *et al*. Body-mass index and all-cause mortality: individual-participant-data meta-analysis of 239 prospective studies in four continents. *Lancet* 2016; 388: 776–786.

18 Wang ZW, Hao G, Wang X, *et al.* [Aggregation analysis of overweight/obesity and cardiovascular risk factors in middle-aged population in China]. *Chin J Hypertens* 2014; 35: 1000. [In Chinese].

19 Feng BY, Chen JC, Li Y, *et al.* [A follow-up study on the relationship between overweight and obesity and hypertension in Chinese adults]. *Chin J Epidemiol* 2016; 37: 606–611. [In Chinese].

20 Wang Z, Zeng X, Chen Z, *et al*. Association of visceral and total body fat with hypertension and prehypertension in a middle-aged Chinese population. *J Hypertens* 2015; 33: 1555–1562.

21 Fox CS, Massaro JM, Hoffmann U, *et al*. Abdominal visceral and subcutaneous adipose tissue compartments: association with metabolic risk factors in the Framingham Heart Study. *Circulation* 2007; 116: 39–48.

22 Xin X, He J, Frontini MG, *et al*. Effects of alcohol reduction on blood pressure: a meta-analysis of randomized controlled trials. *Hypertension* 2001; 38: 1112–1127.

23 Holmes MV, Dale CE, Zuccolo L, *et al*. Association between alcohol and cardiovascular disease: Mendelian randomisation analysis based on individual participant data. *BMJ* 2014; 349: g4164.

24 Lambert E, Dawood T, Straznicky N, *et al*. Association between the sympathetic firing pattern and anxiety level in patients with the metabolic syndrome and elevated blood pressure. *J Hypertens* 2010; 28: 543–550.

25 Bajko Z, Szekeres CC, Kovacs KR, *et al*. Anxiety, depression and autonomic nervous system dysfunction in hypertension. *J Neurol Sci* 2012; 317: 112–116.

26 Pan Y, Cai W, Cheng Q, *et al*. Association between anxiety

and hypertension: a systematic review and meta-analysis of epidemiological studies. *Neuropsychiatr Dis Treat* 2015; 11: 1121–1130.
27 Dong GH, Qian ZM, Xaverius PK, *et al*. Association between long-term air pollution and increased blood pressure and hypertension in China. *Hypertension* 2013; 61: 578–584.
28 Shang Y, Sun Z, Cao J, *et al*. Systematic review of Chinese studies of short-term exposure to air pollution and daily mortality. *Environ Int* 2013; 54: 100–111.
29 Lewington S, Clarke R, Qizilbash N, *et al*. Age-specific relevance of usual blood pressure to vascular mortality: a meta-analysis of individual data for one million adults in 61 prospective studies. *Lancet* 2002; 360: 1903–1913.
30 Lawes CM, Rodgers A, Bennett DA, *et al*. Blood pressure and cardiovascular disease in the Asia Pacific region. *J Hypertens* 2003; 21: 707–716.
31 Lloyd-Jones DM, Larson MG, Leip EP, *et al*. Lifetime risk for developing congestive heart failure: the Framingham Heart Study. *Circulation* 2002; 106: 3068–3072.
32 Conen D, Tedrow UB, Koplan BA, *et al*. Influence of systolic and diastolic blood pressure on the risk of incident atrial fibrillation in women. *Circulation* 2009; 119: 2146–2152.
33 Klag MJ, Whelton PK, Randall BL, *et al*. Blood pressure and end-stage renal disease in men. *N Engl J Med* 1996; 334: 13–18.
34 Dolan E, Stanton A, Thijs L, *et al*. Superiority of ambulatory over clinic blood pressure measurement in predicting mortality: the Dublin outcome study. *Hypertension* 2005; 46: 156–161.
35 Johansson JK, Niiranen TJ, Puukka PJ, *et al*. Prognostic value of the variability in home-measured blood pressure and heart rate: the Finn-Home Study. *Hypertension* 2012; 59: 212–218.
36 Chen WW, Gao RL, Liu LS, *et al.* [Summary of China Cardiovascular Disease Report 2016]. *Chin J Circulation* 2017; 32. [In Chinese].
37 ACCORD Study Group, Cushman WC, Evans GW, *et al*. Effects of intensive blood-pressure control in type 2 diabetes mellitus. *N Engl J Med* 2010; 362: 1575–1585.
38 SPRINT Research Group, Wright JT Jr, Williamson JD, *et al*. A randomized trial of intensive versus standard blood-pressure control. *N Engl J Med* 2015; 373: 2103–2116.
39 Wang JG, Staessen JA, Gong L, *et al*. Chinese trial on isolated systolic hypertension in the elderly. Systolic Hypertension in China (Syst-China) Collaborative Group. *Arch Intern Med* 2000; 160: 211–220.
40 Liu L, Zhang Y, Liu G, *et al*. The Felodipine Event Reduction (FEVER) Study: a randomized long-term placebo-controlled trial in Chinese hypertensive patients. *J Hypertens* 2005; 23: 2157–2172.
41 Huo Y, Li J, Qin X, *et al*. Efficacy of folic acid therapy in primary prevention of stroke among adults with hypertension in China: the CSPPT randomized clinical trial. *JAMA* 2015; 313: 1325–1335.
42 Kato N, Takeuchi F, Tabara Y, *et al*. Meta-analysis of genome-wide association studies identifies common variants associated with blood pressure in East Asians. *Nat Genet* 2011; 43: 531–538.
43 Gao PJ, Zhang KX, Zhu DL, *et al*. Diagnosis of Liddle syndrome by genetic analysis of β and γ subunits of epithelial sodium channel-a report of five affected family members. *J Hypertens* 2001; 19: 885–889.
44 Mulatero P, Morello F, Veglio F, *et al*. Genetics of primary aldosteronism. *J Hypertens* 2004; 22: 663–670.
45 Parati G, Stergiou G, O'Brien E, *et al*. European Society of Hypertension practice guidelines for ambulatory blood pressure monitoring. *J Hypertens* 2014; 32: 1359–1366.
46 Stergiou GS, Siontis KC, Ioannidis JP, *et al*. Home blood pressure as a cardiovascular outcome predictor: it's time to take this method seriously. *Hypertension* 2010; 55: 1301–1303.
47 Myers MG, Godwin M. Automated office blood pressure. *Can J Cardiol* 2012; 28: 341–346.
48 Parati G, Stergiou GS, Asmar R, *et al*. European Society of Hypertension guidelines for blood pressure monitoring at home: a summary report of the Second International Consensus Conference on Home Blood Pressure Monitoring. *J Hypertens* 2008; 26: 1505–1526.
49 Chinese Medical Association Hypertension Professional Committee. [Family blood pressure monitoring Chinese expert consensus]. *Chin Clin* 2012: 69–72. [In Chinese].
50 Schillaci G, Battista F, Pucci G, *et al*. A review of the role of electrocardiography in the diagnosis of left ventricular hypertrophy in hypertension. *J Electrocardiol* 2012; 45: 617–623.
51 Agrawal B, Berger A, Wolf K, *et al*. Microalbuminuria screening by reagent strip predicts cardiovascular risk in hypertension. *J Hypertens* 1996; 14: 223–228.
52 Levey AS, Stevens LA, Schmid CH, *et al*. A new equation to estimate glomerular filtration rate. *Ann Intern Med* 2009; 150: 604–612.
53 Levey AS, Bosch JP, Lewis JB, *et al*. A more accurate method to estimate glomerular filtration rate from serum creatinine: a new prediction equation. Modification of Diet in Renal Disease Study Group. *Ann Intern Med* 1999; 130: 461–470.
54 Ma YC, Zuo L, Chen JH, *et al*. Modified glomerular filtration rate estimating equation for Chinese patients with chronic kidney disease. *J Am Soc Nephrol* 2006; 17: 2937–2944.
55 Bos MJ, Koudstaal PJ, Hofman A, *et al*. Uric acid is a risk factor for myocardial infarction and stroke: the Rotterdam study. *Stroke* 2006; 37: 1503–1507.
56 O'Leary DH, Polak JF, Kronmal RA, *et al*. Carotid-artery intima and media thickness as a risk factor for myocardial infarction and stroke in older adults. Cardiovascular Health Study Collaborative Research Group. *N Engl J Med* 1999; 340: 14–22.
57 Polak JF, Pencina MJ, Pencina KM, *et al*. Carotid-wall in-

tima-media thickness and cardiovascular events. *N Engl J Med* 2011; 365: 213–221.
58 Vlachopoulos C, Aznaouridis K, Stefanadis C, *et al*. Prediction of cardiovascular events and all-cause mortality with arterial stiffness: a systematic review and meta-analysis. *J Am Coll Cardiol* 2010; 55: 1318–1327.
59 Laurent S, Cockcroft J, Van Bortel L, *et al*. Expert consensus document on arterial stiffness: methodological issues and clinical applications. *Eur Heart J* 2006; 27: 2588–2605.
60 Murabito JM, Evans JC, Larson MG, *et al*. The ankle-brachial index in the elderly and risk of stroke, coronary disease, and death: the Framingham Study. *Arch Intern Med* 2003; 163: 1939–1942.
61 Breslin DJ, Gifford RW, Jr., Fairbairn JF, *et al*. Prognostic importance of ophthalmoscopic findings in essential hypertension. *JAMA* 1966; 195: 335–338.
62 Lehmann MV, Schmieder RE. Remodeling of retinal small arteries in hypertension. *Am J Hypertens* 2011; 24: 1267–1273.
63 Longstreth WT Jr, Manolio TA, Arnold A, *et al*. Clinical correlates of white matter findings on cranial magnetic resonance imaging of 3301 elderly people. The Cardiovascular Health Study. *Stroke* 1996; 27: 1274–1282.
64 Revision Committee of the Guidelines for the Prevention and Treatment of Hypertension in China. [Guidelines for the Prevention and Treatment of Hypertension in China]. Revised 2005, Beijing: People's Medical Publishing House; 2006. [In Chinese].
65 [China's Guidelines for the Prevention and Treatment of Hypertension]. Committee on Prevention and Treatment of Hypertension in China. Revised 2010, Beijing: People's Medical Publishing House; 2012. [In Chinese].
66 Zhang Y, Zhang X, Liu L, *et al*. Is a systolic blood pressure target < 140 mmHg indicated in all hypertensives? Subgroup analyses of findings from the randomized FEVER trial. *Euro Heart J* 2011; 32: 1500–1508.
67 Lonn EM, Bosch J, Lopez-Jaramillo P, *et al*. Blood-pressure lowering in intermediate-risk persons without cardiovascular disease. *N Engl J Med* 2016; 374: 2009–2020.
68 Fox KM. Efficacy of perindopril in reduction of cardiovascular events among patients with stable coronary artery disease: randomised, double-blind, placebo-controlled, multicentre trial (the EUROPA study). *Lancet* 2003; 362: 782–788.
69 Staessen JA, Fagard R, Thijs L, *et al*. Randomised double-blind comparison of placebo and active treatment for older patients with isolated systolic hypertension. The Systolic Hypertension in Europe (Syst-Eur) Trial Investigators. *Lancet* 1997; 350: 757–764.
70 Beckett NS, Peters R, Fletcher AE, *et al*. Treatment of hypertension in patients 80 years of age or older. *N Engl J Med* 2008; 358: 1887–1898.
71 Collins R, Peto R, MacMahon S, *et al*. Blood pressure, stroke, and coronary heart disease. Part 2, Short-term reductions in blood pressure: overview of randomised drug trials in their epidemiological context. *Lancet* 1990; 335: 827–838.
72 Staessen JA, Gasowski J, Wang JG, *et al*. Risks of untreated and treated isolated systolic hypertension in the elderly: meta-analysis of outcome trials. *Lancet* 2000; 355: 865–872.
73 Elliott P, Stamler J, Nichols R, *et al*. Intersalt revisited: further analyses of 24 hour sodium excretion and blood pressure within and across populations. Intersalt Cooperative Research Group. *BMJ* 1996; 312: 1249–1253.
74 Aburto NJ, Hanson S, Gutierrez H, *et al*. Effect of increased potassium intake on cardiovascular risk factors and disease: systematic review and meta-analyses. *BMJ* 2013; 346: f1378.
75 Sacks FM, Svetkey LP, Vollmer WM, *et al*. Effects on blood pressure of reduced dietary sodium and the Dietary Approaches to Stop Hypertension (DASH) diet. DASH-Sodium Collaborative Research Group. *N Engl J Med* 2001; 344: 3–10.
76 Zhao Q, Gu D, Chen J, *et al*. Blood pressure responses to dietary sodium and potassium interventions and the cold pressor test: the GenSalt replication study in rural North China. *Am J Hypertens* 2014; 27: 72–80.
77 Mozaffarian D, Fahimi S, Singh GM, *et al*. Global sodium consumption and death from cardiovascular causes. *N Engl J Med* 2014; 371: 624–634.
78 CSSSC G. Salt substitution: a low-cost strategy for blood pressure control among rural Chinese. A randomized, controlled trial. *J Hypertens* 2007; 25: 2011–2018.
79 ICR G. Intersalt: an international study of electrolyte excretion and blood pressure. Results for 24 hour urinary sodium and potassium excretion. Intersalt Cooperative Research Group. *BMJ* 1988; 297: 319–328.
80 Appel LJ, Moore TJ, Obarzanek E, *et al*. A clinical trial of the effects of dietary patterns on blood pressure. DASH Collaborative Research Group. *N Engl J Med* 1997; 336: 1117–1124.
81 Saneei P, Salehi-Abargouei A, Esmaillzadeh A, *et al*. Influence of Dietary Approaches to Stop Hypertension (DASH) diet on blood pressure: a systematic review and meta-analysis on randomized controlled trials. *NMCD* 2014; 24: 1253–1261.
82 Struijk EA, May AM, Wezenbeek NL, *et al*. Adherence to dietary guidelines and cardiovascular disease risk in the EPIC-NL cohort. *Inter J Cardiol* 2014; 176: 354–359.
83 Fung TT, Chiuve SE, McCullough ML, *et al*. Adherence to a DASH-style diet and risk of coronary heart disease and stroke in women. *Arch Intern Med* 2008; 168: 713–720.
84 Neter JE, Stam BE, Kok FJ, *et al*. Influence of weight reduction on blood pressure: a meta-analysis of randomized controlled trials. *Hypertension* 2003; 42: 878–884.
85 China Obesity Working Group Data Summary Analysis Collaborative Group. [Predictive value of adult body mass index and waist circumference for risk factors associated with disease: a study of appropriate body mass index and waist circumference]. *Chin J Epidemiol* 2002; 23: 5–10. [In Chinese].
86 Aucott L, Rothnie H, McIntyre L, *et al*. Long-term weight

loss from lifestyle intervention benefits blood pressure?: a systematic review. *Hypertension* 2009; 54: 756–762.
87 Chen Z, Peto R, Zhou M, *et al*. Contrasting male and female trends in tobacco-attributed mortality in China: evidence from successive nationwide prospective cohort studies. *Lancet* 2015; 386: 1447–1456.
88 Clair C, Rigotti NA, Porneala B, *et al*. Association of smoking cessation and weight change with cardiovascular disease among adults with and without diabetes. *JAMA* 2013; 309: 1014–1021.
89 Peng M, Wu S, Jiang X, *et al*. Long-term alcohol consumption is an independent risk factor of hypertension development in northern China: evidence from Kailuan study. *J Hypertens* 2013; 31: 2342–2347.
90 Zhao J, Stockwell T, Roemer A, *et al*. Alcohol consumption and mortality from coronary heart disease: an updated meta-analysis of cohort studies. *J Stud Alcohol Drugs* 2017; 78: 375–386.
91 Cushman WC, Cutler JA, Hanna E, *et al*. Prevention and Treatment of Hypertension Study (PATHS): effects of an alcohol treatment program on blood pressure. *Arch Intern Med* 1998; 158: 1197–1207.
92 Tanasescu M, Leitzmann MF, Rimm EB, *et al*. Exercise type and intensity in relation to coronary heart disease in men. *JAMA* 2002; 288: 1994–2000.
93 Engstrom G, Hedblad B, Janzon L, *et al*. Hypertensive men who exercise regularly have lower rate of cardiovascular mortality. *J Hypertens* 1999; 17: 737–742.
94 Whelton SP, Chin A, Xin X, *et al*. Effect of aerobic exercise on blood pressure: a meta-analysis of randomized, controlled trials. *Ann Intern Med* 2002; 136: 493–503.
95 Landsbergis PA, Dobson M, Koutsouras G, *et al*. Job strain and ambulatory blood pressure: a meta-analysis and systematic review. *Am J Public Health* 2013; 103: e61–e71.
96 Elmer PJ, Obarzanek E, Vollmer WM, *et al*. Effects of comprehensive lifestyle modification on diet, weight, physical fitness, and blood pressure control: 18-month results of a randomized trial. *Ann Intern Med* 2006; 144: 485–495.
97 Cook NR, Cutler JA, Obarzanek E, *et al*. Long term effects of dietary sodium reduction on cardiovascular disease outcomes: observational follow-up of the trials of hypertension prevention (TOHP). *BMJ* 2007; 334: 885–888.
98 Dickinson HO, Mason JM, Nicolson DJ, *et al*. Lifestyle interventions to reduce raised blood pressure: a systematic review of randomized controlled trials. *J Hypertens* 2006; 24: 215–233.
99 He J, Whelton PK, Appel LJ, *et al*. Long-term effects of weight loss and dietary sodium reduction on incidence of hypertension. *Hypertension* 2000; 35: 544–549.
100 Weber MA, Schiffrin EL, White WB, *et al*. Clinical practice guidelines for the management of hypertension in the community: a statement by the American Society of Hypertension and the International Society of Hypertension. *J Clin Hypertens (Greenwich)* 2014; 16: 14–26.
101 The Hypertension Group in Chinese Society of Cardiology. [2015 Chinese expert guidance on the management of salt intake to control hypertension]. *Chin J Hypertens* 2015; 23: 1028–1034. [In Chinese].
102 Chen X, Guo X, Ma J, *et al*. Urinary sodium or potassium excretion and blood pressure in adults of Shandong province, China: preliminary results of the SMASH project. *JASH* 2015; 9: 754–762.
103 Anderson CA, Appel LJ, Okuda N, *et al*. Dietary sources of sodium in China, Japan, the United Kingdom, and the United States, women and men aged 40 to 59 years: the INTERMAP study. *J Am Diet Assoc* 2010; 110: 736–745.
104 China Obesity Working Group Data Summary Analysis Collaborative Group. [Predictive value of Chinese adult body mass index and waist circumference for risk factors of related diseases: a study of appropriate body mass index and waist cut point]. *Chin J Epidemiol* 2003; 23: 5–10. [In Chinese].
105 Department of Disease Control, Ministry of Health of the People's Republic of China. [Guidelines for Prevention and Control of Overweight and Obesity in Adults in China]. Beijing: People's Medical Publishing House, 2006. [In Chinese].
106 Douketis JD, Macie C, Thabane L, *et al*. Systematic review of long-term weight loss studies in obese adults: clinical significance and applicability to clinical practice. *Int J obes (Lond)* 2005; 29: 1153–1167.
107 Wing RR, Lang W, Wadden TA, *et al*. Benefits of modest weight loss in improving cardiovascular risk factors in overweight and obese individuals with type 2 diabetes. *Diabetes Care* 2011; 34: 1481–1486.
108 Malek AM, Cushman M, Lackland DT, *et al*. Secondhand Smoke Exposure and Stroke: The Reasons for Geographic and Racial Differences in Stroke (REGARDS) Study. *Am J Prev Med* 2015; 49: e89–e97.
109 Primatesta P, Falaschetti E, Gupta S, *et al*. Association between smoking and blood pressure: evidence from the health survey for England. *Hypertension* 2001; 37: 187–193.
110 Mancia G, Fagard R, Narkiewicz K, *et al*. 2013 ESH/ESC Guidelines for the management of arterial hypertension: the Task Force for the management of arterial hypertension of the European Society of Hypertension (ESH) and of the European Society of Cardiology (ESC). *J Hypertens* 2013; 31: 1281–1357.
111 Leung AA, Nerenberg K, Daskalopoulou SS, *et al*. Hypertension Canada's 2016 Canadian Hypertension Education Program Guidelines for Blood Pressure Measurement, Diagnosis, Assessment of Risk, Prevention, and Treatment of Hypertension. *Can J Cardiol* 2016; 32: 569–588.
112 Okin PM, Oikarinen L, Viitasalo M, *et al*. Prognostic value of changes in the electrocardiographic strain pattern during antihypertensive treatment: the Losartan Intervention for

End-Point Reduction in Hypertension Study (LIFE). *Circulation* 2009; 119: 1883–1891.

113 Parving HH, Lehnert H, Brochner-Mortensen J, *et al*. The effect of irbesartan on the development of diabetic nephropathy in patients with type 2 diabetes. *N Engl J Med* 2001; 345: 870–878.

114 Zong WZ, Yang WY, Xiang HD, *et al.* [Efficacy and safety of irbesartan in the treatment of type 2 diabetes with albuminuria: a multicenter randomized double-blind controlled study]. *Chin J Endocrinol Metabol* 2008; 24: 55–58. [In Chinese].

115 Lewis EJ, Hunsicker LG, Clarke WR, *et al*. Renoprotective effect of the angiotensin-receptor antagonist irbesartan in patients with nephropathy due to type 2 diabetes. *N Engl J Med* 2001; 345: 851–860.

116 Department of Hypertension, Chinese Medical Association Cardiovascular Disease Branch. [Chinese expert guidance for early morning blood pressure clinical management]. *Chin J Cardiovasc Diseases* 2014; 42: 721–725. [In Chinese].

117 Brown MJ, Palmer CR, Castaigne A, *et al*. Morbidity and mortality in patients randomised to double-blind treatment with a long-acting calcium-channel blocker or diuretic in the International Nifedipine GITS study: Intervention as a Goal in Hypertension Treatment (INSIGHT). *Lancet* 2000; 356: 366–372.

118 Kario K, Saito I, Kushiro T, *et al*. Home blood pressure and cardiovascular outcomes in patients during antihypertensive therapy: primary results of HONEST, a large-scale prospective, real-world observational study. *Hypertension* 2014; 64: 989–996.

119 Weber MA, Julius S, Kjeldsen SE, *et al*. Blood pressure dependent and independent effects of antihypertensive treatment on clinical events in the VALUE Trial. *Lancet* 2004; 363: 2049–2051.

120 Wang W, Ma LY, Liu MB, *et al.* [Phase report on the effect of initial low-dose amlodipine plus telmisartan or compound amiloride on blood pressure control rate in patients with hypertension]. *Chin J Cardiovasc Diseases* 2009; 25: 701–707. [In Chinese].

121 Jamerson K, Weber MA, Bakris GL, *et al*. Benazepril plus amlodipine or hydrochlorothiazide for hypertension in high-risk patients. *New Engl J Med* 2008; 359: 2417–2428.

122 Patel A, MacMahon S, Chalmers J, *et al*. Effects of a fixed combination of perindopril and indapamide on macrovascular and microvascular outcomes in patients with type 2 diabetes mellitus (the ADVANCE trial): a randomised controlled trial. *Lancet* 2007; 370: 829–840.

123 Wang W, Ma L, Zhang Y, *et al*. The combination of amlodipine and angiotensin receptor blocker or diuretics in high-risk hypertensive patients: rationale, design and baseline characteristics. *J Hum Hypertens* 2011; 25: 271–277.

124 Staessen JA, Wang JG, Thijs L, *et al*. Cardiovascular protection and blood pressure reduction: a meta-analysis. *Lancet* 2001; 358: 1305–1315.

125 Neal B, MacMahon S, Chapman N, *et al*. Effects of ACE inhibitors, calcium antagonists, and other blood-pressure-lowering drugs: results of prospectively designed overviews of randomised trials. Blood Pressure Lowering Treatment Trialists' Collaboration. *Lancet* 2000; 356: 1955–1964.

126 Ogihara T, Saruta T, Rakugi H, *et al*. Target blood pressure for treatment of isolated systolic hypertension in the elderly: valsartan in elderly isolated systolic hypertension study. *Hypertension* 2010; 56: 196–202.

127 Liu L, Wang JG, Gong L, *et al*. Comparison of active treatment and placebo in older Chinese patients with isolated systolic hypertension. Systolic Hypertension in China (Syst-China) Collaborative Group. *J Hypertens* 1998; 16: 1823–1829.

128 Gong L, Zhang W, Zhu Y, *et al*. Shanghai trial of nifedipine in the elderly (STONE). *J Hypertens* 1996; 14: 1237–1245.

129 Zhang TJ. [Hypertensive intervention trial a randomized controlled trial of nifedipine and placebo]. *Chin J Cardiovasc Diseases* 1994; 201–5. [In Chinese].

130 PATS Collaborating Group. Post-stroke antihypertensive treatment study. A preliminary result. *Chin Med J* 1995; 108: 710–717.

131 Liu L, Wang Z, Gong L, *et al*. Blood pressure reduction for the secondary prevention of stroke: a Chinese trial and a systematic review of the literature. *Hypertens Res* 2009; 32: 1032–1040.

132 PROGRESS Collaborative Group. Randomised trial of a perindopril-based blood-pressure-lowering regimen among 6,105 individuals with previous stroke or transient ischaemic attack. *Lancet* 2001; 358: 1033–1041.

133 Arima H, Anderson C, Omae T, *et al*. Perindopril-based blood pressure lowering reduces major vascular events in Asian and Western participants with cerebrovascular disease: the PROGRESS trial. *J Hypertens* 2010; 28: 395–400.

134 Arima H, Anderson C, Omae T, *et al*. Degree of blood pressure reduction and recurrent stroke: the PROGRESS trial. *J Neurol Neurosurg Psychiatry* 2014; 85: 1284–1285.

135 Liu LS, Gong LS, Wang W, *et al*. [A multicenter randomized, double-blind, controlled clinical trial of antihypertensive therapy for stroke recurrence in Chinese patients with cerebrovascular disease]. *Chin J Cardiovasc Diseases* 2005; 33: 613–617. [In Chinese].

136 Bostom AG, Rosenberg IH, Silbershatz H, *et al*. Nonfasting plasma total homocysteine levels and stroke incidence in elderly persons: the Framingham Study. *Ann Intern Med* 1999; 131: 352–355.

137 Zhou XL, Hu AH, Hui YT, *et al.* [Relationship between MTHFR gene polymorphism and plasma homocysteine levels and stroke]. *Chin J Cardiovasc Disease* 1999; 27: 121–123. [In Chinese].

138 Zhang W, Sun K, Chen J, *et al*. High plasma homocysteine

levels contribute to the risk of stroke recurrence and all cause mortality in a large prospective stroke population. *Clin Sci (Lond)* 2010; 118: 187–194.

139 Mark SD, Wang W, Fraumeni JF Jr, *et al*. Lowered risks of hypertension and cerebrovascular disease after vitamin/mineral supplementation: the Linxian Nutrition Intervention Trial. *Am J Epidemiol* 1996; 143: 658–664.

140 Zhao M, Wu G, Li Y, *et al*. Meta-analysis of folic acid efficacy trials in stroke prevention: Insight into effect modifiers. *Neurology* 2017; 88: 1830–1838.

141 Xu X, Qin X, Li Y, *et al*. Efficacy of folic acid therapy on the progression of chronic kidney disease: the renal substudy of the China stroke primary prevention trial. *JAMA Intern Med* 2016; 176: 1443–1450.

142 Qin X, Li Y, He M, *et al*. Folic acid therapy reduces serum uric acid in hypertensive patients: a substudy of the China Stroke Primary Prevention Trial (CSPPT). *Am J Clin Nutr* 2017; 105: 882–889.

143 Cooper-DeHoff RM, Gong Y, Handberg EM, *et al*. Tight blood pressure control and cardiovascular outcomes among hypertensive patients with diabetes and coronary artery disease. *JAMA* 2010; 304: 61–68.

144 Wang W, Wang JG, Zhang YQ, *et al*. [To formulate strategies and programs for prevention and treatment of hypertension in China according to the characteristics of hypertension in China]. *Chin J Hypertens* 2010; 904–907. [In Chinese].

145 Wang JG, Li Y, Franklin SS, *et al*. Prevention of stroke and myocardial infarction by amlodipine and Angiotensin receptor blockers: a quantitative overview. *Hypertension* 2007; 50: 181–188.

146 Wang JG, Kario K, Lau T, *et al*. Use of dihydropyridine calcium channel blockers in the management of hypertension in Eastern Asians: a scientific statement from the Asian Pacific Heart Association. *Hypertensi Res* 2011; 34: 423–430.

147 Mancia G, Parati G, Bilo G, *et al*. Blood pressure control by the nifedipine GITS-telmisartan combination in patients at high cardiovascular risk: the TALENT study. *J Hypertens* 2011; 29: 600–609.

148 Nissen SE, Tuzcu EM, Libby P, *et al*. Effect of antihypertensive agents on cardiovascular events in patients with coronary disease and normal blood pressure: the CAMELOT study: a randomized controlled trial. *JAMA* 2004; 292: 2217–2225.

149 Elliott HL, Meredith PA. Preferential benefits of nifedipine GITS in systolic hypertension and in combination with RAS blockade: further analysis of the 'ACTION' database in patients with angina. *J Hum Hypertens* 2011; 25: 63–70.

150 Experts Association of Angiotensin-Converting Enzyme Inhibitors Correctly Applied in Kidney Diseases. [Expert consensus on the correct application of angiotensin-converting enzyme inhibitors in kidney disease]. *Chin J Nephrol* 2006: 57– 58. [In Chinese].

151 Danchin N, Cucherat M, Thuillez C, *et al*. [Angiotensin-converting enzyme inhibitors in patients with coronary artery disease and absence of heart failure or left ventricular systolic dysfunction: an overview of long-term randomized controlled trials. *Arch Intern Med* 2006; 166: 787–796.

152 Yusuf S, Teo KK, Pogue J, *et al*. Telmisartan, ramipril, or both in patients at high risk for vascular events. *N Engl J Med* 2008; 358: 1547–1559.

153 Lindholm LH, Ibsen H, Dahlof B, *et al*. Cardiovascular morbidity and mortality in patients with diabetes in the losartan intervention for endpoint reduction in hypertension study (LIFE): a randomised trial against atenolol. *Lancet* 2002; 359: 1004–1010.

154 Coyle D, Rodby R, Soroka S, *et al*. Cost-effectiveness of irbesartan 300 mg given early versus late in patients with hypertension and a history of type 2 diabetes and renal disease: a Canadian perspective. *Clin Ther* 2007; 29: 1508–1523.

155 Park HC, Choi HY, Kim BS, *et al*. Antiproteinuric effect of losartan in non-diabetic renal disease is not dependent on ACE insertion/deletion polymorphism. *Blood Press Res* 2006; 29: 216–224.

156 Al Badarin FJ, Abuannadi MA, Lavie CJ, *et al*. Evidence-based diuretic therapy for improving cardiovascular prognosis in systemic hypertension. *Am J Cardiol* 2011; 107: 1178–1184.

157 Cleland JG, Coletta AP, Lammiman M, *et al*. Clinical trials update from the European Society of Cardiology meeting 2005: CARE-HF extension study, ESSENTIAL, CIBIS-III, S-ICD, ISSUE-2, STRIDE-2, SOFA, IMAGINE, PREAMI, SIRIUS-II and ACTIVE. *Eur J Heart Fail* 2005; 7: 1070–1075.

158 Bisoprolol Center Research Collaboration Group. [Effect of domestic bisoprolol on glucose metabolism in patients with type 2 diabetes mellitus]. *Chin J Intern Med* 2005; 503–505. [In Chinese].

159 Castagno D, Jhund PS, McMurray JJ, *et al*. Improved survival with bisoprolol in patients with heart failure and renal impairment: an analysis of the cardiac insufficiency bisoprolol study II (CIBIS-II) trial. *Eur J Heart Fail* 2010; 12: 607–616.

160 Wikstrand J, Warnold I, Tuomilehto J, *et al*. Metoprolol versus thiazide diuretics in hypertension. Morbidity results from the MAPHY Study. *Hypertension* 1991; 17: 579–588.

161 Haenni A, Lithell H. Moxonidine improves insulin sensitivity in insulin-resistant hypertensives. *J Hypertension Suppl* 1999; 17: S29–S35.

162 Brown MJ, McInnes GT, Papst CC, *et al*. Aliskiren and the calcium channel blocker amlodipine combination as an initial treatment strategy for hypertension control (ACCELERATE): a randomised, parallel-group trial. *Lancet* 2011; 377: 312–320.

163 Oparil S, Yarows SA, Patel S, *et al*.Efficacy and safety of combined use of aliskiren and valsartan in patients with hypertension: a randomised, double-blind trial. *Lancet* 2007; 370: 221–229.

164 Yarows A, Oparil S, Pate S, *et al*.Aliskiren and valsartan in stage 2 hypertension: subgroup analysis of a randomised, double-blind study. *Adv Ther* 2008; 25: 1288–1302.

165 Duprez DA, Munger MA, Botha J, *et al*. Aliskiren for geritric lowering of systolic hypertension: a randomized controlled trial. *J Hum Hypertens* 2010; 24: 600–608.

166 Franklin SS, Lopez VA, Wong ND, *et al*. Single versus combined blood pressure components and risk for cardiovascular disease: the Framingham Heart Study. *Circulation* 2009; 119: 243–250.

167 Wang HY, Sun NL, Jing S, *et al*. [Efficacy and safety of compound reserpine triamterene tablets (Bump No. 0) and indapamide in the treatment of patients with essential hypertension Randomized controlled clinical study]. *Chin J Hypertens* 2016; 857–862. [In Chinese].

168 Jiang XJ, Gao RL. Post-study era of SIMPLICITY HTN 3: The status and challenges of denervation for the treatment of refractory hypertension. *Chin J Med* 2014; 1761–1763.

169 White WB, Galis ZS, Henegar J, *et al*. Renal denervation therapy for hypertension: pathways for moving development forward. *J Am Soc Hypertens* 2015; 9: 341–350.

170 Townsend RR, Mahfoud F, Kandzari DE, *et al*. Catheter-based renal denervation in patients with uncontrolled hypertension in the absence of antihypertensive medications (SPYRAL HTN-OFF MED): a randomised, sham-controlled, proof-of-concept trial. *Lancet* 2017; 390: 2160–2170.

171 Kandzari DE, Bohm M, Mahfoud F, *et al*. Effect of renal denervation on blood pressure in the presence of antihypertensive drugs: 6-month efficacy and safety results from the SPYRAL HTN-ON MED proof-of-concept randomised trial. *Lancet* 2018; 391: 2346–2355.

172 Bisognano JD, Bakris G, Nadim MK, *et al*. Baroreflex activation therapy lowers blood pressure in patients with resistant hypertension: results from the double-blind, randomized, placebo-controlled rheos pivotal trial. *J Am Coll Cardiol* 2011; 58: 765–773.

173 Lobo MD, Sobotka PA, Stanton A, *et al*. Central arteriovenous anastomosis for the treatment of patients with uncontrolled hypertension (the ROX CONTROL HTN study): a randomised controlled trial. *Lancet* 2015; 385: 1634–1641.

174 Zhu JR, Gao RL, Zhao L, *et al*. [Guidelines for prevention and treatment of dyslipidemia in Chinese adults (revised 2016)]. *Chin J Circ* 2016; 937–953. [In Chinese].

175 Randomised trial of cholesterol lowering in 4444 patients with coronary heart disease: the Scandinavian Simvastatin Survival Study (4S). *Lancet* 1994; 344: 1383–1389.

176 Sacks FM, Pfeffer MA, Moye LA, *et al*. The effect of pravastatin on coronary events after myocardial infarction in patients with average cholesterol levels. Cholesterol and Recurrent Events Trial investigators. *N Engl J Med* 1996; 335: 1001–1009.

177 Prevention of cardiovascular events and death with pravastatin in patients with coronary heart disease and a broad range of initial cholesterol levels. *N Engl J Med* 1998; 339: 1349–1357.

178 Serruys PW, de Feyter P, Macaya C, *et al*. Fluvastatin for prevention of cardiac events following successful first percutaneous coronary intervention: a randomized controlled trial. *JAMA* 2002; 287: 3215–3222.

179 MRC/BHF Heart Protection Study of cholesterol lowering with simvastatin in 20,536 high-risk individuals: a randomised placebo-controlled trial. *Lancet* 2002; 360: 7–22.

180 Colhoun HM, Betteridge DJ, Durrington PN, *et al*. Primary prevention of cardiovascular disease with atorvastatin in type 2 diabetes in the Collaborative Atorvastatin Diabetes Study (CARDS): multicentre randomised placebo-controlled trial. *Lancet* 2004; 364: 685–696.

181 LaRosa JC, Grundy SM, Waters DD, *et al*. Intensive lipid lowering with atorvastatin in patients with stable coronary disease. *N Engl J Med* 2005; 352: 1425–1435.

182 Amarenco P, Bogousslavsky J, Callahan A, *et al*. High-dose atorvastatin after stroke or transient ischemic attack. *N Engl J Med* 2006; 355: 549–559.

183 Taylor F, Ward K, Moore TH, *et al*. Statins for the primary prevention of cardiovascular disease. The Cochrane database of systematic reviews 2011; Cd004816.

184 [China Cholesterol Education Program Experts Group on Prevention and Treatment of Dyslipidemia]. *Chin J Cardiovasc Diseases* 2016; 661–664. [In Chinese].

185 Kernan WN, Ovbiagele B, Black HR, *et al*. Guidelines for the prevention of stroke in patients with stroke and transient ischemic attack: a guideline for healthcare professionals from the American Heart Association/American Stroke Association. *Stroke* 2014; 45: 2160–2236.

186 Fowkes FG, Price JF, Stewart MC, *et al*. Aspirin for prevention of cardiovascular events in a general population screened for a low ankle brachial index: a randomized controlled trial. *JAMA* 2010; 303: 841–848.

187 Baigent C, Blackwell L, Collins R, *et al*. Aspirin in the primary and secondary prevention of vascular disease: collaborative meta-analysis of individual participant data from randomised trials. *Lancet* 2009; 373:1849–1860.

188 Smith SC Jr, Benjamin EJ, Bonow RO, *et al*. AHA/ACCF Secondary Prevention and Risk Reduction Therapy for Patients with Coronary and other Atherosclerotic Vascular Disease: 2011 update: a guideline from the American Heart Association and American College of Cardiology Foundation. *Circulation* 2011; 124: 2458–2473.

189 [Guidelines for the Prevention and Treatment of Type 2 Diabetes in China (2013 Edition)]. *Chin J Diabete* 2014: 2–42. [In Chinese].

190 Guirguis-Blake JM, Evans CV, Senger CA, *et al*. Aspirin for the primary prevention of cardiovascular events: a systematic evidence review for the U.S. Preventive Services Task Force. *Ann Intern Med* 2016; 164: 804–813.

191 Halvorsen S, Andreotti F, ten Berg JM, *et al*. Aspirin therapy in primary cardiovascular disease prevention: a position paper of the European Society of Cardiology working group on thrombosis. *J Am Coll Cardiol* 2014; 64: 319–327.
192 Bibbins-Domingo K. Aspirin use for the primary prevention of cardiovascular disease and colorectal cancer: U.S. Preventive services task force recommendation statement. *Ann Intern Med* 2016; 164: 836–845.
193 Whitlock EP, Burda BU, Williams SB, *et al*. Bleeding risks with aspirin use for primary prevention in adults: a systematic review for the U.S. Preventive Services Task Force. *Ann Intern Med* 2016; 164: 826–835.
194 [Clinical application of aspirin in atherosclerotic cardiovascular disease: Chinese expert consensus (2016)]. *Chin J Intern Med* 2017: 68–80. [In Chinese].
195 Standards of medical care in diabetes-2014. *Diabetes Care* 2014; 37: S14–S80.
196 Zinman B, Wanner C, Lachin JM, *et al*. Empagliflozin, cardiovascular outcomes, and mortality in Type 2 Diabetes. *N Engl J Med* 2015; 373: 2117–2128.
197 Neal B, Perkovic V, Mahaffey KW, *et al*. Canagliflozin and cardiovascular and renal events in Type 2 Diabetes. *N Engl J Med* 2017; 377: 644–657.
198 Marso SP, Daniels GH, Brown-Frandsen K, *et al*. Liraglutide and cardiovascular outcomes in Type 2 Diabetes. *N Engl J Med* 2016; 375: 311–322.
199 [Diabetes Branch of Chinese Medical Association. Guidelines for the prevention and treatment of type 2 Diabetes in China (2017 Edition)]. *Chin J Diabetes* 2018; 10: 4–67. [In Chinese].
200 Wachtell K, Hornestam B, Lehto M, *et al*. Cardiovascular morbidity and mortality in hypertensive patients with a history of atrial fibrillation: the Losartan Intervention For End Point Reduction in Hypertension (LIFE) study. *J Am Coll Cardiol* 2005; 45: 705–711.
201 Kirchhof P, Benussi S, Kotecha D, *et al*. 2016 ESC Guidelines for the management of atrial fibrillation developed in collaboration with EACTS. *Eur J Cardiothorac Surg* 2016; 50: e1–e88.
202 Schnabel RB, Yin X, Gona P, *et al*. 50 year trends in atrial fibrillation prevalence, incidence, risk factors, and mortality in the Framingham Heart Study: a cohort study. *Lancet* 2015; 386: 154–162.
203 Connolly SJ, Ezekowitz MD, Yusuf S, *et al*. Dabigatran versus warfarin in patients with atrial fibrillation. *N Engl J Med* 2009; 361: 1139–1151.
204 Patel MR, Mahaffey KW, Garg J, *et al*. Rivaroxaban versus warfarin in nonvalvular atrial fibrillation. *N Engl J Med* 2011; 365: 883–891.
205 Granger CB, Alexander JH, McMurray JJ, *et al*. Apixaban versus warfarin in patients with atrial fibrillation. *N Engl J Med* 2011; 365: 981–992.
206 Giugliano RP, Ruff CT, Braunwald E, *et al*. Edoxaban versus warfarin in patients with atrial fibrillation. *N Engl J Med* 2013; 369: 2093–2104.
207 Yu EY, Wan EY, Chan KH, *et al*. Evaluation of the quality of care of a multi-disciplinary Risk Factor Assessment and Management Programme for Hypertension (RAMP-HT). *BMC Fam Pract* 2015; 16: 71.
208 Wang HHX, Wong MCS, Tang JL, *et al.* Primary care-based lifestyle interventions on blood pressure and lipid profiles among Chinese subjects: a meta-analysis. World Congress of Cardiology Scientific Sessions; 2012.
209 Wang HHX, Wong MCS, Yan BP, *et al*. Effectiveness of lifestyle interventions in reducing cardiovascular risk factors among Chinese subjects in primary care setting: a systematic review. *Intern J Cardiol* 2011; 147: S32.
210 Wang HH, Wang JJ, Xu L, *et al*. 12 Effect of cardiovascular risk-based approach in primary care physician-led management for 19,400 hypertensive patients in southern China. *J Hypertens* 2012; 30: e4.
211 Zhou ZY, Pan YZ, Yi WD, *et al*. [Comparative analysis of multiple risk factors for metabolic syndrome in community population in Guiyang City]. *Chin J Hypertens* 2008; 937–940. [In Chinese].
212 Zheng L, Li J, Sun Z, *et al*. Relationship of blood pressure with mortality and cardiovascular events among hypertensive patients aged >/= 60 years in rural areas of China: a strobe-compliant study. *Medicine (Baltimore)* 2015; 94: e1551.
213 Aronow WS, Fleg JL, Pepine CJ, *et al*. ACCF/AHA 2011 expert consensus document on hypertension in the elderly: a report of the American College of Cardiology Foundation Task Force on Clinical Expert Consensus Documents. *Circulation* 2011; 123: 2434–2506.
214 Franklin SS, Jacobs MJ, Wong ND, *et al*. Predominance of isolated systolic hypertension among middle-aged and elderly US hypertensives: analysis based on National Health and Nutrition Examination Survey (NHANES) III. *Hypertension* 2001; 37: 869–874.
215 Kim NR, Kim HC. Prevalence and trends of isolated systolic hypertension among Korean adults: the Korea national health and nutrition examination survey, 1998–2012. *Korean Circ J* 2015; 45: 492–499.
216 Bobrie G, Chatellier G, Genes N, *et al*. Cardiovascular prognosis of "masked hypertension" detected by blood pressure self-measurement in elderly treated hypertensive patients. *JAMA* 2004; 291: 1342–1349.
217 Insua JT, Sacks HS, Lau TS, *et al*. Drug treatment of hypertension in the elderly: a meta-analysis. *Ann Intern Med* 1994; 121: 355–362.
218 Franklin SS, Wilkinson IB, McEniery CM, *et al*. Unusual hypertensive phenotypes: what is their significance? *Hypertension* 2012; 59: 173–178.
219 Prevention of stroke by antihypertensive drug treatment in

older persons with isolated systolic hypertension. Final results of the Systolic Hypertension in the Elderly Program (SHEP). SHEP Cooperative Research Group. *JAMA* 1991; 265: 3255–3264.

220 Dahlof B, Lindholm LH, Hansson L, *et al*. Morbidity and mortality in the Swedish Trial in Old Patients with Hypertension (STOP-Hypertension). *Lancet* 1991; 338: 1281–1285.

221 Coope J, Warrender TS. Randomised trial of treatment of hypertension in elderly patients in primary care. *Br Med J (Clin Res Ed)* 1986; 293: 1145–1151.

222 Hansson L, Lindholm LH, Ekbom T, *et al*. Randomised trial of old and new antihypertensive drugs in elderly patients: cardiovascular mortality and morbidity the Swedish Trial in Old Patients with Hypertension-2 study. *Lancet* 1999; 354: 1751–1756.

223 Lithell H, Hansson L, Skoog I, *et al*. The Study on Cognition and Prognosis in the Elderly (SCOPE): principal results of a randomized double-blind intervention trial. *J Hypertens* 2003; 21: 875–886.

224 National High Blood Pressure Education Program Working Group on High Blood Pressure in Children and Adolescents. The fourth report on the diagnosis, evaluation, and treatment of high blood pressure in children and adolescents. *Pediatrics* 2004; 114: 555–576.

225 Lurbe E, Agabiti-Rosei E, Cruickshank JK, *et al*. 2016 European Society of Hypertension guidelines for the management of high blood pressure in children and adolescents. *J Hypertens* 2016; 34: 1887–1920.

226 Dong B, Ma J, Wang HJ, *et al*. The association of overweight and obesity with blood pressure among Chinese children and adolescents. *Biomed Environ Sci* 2013; 26: 437–444.

227 Lo JC, Sinaiko A, Chandra M, *et al*. Prehypertension and hypertension in community-based pediatric practice. *Pediatrics* 2013; 131: e415–e424.

228 Koebnick C, Mohan Y, Li X, *et al*. Failure to confirm high blood pressures in pediatric care-quantifying the risks of misclassification. *J Clin Hypertens (Greenwich)* 2018; 20: 174–182.

229 Hu YM. Zhu FT. [Practical Pediatrics]. Beijing: People's Medical Publishing House; 2002. [In Chinese].

230 McNiece KL, Gupta-Malhotra M, Samuels J, *et al*. Left ventricular hypertrophy in hypertensive adolescents: analysis of risk by 2004 National High Blood Pressure Education Program Working Group staging criteria. *Hypertension* 2007; 50: 392–395.

231 Brady TM, Redwine KM, Flynn JT, *et al*. Screening blood pressure measurement in children: are we saving lives? *Pediatr Nephrol* 2014; 29: 947–950.

232 Litwin M, Niemirska A, Sladowska J, *et al*. Left ventricular hypertrophy and arterial wall thickening in children with essential hypertension. *Pediatr Nephrol* 2006; 21: 811–819.

233 Sinha MD, Reid CJ. Evaluation of blood pressure in children. *Curr Opin Nephrol Hypertens* 2007; 16: 577–584.

234 Assadi F. Relation of left ventricular hypertrophy to microalbuminuria and C-reactive protein in children and adolescents with essential hypertension. *Pediatr Cardiol* 2008; 29: 580–584.

235 Mitchell P, Cheung N, de Haseth K, *et al*. Blood pressure and retinal arteriolar narrowing in children. *Hypertension* 2007; 49: 1156–1162.

236 Chen X, Wang Y, Appel LJ, *et al*. Impacts of measurement protocols on blood pressure tracking from childhood into adulthood: a metaregression analysis. *Hypertension* 2008; 51: 642–649.

237 Zhang MM, Mi J, Wang W, *et al*. [A longitudinal control survey of blood pressure in 412 children in Beijing after 18 years]. *Chinese Journal of Evidence-Based Pediatrics* 2006; 187–192. [In Chinese].

238 Sundstrom J, Neovius M, Tynelius P, *et al*. Association of blood pressure in late adolescence with subsequent mortality: cohort study of Swedish male conscripts. *BMJ* 2011; 342: d643.

239 Yan Y, Hou D, Liu J, *et al*. Childhood body mass index and blood pressure in prediction of subclinical vascular damage in adulthood: Beijing blood pressure cohort. *J Hypertens* 2017; 35: 47–54.

240 Liang Y, Hou D, Shan X, *et al*. Cardiovascular remodeling relates to elevated childhood blood pressure: Beijing Blood Pressure Cohort Study. *Int J Cardiol* 2014; 177: 836–839.

241 Gray L, Lee IM, Sesso HD, *et al*. Blood pressure in early adulthood, hypertension in middle age, and future cardiovascular disease mortality: HAHS (Harvard Alumni Health Study). *J Am Coll Cardiol* 2011; 58: 2396–2403.

242 Flynn JT, Kaelber DC, Baker-Smith CM, *et al*. Clinical practice guideline for screening and management of high blood pressure in children and adolescents. *Pediatrics* 2017,140.

243 Fan H, Yan YK, Mi J, *et al*. [Reference standards for gender, age and height in Chinese children aged 3 to 17 years]. *Chin J Hypertens* 2017; 428–435. [In Chinese].

244 Fan H, Hou D, Liu J, *et al*. Performance of 4 definitions of childhood elevated blood pressure in predicting subclinical cardiovascular outcomes in adulthood. *J Clin Hypertens* 2018; 20.

245 Fan H, Yan YK, Mi J, *et al*. [Development of a simplified blood pressure standard for children aged 3 to 17 years in China]. *Chin J Hypertens* 2017; 436–440. [In Chinese].

246 Li S, Chen W. Identifying elevated blood pressure and hypertension in children and adolescents. *J Clin Hypertens (Greenwich)* 2018; 20: 515–517.

247 Geleijnse JM, Hofman A, Witteman JC, *et al*. Long-term effects of neonatal sodium restriction on blood pressure. *Hypertension* 1997; 29: 913–917.

248 Rocchini AP, Katch V, Anderson J, *et al*. Blood pressure in obese adolescents: effect of weight loss. *Pediatrics* 1988; 82: 16–23.

249 McCambridge TM, Benjamin HJ, Brenner JS, *et al*. Athletic

participation by children and adolescents who have systemic hypertension. *Pediatrics* 2010; 125: 1287–1294.

250 Simons-Morton DG, Hunsberger SA, Van Horn L, *et al*. Nutrient intake and blood pressure in the Dietary Intervention Study in Children. *Hypertension* 1997; 29: 930–936.

251 Magee LA, Pels A, Helewa M, *et al*. Diagnosis, evaluation, and management of the hypertensive disorders of pregnancy: executive summary. *J Obstet Gynaecol Can* 2014; 36: 416–441.

252 Cantwell R, Clutton-Brock T, Cooper G, *et al*. Saving Mothers' Lives: Reviewing maternal deaths to make motherhood safer: 2006-2008. The Eighth Report of the Confidential Enquiries into Maternal Deaths in the United Kingdom. *BJOG* 2011; 118: 1–203.

253 Hypertension in pregnancy. Report of the American College of Obstetricians and Gynecologists' Task Force on Hypertension in Pregnancy. *Obstet Gynecol* 2013; 122: 1122–1131.

254 Sibai BM. Chronic hypertension in pregnancy. *Obstet Gynecol* 2002; 100: 369–377.

255 Gilbert WM, Young AL, Danielsen B, *et al*. Pregnancy outcomes in women with chronic hypertension: a population-based study. *J Reprod Med* 2007; 52: 1046–1051.

256 Magee LA, von Dadelszen P, Rey E, *et al*. Less-tight versus tight control of hypertension in pregnancy. *N Engl J Med* 2015; 372: 407–417.

257 Bujold E, Roberge S, Lacasse Y, *et al*. Prevention of preeclampsia and intrauterine growth restriction with aspirin started in early pregnancy: a meta-analysis. *Obstet Gynecol* 2010; 116: 402–414.

258 Lubsen J, Wagener G, Kirwan BA, *et al*. Effect of long- acting nifedipine on mortality and cardiovascular morbidity in patients with symptomatic stable angina and hypertension: the ACTION trial. *J Hypertens* 2005; 23: 641–648.

259 Zhang J, Zhang YH. [Multi-center, prospective Chinese heart failure registration study – preliminary analysis of etiology, clinical features and treatment]. *Chin J Circ* 2015; 30: 413–416. [In Chinese].

260 Ettehad D, Emdin CA, Kiran A, *et al*. Blood pressure lowering for prevention of cardiovascular disease and death: a systematic review and meta-analysis. *Lancet* 2016; 387: 957–967.

261 Ponikowski P, Voors AA, Anker SD, *et al*. 2016 ESC Guidelines for the diagnosis and treatment of acute and chronic heart failure: The Task Force for the diagnosis and treatment of acute and chronic heart failure of the European Society of Cardiology (ESC)Developed with the special contribution of the Heart Failure Association (HFA) of the ESC. *Eur Heart J* 2016; 37: 2129–2200.

262 Chinese Medical Association Cardiovascular Branch, Editorial Board of Chinese Journal of Cardiovascular Diseases. [Guidelines for the diagnosis and treatment of heart failure in China 2014]. *Chin J Cardiovasc Diseases* 2014; 98–122. [In Chinese].

263 Hollenberg NK. The Antihypertensive and Lipid-Lowering Treatment to Prevent Heart Attack Trial (ALLHAT). Major outcomes in high-risk hypertensive patients randomized to angiotensin-converting enzyme inhibitor or calcium channel blocker vs diuretic. *Curr Hypertens Rep* 2003; 5: 183–185.

264 Leenen FH, Nwachuku CE, Black HR, *et al*. Clinical events in high-risk hypertensive patients randomly assigned to calcium channel blocker versus angiotensin-converting enzyme inhibitor in the antihypertensive and lipid-lowering treatment to prevent heart attack trial. *Hypertension* 2006; 48: 374–384.

265 Zhang W, Shi W, Liu Z, *et al*. A nationwide cross-sectional survey on prevalence, management and pharmacoepidemiology patterns on hypertension in Chinese patients with chronic kidney disease. *Sci Rep* 2016; 6: 38768.

266 Zheng Y, Cai GY, Chen XM, *et al*. Prevalence, awareness, treatment, and control of hypertension in the non-dialysis chronic kidney disease patients. *Chin Med J (Engl)* 2013; 126: 2276–2280.

267 Lin J, Ding JJ, Fu CS, *et al.* [A cross-sectional survey of hypertension in patients with chronic kidney disease]. *Chin J Nephrol* 2009; 11: 827– 831. [In Chinese].

268 Dhaybi OA, Bakris G. Mineralocorticoid antagonists in chronic kidney disease. *Curr Opin Nephrol Hypertens* 2017; 26: 50–55.

269 Robinson BM, Tong L, Zhang J, *et al*. Blood pressure levels and mortality risk among hemodialysis patients in the Dialysis Outcomes and Practice Patterns Study. *Kidney Int* 2012; 82: 570–580.

270 Liu J, Zhao D, Liu J, *et al.* Prevalence of diabetes mellitus in outpatients with essential hypertension in China: a cross- sectional study. *BMJ Open* 2013; 3: e003798.

271 de Boer IH, Bangalore S, Benetos A, *et al*. Diabetes and hypertension: a position statement by the American Diabetes Association. *Diabetes Care* 2017; 40: 1273–1284.

272 Emdin CA, Rahimi K, Neal B, *et al*. Blood pressure lowering in type 2 diabetes: a systematic review and meta-analysis. *JAMA* 2015; 313: 603–615.

273 Hansson L, Zanchetti A, Carruthers SG, *et al*. Effects of intensive blood-pressure lowering and low-dose aspirin in patients with hypertension: principal results of the Hypertension Optimal Treatment (HOT) randomised trial. HOT Study Group. *Lancet* 1998; 351: 1755–1762.

274 Gu D, Reynolds K, Wu X, *et al*. Prevalence of the metabolic syndrome and overweight among adults in China. *Lancet* 2005; 365: 1398–1405.

275 Xi B, He D, Hu Y, *et al*. Prevalence of metabolic syndrome and its influencing factors among the Chinese adults: the China Health and Nutrition Survey in 2009. *Prev Med* 2013; 57: 867–871.

276 Zhu ZM. [Metabolic Syndrome---Etiology Exploration and Clinical Practice. Prevention and Treatment of Metabolic Syndrome]. Beijing: People's Military Medical Press, 2005. [In Chinese].

277 Liu J, Zhao D, Wang W, *et al.* [The relationship between different components and combinations of metabolic syndrome and cardiovascular disease in 11 provinces of China]. *Chin J Epidemiol* 2008; 652–625. [In Chinese].
278 Xiao J, Wu C, Xu G, *et al*. Association of physical activity with risk of metabolic syndrome: findings from a cross-sectional study conducted in rural area, Nantong, China. *J Sports Sci* 2016; 34: 1839–1848.
279 Selvin E, Erlinger TP. Prevalence of and risk factors for peripheral arterial disease in the United States: results from the National Health and Nutrition Examination Survey, 1999-2000. *Circulation* 2004; 110: 738–743.
280 Norgren L, Hiatt WR, Dormandy JA, *et al*. Inter-Society Consensus for the Management of Peripheral Arterial Disease (TASC II). *Eur J Vasc Endovasc Surg* 2007; 33: S1–S75.
281 Kong LZ, Hu SS. China Cardiovascular Disease Report 2010. Beijing: China Encyclopedia Publishing House, 2010, 112–113. [In Chinese].
282 Lip GY, Makin AJ. Treatment of hypertension in peripheral arterial disease. *Cochrane Database Syst Rev* 2003; CD003075.
283 Singer DR, Kite A. Management of hypertension in peripheral arterial disease: does the choice of drugs matter? *Eur J Vasc Endovasc Surg* 2008; 35: 701–708.
284 European Stroke O, Tendera M, Aboyans V, *et al*. ESC Guidelines on the diagnosis and treatment of peripheral artery diseases: Document covering atherosclerotic disease of extracranial carotid and vertebral, mesenteric, renal, upper and lower extremity arteries: the Task Force on the Diagnosis and Treatment of Peripheral Artery Diseases of the European Society of Cardiology (ESC). *Eur Heart J* 2011; 32: 2851–2906.
285 Anderson JL, Halperin JL, Albert NM, *et al*. Management of patients with peripheral artery disease (compilation of 2005 and 2011 ACCF/AHA guideline recommendations): a report of the American College of Cardiology Foundation/American Heart Association Task Force on Practice Guidelines. *Circulation* 2013; 127: 1425–1443.
286 Calhoun DA, Jones D, Textor S, *et al*. Resistant hypertension: diagnosis, evaluation, and treatment. A scientific statement from the American Heart Association Professional Education Committee of the Council for High Blood Pressure Research. *Hypertension* 2008; 51: 1403–1419.
287 Denolle T, Chamontin B, Doll G, *et al*. Management of resistant hypertension: expert consensus statement from the French Society of Hypertension, an affiliate of the French Society of Cardiology. *J Hum Hypertens* 2016; 30: 657–663.
288 Williams B, MacDonald TM, Morant S, *et al*. Spironolactone versus placebo, bisoprolol, and doxazosin to determine the optimal treatment for drug-resistant hypertension (PATHWAY-2): a randomised, double-blind, crossover trial. *Lancet* 2015; 386: 2059–2068.
289 Oliveras A, Armario P, Clara A, *et al*. Spironolactone versus sympathetic renal denervation to treat true resistant hypertension: results from the DENERVHTA study—a randomized controlled trial. *J Hypertens* 2016; 34: 1863–1871.
290 Wu ZS, Zhu DL, Jiang XJ, *et al.* [The position and recommendations of the Chinese Hypertension League on percutaneous transluminal radiofrequency ablation of renal sympathetic nerve for the treatment of refractory hypertension]. *Chin J Hypertens* 2013; 419: 22–23. [In Chinese].
291 Ogihara T, Kikuchi K, Matsuoka H, *et al*. The Japanese Society of Hypertension Guidelines for the Management of Hypertension (JSH 2009). *Hypertens Res* 2009; 32: 3–107.
292 Chobanian AV, Bakris GL, Black HR, *et al*. The Seventh Report of the Joint National Committee on prevention, detection, evaluation, and treatment of high blood pressure: the JNC 7 report. *JAMA* 2003; 289: 2560–2572.
293 Wright JT Jr, Fine LJ, Lackland DT, *et al*. Evidence supporting a systolic blood pressure goal of less than 150 mm Hg in patients aged 60 years or older: the minority view. *Ann Intern Med* 2014; 160: 499–503.
294 Ezzati M, Oza S, Danaei G, *et al*. Trends and cardiovascular mortality effects of state-level blood pressure and uncontrolled hypertension in the United States. *Circulation* 2008; 117: 905–914.
295 Aronson S. Perioperative hypertensive emergencies. *Curr Hypertens Rep* 2014; 16: 448.
296 Getsios D, Wang Y, Stolar M, *et al*. Improved perioperative blood pressure control leads to reduced hospital costs. *Expert Opin Pharmacother* 2013; 14: 1285–1293.
297 Hanada S, Kawakami H, Goto T, *et al*. Hypertension and anesthesia. *Curr Opin Anaesthesiol* 2006; 19: 315–319.
298 Marik PE, Varon J. Perioperative hypertension: a review of current and emerging therapeutic agents. *J Clin Anesth* 2009; 21: 220–229.
299 Wang ZW, Yan H, Wang X, *et al*. [Comparative study on the effects of hypertension management in rural communities]. *J Med Res* 2015; 25–28. [In Chinese].
300 Wang Z, Wang X, Chen Z, *et al*. Hypertension control in community health centers across China: analysis of antihypertensive drug treatment patterns. *Am J Hypertens* 2014; 27: 252–259.
301 Wu ZS, Huo Y, Wang W, Zhao LY, Zhu DL on behalf of the "China High Blood Pressure Education Guide" Compilation Committee. [Chinese Hypertension Patients Education Guide]. *Chin J Hypertens* 2013: 1123–1149. [In Chinese].
302 Li N, Cheng WP, Yan ZT, *et al.* [Investigation and analysis of target organ damage in sleep apnea-related hypertension]. *Chin J Hypertens* 2011; 642–646. [In Chinese].
303 Li NF, Li HJ, Wang HM, *et al.* [Study of left ventricular structural damage in patients with primary aldosteronism]. *Chin J Endocrinol Metabol* 2012: 117–120. [In Chinese].
304 Li N, Ma X, Wang HM, *et al*. [Analysis of proteinuria in patients with primary aldosteronism]. *Chin J Hypertens* 2013; 249–252. [In Chinese].
305 Nan-fang LI, W-pC, Zhi-tao YAN, *et al*. Prevalence of target

organ damage in patients with obstructive sleep apnea-related hypertension. *Am J Hypertens* 2011; 24: 1345.

306 Wang L, Li NF, Zhou KM, *et al.* [Analysis of 628 cases of uncontrollable hypertension]. *Chin J Cardiol* 2009; 138–141. [In Chinese].

307 Peng M, Jiang XJ, Dong H, *et al*. Etiology of renal artery stenosis in 2047 patients: a single-center retrospective analysis during a 15-year period in China. *J Hum Hypertens* 2016; 30: 124–128.

308 [Expert Consensus Drafting Group of Vascular Diseases and Hypertension Branch of China International Exchange and Promotive Association for Medical and Health Care Chinese expert consensus on diagnosis and treatment of renal artery stenosis]. *Chin J Circ* 2017; 835–844. [In Chinese].

309 Jiang X, Peng M, Li B, *et al*. The efficacy of renal artery stent combined with optimal medical therapy in patients with severe atherosclerotic renal artery stenosis. *Curr Med Res Opin* 2016; 32: 3–7.

310 Li NF, Zhang LL, Yan ZT, *et al.* [Study on the detection rate of sleep apnea hypopnea syndrome in hypertensive patients with different body mass index]. *Chin J Cardiovasc Diseases* 2012; 40: 120–124. [In Chinese].

311 Yao X, Li N, Zhang Y, *et al*. Plasma aldosterone concentration is positively associated with pulse pressure in patients with primary hypertension. *Medicine (Baltimore)* 2015; 94: e614.

312 Ma X, Wang HM, Li J, *et al.* [The prevalence of metabolic syndrome in patients with primary aldosteronism]. *Chin J Endocrinol Metab* 2011; 724–728. [In Chinese].

313 Zhou M, Wang H, Zhu J, *et al.* Cause-specific mortality for 240 causes in China during 1990-2013: a systematic subnational analysis for the Global Burden of Disease Study 2013. *Lancet* 2016; 387: 251–272.

Supplement 1. Blood pressure references for children and adolescents aged 3 to 17 years old in China by sex and height.

Supplement Table 1-1. Blood pressure references for children and adolescents aged 3 to 17 years old in China, male

Age, year	Height percentile	Height range, cm	SBP, mmHg				DBP, mmHg			
			50th	90th	95th	99th	50th	90th	95th	99th
3	P_5	< 96	88	99	102	108	54	62	65	72
	P_{10}	96–97	88	100	103	109	54	63	65	72
	P_{25}	98–100	89	101	104	110	54	63	66	72
	P_{50}	101–103	90	102	105	112	54	63	66	73
	P_{75}	104–106	91	103	107	113	55	63	66	73
	P_{90}	107–108	92	104	107	114	55	63	66	73
	P_{95}	≥ 109	93	105	108	115	55	63	66	73
4	P_5	< 102	89	101	104	111	55	64	67	74
	P_{10}	102–104	90	102	105	111	55	64	67	74
	P_{25}	105–107	91	103	106	113	55	64	67	74
	P_{50}	108–110	92	104	108	114	56	64	67	74
	P_{75}	111–113	93	106	109	115	56	64	67	74
	P_{90}	114–116	94	107	110	117	56	65	68	75
	P_{95}	≥ 117	95	107	111	117	56	65	68	75
5	P_5	< 109	92	104	107	114	56	65	68	75
	P_{10}	109–110	92	104	107	114	56	65	68	75
	P_{25}	111–113	93	105	109	115	56	65	68	75
	P_{50}	114–117	94	106	110	117	57	65	69	76
	P_{75}	118–120	95	108	111	118	57	66	69	76
	P_{90}	121–123	96	109	112	119	58	67	70	77
	P_{95}	≥ 124	97	110	113	120	58	67	70	77
6	P_5	< 114	93	105	109	115	57	66	69	76
	P_{10}	114–116	94	106	110	116	57	66	69	76
	P_{25}	117–119	95	107	111	117	58	66	69	77
	P_{50}	120–123	96	108	112	119	58	67	70	78
	P_{75}	124–126	97	110	113	120	59	68	71	78
	P_{90}	127–129	98	111	115	121	59	69	72	79
	P_{95}	≥ 130	99	112	116	123	60	69	73	80
7	P_5	< 118	94	106	110	117	58	67	70	77
	P_{10}	118–120	95	107	111	118	58	67	70	78
	P_{25}	121–123	96	108	112	119	59	68	71	78
	P_{50}	124–127	97	110	113	120	59	68	72	79
	P_{75}	128–131	98	112	115	122	60	70	73	81
	P_{90}	132–135	100	113	117	124	61	71	74	82
	P_{95}	≥ 136	100	114	117	125	62	71	74	82
8	P_5	< 121	95	108	111	118	59	68	71	78
	P_{10}	121–123	95	108	112	119	59	68	71	79
	P_{25}	124–127	97	110	113	120	60	69	72	80
	P_{50}	128–132	98	111	115	122	61	70	73	81
	P_{75}	133–136	99	113	117	124	62	71	74	82
	P_{90}	137–139	101	114	118	125	62	72	75	83
	P_{95}	≥ 140	102	115	119	127	63	73	76	84

Schedule 1-1. Cont.

Age, year	Height percentile	Height range, cm	SBP, mmHg				DBP, mmHg			
			50th	90th	95th	99th	50th	90th	95th	99th
9	P_5	< 125	96	109	112	119	60	69	72	80
	P_{10}	125–128	96	109	113	120	60	69	73	80
	P_{25}	129–132	98	111	115	122	61	71	74	82
	P_{50}	133–137	99	113	117	124	62	72	75	83
	P_{75}	138–142	101	115	119	126	63	73	76	84
	P_{90}	143–145	102	116	120	128	64	73	77	85
	P_{95}	≥ 146	103	117	121	129	64	74	77	85
10	P_5	< 130	97	110	114	121	61	70	74	81
	P_{10}	130–132	98	111	115	122	62	71	74	82
	P_{25}	133–137	99	113	116	124	62	72	75	83
	P_{50}	138–142	101	115	119	126	63	73	77	85
	P_{75}	143–147	102	117	120	128	64	74	77	85
	P_{90}	148–151	104	118	122	130	64	74	77	86
	P_{95}	≥ 152	105	119	123	131	64	74	77	86
11	P_5	< 134	98	111	115	122	62	72	75	83
	P_{10}	134–137	99	112	116	124	63	72	76	84
	P_{25}	138–142	100	114	118	126	64	73	77	85
	P_{50}	143–148	102	116	120	128	64	74	78	86
	P_{75}	149–153	104	119	123	130	64	74	78	86
	P_{90}	154–157	106	120	124	132	64	74	78	86
	P_{95}	≥ 158	106	121	125	133	64	74	78	86
12	P_5	< 140	100	113	117	125	64	73	77	85
	P_{10}	140–144	101	115	119	126	64	74	78	86
	P_{25}	145–149	102	117	121	128	65	75	78	86
	P_{50}	150–155	104	119	123	131	65	75	78	86
	P_{75}	156–160	106	121	125	133	65	75	78	86
	P_{90}	161–164	108	123	127	135	65	75	78	87
	P_{95}	≥ 165	108	124	128	136	65	75	78	87
13	P_5	< 147	102	116	120	128	65	75	78	86
	P_{10}	147–151	103	117	121	129	65	75	78	87
	P_{25}	152–156	104	119	123	131	65	75	79	87
	P_{50}	157–162	106	121	125	133	65	75	79	87
	P_{75}	163–167	108	123	128	136	65	75	79	87
	P_{90}	168–171	110	125	130	138	66	76	79	87
	P_{95}	≥ 172	110	126	130	139	66	76	79	88
14	P_5	< 154	103	118	122	130	65	75	79	87
	P_{10}	154–157	104	119	124	132	65	75	79	87
	P_{25}	158–162	106	121	125	133	65	75	79	87
	P_{50}	163–167	108	123	128	136	65	75	79	87
	P_{75}	168–172	109	125	130	138	66	76	79	88
	P_{90}	173–176	111	127	131	140	66	76	80	88
	P_{95}	≥ 177	112	128	133	141	67	77	80	89

Schedule 1-1. Cont.

Age, year	Height percentile	Height range, cm	SBP, mmHg 50th	SBP 90th	SBP 95th	SBP 99th	DBP, mmHg 50th	DBP 90th	DBP 95th	DBP 99th
15	P_5	< 158	105	120	124	132	65	76	79	87
	P_{10}	158–161	106	121	125	133	65	76	79	87
	P_{25}	162–166	107	122	127	135	66	76	79	88
	P_{50}	167–170	109	124	128	137	66	76	80	88
	P_{75}	171–174	110	126	131	139	66	77	80	89
	P_{90}	175–178	112	128	132	141	67	77	81	89
	P_{95}	≥ 179	113	129	133	142	67	77	81	90
16	P_5	< 161	105	121	125	133	66	76	79	88
	P_{10}	161–164	106	121	126	134	66	76	79	88
	P_{25}	165–168	107	123	127	136	66	76	80	88
	P_{50}	169–172	109	125	129	138	66	76	80	88
	P_{75}	173–176	111	126	131	140	67	77	80	89
	P_{90}	177–179	112	128	133	141	67	77	81	90
	P_{95}	≥ 180	113	129	134	142	67	78	81	90
17	P_5	< 163	106	121	126	134	66	76	80	88
	P_{10}	163–165	107	122	126	135	66	76	80	88
	P_{25}	166–169	108	124	128	136	66	76	80	88
	P_{50}	170–173	109	125	130	138	67	77	80	89
	P_{75}	174–177	111	127	131	140	67	77	81	89
	P_{90}	178–180	112	129	133	142	67	78	81	90
	P_{95}	≥ 181	113	129	134	143	68	78	82	90

Supplement Table 1-2. Blood pressure references for children and adolescents aged 3 to 17 years old in China, female

Age, year	Height percentile	Height range, cm	SBP, mmHg 50th	SBP 90th	SBP 95th	SBP 99th	DBP, mmHg 50th	DBP 90th	DBP 95th	DBP 99th
3	P_5	< 95	87	99	102	108	55	63	67	74
	P_{10}	95–96	88	99	103	109	55	63	67	74
	P_{25}	97–99	88	100	103	110	55	64	67	74
	P_{50}	100–102	89	101	104	111	55	64	67	74
	P_{75}	103–105	90	102	105	112	55	64	67	74
	P_{90}	106–107	91	103	106	113	55	64	67	75
	P_{95}	≥ 108	91	103	107	113	56	64	67	75
4	P_5	< 101	89	101	105	111	56	64	67	75
	P_{10}	101–103	89	101	105	111	56	64	67	75
	P_{25}	104–106	90	102	106	112	56	64	67	75
	P_{50}	107–109	91	103	107	113	56	64	67	75
	P_{75}	110–112	92	104	107	114	56	65	68	75
	P_{90}	113–114	93	105	109	115	56	65	68	76
	P_{95}	≥ 115	93	105	109	115	56	65	68	76
5	P_5	< 108	91	103	106	113	56	65	68	76
	P_{10}	108–109	91	103	107	113	56	65	68	76
	P_{25}	110–112	92	104	107	114	56	65	68	76
	P_{50}	113–116	93	105	109	115	57	65	68	76
	P_{75}	117–119	93	106	109	116	57	66	69	77
	P_{90}	120–122	94	107	111	117	58	66	70	77
	P_{95}	≥ 123	95	108	111	118	58	67	70	78

Schedule 1-2. Cont.

Age, year	Height percentile	Height range, cm	SBP, mmHg				DBP, mmHg			
			50th	90th	95th	99th	50th	90th	95th	99th
6	P_5	< 113	92	104	108	115	57	65	69	76
	P_{10}	113–114	92	105	108	115	57	66	69	77
	P_{25}	115–118	93	106	109	116	57	66	69	77
	P_{50}	119–121	94	107	110	117	58	67	70	78
	P_{75}	122–125	95	108	112	118	58	67	71	79
	P_{90}	126–128	96	109	113	119	59	68	71	79
	P_{95}	≥ 129	97	110	114	121	59	69	72	80
7	P_5	< 116	93	105	109	115	57	66	69	77
	P_{10}	116–118	93	106	109	116	57	66	69	77
	P_{25}	119–122	94	107	110	117	58	67	70	78
	P_{50}	123–126	95	108	112	119	59	68	71	79
	P_{75}	127–130	96	109	113	120	59	69	72	80
	P_{90}	131–133	97	111	114	122	60	69	73	81
	P_{95}	≥ 134	98	112	115	122	61	70	73	82
8	P_5	< 120	94	106	110	116	58	67	70	78
	P_{10}	120–122	94	107	111	117	58	67	71	79
	P_{25}	123–126	95	108	112	119	59	68	71	79
	P_{50}	127–131	96	109	113	120	60	69	72	80
	P_{75}	132–135	98	111	115	122	61	70	73	82
	P_{90}	136–138	99	112	116	123	61	71	74	83
	P_{95}	≥ 139	100	113	117	124	62	71	75	83
9	P_5	< 124	95	108	111	118	59	68	71	79
	P_{10}	124–127	95	108	112	119	59	68	72	80
	P_{25}	128–132	97	110	113	120	60	69	73	81
	P_{50}	133–136	98	111	115	122	61	71	74	82
	P_{75}	137–141	100	113	117	124	62	72	75	84
	P_{90}	142–145	101	114	118	125	63	72	76	84
	P_{95}	≥ 146	102	115	119	126	63	73	76	85
10	P_5	< 130	96	109	113	120	60	69	73	81
	P_{10}	130–133	97	110	114	121	61	70	73	82
	P_{25}	134–138	99	112	116	123	62	71	75	83
	P_{50}	139–143	100	113	117	124	63	72	76	84
	P_{75}	144–147	101	115	119	126	63	73	76	85
	P_{90}	148–151	103	116	120	128	63	73	77	85
	P_{95}	≥ 152	103	117	121	129	64	73	77	86
11	P_5	< 136	98	112	115	122	62	71	75	83
	P_{10}	136–139	99	113	116	123	62	72	75	84
	P_{25}	140–144	101	114	118	125	63	73	76	85
	P_{50}	145–149	102	116	120	127	64	73	77	86
	P_{75}	150–154	103	117	121	128	64	74	77	86
	P_{90}	155–157	104	118	122	129	64	74	77	86
	P_{95}	≥ 158	104	118	122	130	64	74	77	86

Schedule 1-2. Cont.

Age, year	Height percentile	Height range, cm	SBP, mmHg				DBP, mmHg			
			**50th**	**90th**	**95th**	**99th**	**50th**	**90th**	**95th**	**99th**
12	P_5	< 142	100	113	117	124	63	73	76	85
	P_{10}	142–145	101	114	118	125	63	73	77	85
	P_{25}	146–150	102	116	120	127	64	74	77	86
	P_{50}	151–154	103	117	121	129	64	74	78	86
	P_{75}	155–158	104	118	122	130	64	74	78	87
	P_{90}	159–162	105	119	123	130	64	74	78	87
	P_{95}	≥ 163	105	119	123	131	64	74	78	87
13	P_5	< 147	101	115	119	126	64	74	77	86
	P_{10}	147–149	102	116	120	127	64	74	78	87
	P_{25}	150–153	103	117	121	128	64	74	78	87
	P_{50}	154–157	104	118	122	129	65	74	78	87
	P_{75}	158–161	105	119	123	130	65	74	78	87
	P_{90}	162–164	105	119	123	131	65	74	78	87
	P_{95}	≥ 165	105	119	123	131	65	75	78	87
14	P_5	< 149	102	116	120	127	65	74	78	87
	P_{10}	149–152	103	117	121	128	65	75	78	87
	P_{25}	153–155	104	118	122	129	65	75	78	87
	P_{50}	156–159	104	118	122	130	65	75	78	87
	P_{75}	160–163	105	119	123	130	65	75	78	87
	P_{90}	164–166	105	119	123	131	65	75	79	87
	P_{95}	≥ 167	106	120	124	131	65	75	79	88
15	P_5	< 151	103	116	120	128	65	75	79	87
	P_{10}	151–152	103	117	121	128	65	75	79	88
	P_{25}	153–156	104	118	122	129	65	75	79	88
	P_{50}	157–160	105	119	123	130	65	75	79	88
	P_{75}	161–163	105	119	123	131	65	75	79	88
	P_{90}	164–166	105	120	124	131	65	75	79	88
	P_{95}	≥ 167	106	120	124	131	65	75	79	88
16	P_5	< 151	103	117	121	128	65	75	79	88
	P_{10}	151–153	103	117	121	129	65	75	79	88
	P_{25}	154–157	104	118	122	130	65	75	79	88
	P_{50}	158–160	105	119	123	130	65	75	79	88
	P_{75}	161–164	105	119	123	131	66	76	79	88
	P_{90}	165–167	106	120	124	131	66	76	79	88
	P_{95}	≥ 168	106	120	124	132	66	76	79	88
17	P_5	< 152	103	117	121	129	66	76	79	88
	P_{10}	152–154	104	118	122	129	66	76	79	89
	P_{25}	155–157	104	118	122	130	66	76	80	89
	P_{50}	158–161	105	119	123	130	66	76	80	89
	P_{75}	162–164	105	119	124	131	66	76	80	89
	P_{90}	165–167	106	120	124	132	66	76	80	89
	P_{95}	≥ 168	106	120	124	132	66	76	80	89
